# A summer course in cancer for high school students-an update on lessons taught and lessons learned

**DOI:** 10.1186/s12909-024-06002-z

**Published:** 2024-09-17

**Authors:** Xzaviar K. Solone, Siddhi Chitre, Laura Falceto Font, Kimberly N. Espinoza Pereira, Kathryn Stofer, Dietmar W. Siemann

**Affiliations:** 1 Department of Molecular Genetics and Microbiology, University of Florida, Gainesville, FL USA; 2 Department of Gastroenterology, University of Florida, Gainesville, FL USA; 3 Department of Neurosurgery, University of Florida, Gainesville, FL USA; 4 Department of Hematology & Oncology, University of Florida, Gainesville, FL USA; 5 Department of Agricultural Education and Communication, University of Florida, Gainesville, FL USA; 6 Department of Radiation Oncology, Gainesville, FL USA; 7 University of Florida Health Cancer Center, Gainesville, FL USA

**Keywords:** Cancer, Cancer hallmarks, Teaching, High-school summer course, Therapeutics, COVID-19

## Abstract

**Background:**

Previous graduate students and postdoctoral associates from the University of Florida Health Cancer Center, in partnership with the University of Florida Student Science Training Program, implemented a cooperative learning curriculum, providing high school students with a broad overview of cancer topics over six weeks over the summer. To address discussions necessitated by the COVID-19 pandemic on student autonomy, we report lessons learned and outcomes of a cancer biology and therapeutic curriculum modified for a collaborative learning environment.

**Methods:**

This pre-post longitudinal observational study conducted in 2023 on a cancer biology and therapeutics course evaluated students’ knowledge retention and general awareness and opinions in cancer research. A structured survey was employed for data collection, using learning assessment surveys and the Likert scale ranging from 1 to 10, with 10 being highly likely.

**Results:**

Student performance tracked over a 7-year period indicated consistency in performance between years. Post-assessment analysis revealed significant improvements in student benchmark understanding, notably in their ability to define cancer in one sentence (*p* = 0.0407), identify cancer therapies (*p* = 0.0040), and recognize cancer hallmarks (*p* < 0.0001). An increased trend in median response to the likelihood of pursuing cancer research (*p* = 0.8793) and the possibility of pursuing cancer research (*p* = 0.4874) were also observed, although not statistically significant. Moreover, feedback from participating students indicated that “*the educational activities at the end of class (e.g.*,* escape room*,* case studies)*” and “*learning about cancer and getting to work in groups…*” the curriculum fostered a positive educational learning environment.

**Conclusion:**

Students generally retained the course material presented and upheld a positive perception of the course. Incorporating opportunities for peer-to-peer learning, especially when introducing or discussing complex issues like cancer, may benefit student autotomy.

**Supplementary Information:**

The online version contains supplementary material available at 10.1186/s12909-024-06002-z.

## Introduction

The University of Florida Student Science Training Program (UF-SSTP) is a seven-week residential research program for high-achieving students entering their senior year of high school. Under the mentorship of faculty members, students actively participate in ongoing research projects for 30 h per week, gaining hands-on experience in current research topics. Celebrating its 65th consecutive year, the UF-SSTP has a long-standing tradition of fostering interpersonal, leadership, professional communication, and organizational skills in its participants. With over 5,000 academically talented students worldwide having completed this rigorous summer residential research program since its inception in 1959, the UF-SSTP provides invaluable opportunities for young scholars to excel. Furthermore, they enroll in UF honors seminar classes created and organized by graduate students and postdoctoral associates to enhance their academic knowledge and skills further.

Since 2011, graduate students and postdoctoral associates at the University of Florida Health Cancer Center (UFHCC) have coordinated a “Cancer Biology and Therapeutics” course. This course has been a transformative experience for lecturers and high school students alike, providing teaching experience to the former while enriching the latter’s knowledge of cancer. In a previous publication by former instructors of this course [1], the instructors tracked students’ performance and discussed the changes over five years. During this period, they observed incremental improvements in cumulative grade averages. Moreover, the instructors assessed students’ knowledge before and after the course and observed significant increases in understanding benchmarks, particularly in basic cancer knowledge and potential therapeutic options.

Recent studies have indicated that the COVID-19 pandemic has significantly impacted medical education, specifically learning and teaching styles, although the long-term implications are still being evaluated [2, 3]. Other studies have shown a significant correlation between online cooperative learning and problem-solving ability, as well as learning satisfaction [4], and students acknowledged that group learning benefited skills related to communication, problem-solving, research, listening, and negotiation [5]. The COVID-19 pandemic has prompted remote learning, in which elements have emerged, which are beneficial for student autonomy. Studies have shown that incorporating traditional and remote education increases scholastic fulfillment while promoting student autonomy [6, 7]. With that in mind we updated the cancer biology and therapeutics course [1] to have elements that can work for both online and in-person seminars.

Here, we provide an update on monitoring student progress amid the shift from COVID-19-associated limitations in 2021 to resuming in-person lectures (2022–2023). We aim to address student autonomy by designing surveys aimed at assessing student performance following updates to the cancer biology and therapeutics course [1].

## Methods

### Data collection

Final grade averages were collected between 2017 and 2023. In 2017 and 2018, previous instructors collected cumulative grades on campus. In 2021 the course was taught virtually following COVID-19 restrictions, and the grades were collected. In 2022–2023, grades were collected from students participating on campus when they were allowed to return to in-person lectures. In 2023, we designed learning assessment surveys to gauge student retention in core cancer biology concepts and developed Likert surveys to assess students’ perceptions of cancer research and career outcomes.

### Meeting time and style

The course convened eleven times on Tuesdays and Thursdays, with each session lasting two hours, totaling 22 contact hours (Table 1). The class size ranged from 10 to 14 students. Each lecture was divided into two sections: Sect. 1 consisted of prepared lectures, during which students were encouraged to complete follow-along worksheets for engagement and study purposes. In Sect. 2, students engaged in team-based group activities that applied lecture material, including case studies and group assignments. In-lecture assignments were collected, graded, and returned. Students demonstrated their understanding of cancer’s clinical impact through conceptual case studies. They underwent assessment for learning retention via a final examination consisting of multiple-choice, short-answer, and essay questions.

**Table 1:** Course schedule and topics

	Date	Topic	Assignment
1	06/15	Central Dogma	Group Assignment
2	06/20	Cancer Hallmarks	Case Study Assignment
3	06/22	Oncogene and Tumor Suppressor	Group Assignment
4	06/27	Epigenetics and Cancer	Group Assignment
5	06/29	Introduction to Immunology	Case Study
6	07/04	No Class	No Class
7	07/06	Immunotherapies for Cancer	Game Choice Activity
8	07/11	Microbiome in Cancer	Group Assignment
9	07/13	Therapy & the Microbiome	Group Assignment
10	07/18	Escape Room/Review	Group Project
11	07/20	Careers in Science Panel	Final Assessment Opens
12	07/25	End of the Course Celebration	

### Student learning outcomes

Our approach merges both didactic learning modules with cooperative learning strategies to educate students on recent cancer advancements [7]. Additionally, we exposed students to various career paths through an interactive panel discussion encompassing fields such as epidemiology, law, consulting, biomedical sciences, engineering, physics, medicine, and academia. The assessment of learning benchmarks spread across four modules was conducted as follows: (1) Students were evaluated on their ability to develop a solid understanding of basic cancer biology terminology, including terms such as cancer, tumor, oncogene, and tumor suppressor. (2) Student assessment involved exploring the fundamental characteristics of cancer, known as hallmarks, and gaining insights into how cancer cells behave differently from normal cells. (3) Students were tasked with investigating the factors and mechanisms contributing to the development and transformation of cancer cells. (4) Student evaluation included the role of epigenetic regulation in cancer. (5) Students’ understanding of the relationship between the immune system and cancer was assessed, exploring how immune responses can impact disease progression. (6) Assessment involved the influence of the microbiome on cancer prognosis and examining how the diverse gut microbiome in our bodies can affect cancer outcomes. (7) Evaluation included students gaining knowledge about different types of cancer therapies, both traditional and innovative, and understanding their mechanisms of action in treating cancer. (8) Students were exposed to different career options in cancer. Further details about student learning objectives and assessment methods can be found in Table 2.

**Table 2:** Course alignment map

Fundamentals of cancer biology alignment map				
Course objective	Module objectives	Resources	Assessments assignments	
		Required	Required	Optional
Develop a solid understanding of basic cancer biology terminology, including terms like cancer, tumor, oncogene, and tumor suppressor.	Module 1: Molecular Tumor Biology and Overview of Cancer Hallmarks	In class follow along worksheets.	Students will be divided into two large groups, and a structured presentation will be delivered based on a predefined case study focusing on glioblastoma and chronic myeloid leukemia.	Comprehensive worksheet
		TBLs worksheets.		
Explore the fundamental characteristics of cancer, known as hallmarks, and gain insights into how cancer cells behave differently from normal cells.	Module 2: Tumor Suppressors, Oncogenes, and Epigenetic Regulation in Cancer	In class follow along worksheets.		
		Group worksheets.		
Investigate the factors and mechanisms that contribute to the development and transformation of cancer cells.	Module 3: Cancer Immunology and Immunotherapies for Cancer	In class follow along worksheets.		
		Group worksheets.		
Learn about the role of epigenetic regulation in cancer.	Module 4: Microbiomes’ Impact on Cancer Prognosis and Treatment	In class follow along worksheets.		
		Group worksheets.		
Understand the relationship between the immune system and cancer and how immune responses can affect the progression of the disease.	Final Assessment: Comprehensive timed quiz		The Comprehensive Assessment is a single-attempt, time-limited examination lasting 95 min and covering 20 questions. Each module consists of 5 questions, including three multiple-choice questions, one short-answer question, and one essay question.	Create a 5 to 10-minute presentation that covers a topic within Cancer Immunology (of your choosing) by the last day of class. This assignment can be done individually or with a group.
Introduce the gut microbiome in healthy and diseased states. Understand the role of pathogenic bacteria in cancer development, gain knowledge about different types of cancer therapies, both traditional and innovative, and understand how they work to treat cancer. Explore different therapeutic options used in the clinic to modulate the gut microbiome.				
Introduce students to different types of cancer therapies, both traditional and innovative, and understand their mechanisms of action in treating cancer.				
Expose students to different career options in cancer.				

### Course lecture design

The course incorporated several key cancer hallmarks [8] structured into four modules to provide smaller, more manageable units. This approach facilitated a more cohesive and interconnected learning experience, allowing students to build upon and establish connections between concepts as they progressed through each module. Each module was divided into two lectures.

#### Module 1: Molecular tumor biology and overview of cancer hallmarks

Module 1 provided a foundational understanding of the course and necessary background information on cancer hallmarks. The topics in lecture one (Introduction and Central Dogma) included deoxyribose nucleic acid (DNA) structure and synthesis, transcription, protein structure, protein translation, and mutations. To facilitate the team-based group activity, the students were divided into two groups to translate genetic codes. Lecture 2 (Cancer Hallmarks) discussed genomic instability, malignant transformation, oncogenes and tumor suppressors, angiogenic signaling, and cancer metastasis and invasion. The group learning activity comprised two case studies with the following objectives: understanding how to research epidermal growth factor receptor (EGFR) and Kirsten Rat Sarcoma Viral oncogene homologue (KRAS) inhibitors, interpreting survival graphs, comprehending computed tomography (CT) and positron emission tomography (PET) scans, and understanding mechanisms of drug resistance.

#### Module 2: Tumor suppressors, oncogenes, and epigenetic regulation in Cancer

This module was based on the following hallmarks: genomic instability and mutation, evading growth suppressors, sustaining proliferative signaling, and non-mutation epigenetic reprogramming [8]. This module was divided into two parts. The first lecture introduced common mutations observed in cancer, followed by how these mutations contribute to the molecular regulation and role of oncogenes and tumor suppressor genes. The first lecture was followed by a case study covering cancer patient diagnosis & treatment. Each case study had a prompt that required students to think critically and engage with their peers to come up with an answer. The second lecture focused on epigenetics in cancer, highlighting what epigenetics is, critical regulators of epigenetic markers, and the functional consequence of deregulated epigenetic markers in cancer, such as DNA methylation on gene expression. An interesting case study with a real-world scenario in a cancer setting followed this lecture. Case studies proceeded the lecture to introduce how to identify and clinically target dysregulated epigenetic regulators.

#### Module 3: Cancer immunology and immunotherapies for cancer

Cancer Immunology and Immunotherapies for Cancer was the third of four modules in the course and was based on the “Avoiding immune destruction” hallmark [8]. This section was divided into two lectures: “Introduction to Immunology” and “Immunotherapies for Cancer”. The first lecture provided background on the fundamentals of immunology and the relationship between the immune system and cancer. The second lecture, “Immunotherapies for Cancer,” was to provide an understanding of how we can exploit our immune system to create therapies against cancer. Following lecture 1, students engaged in a peer-led case study assignment to identify responses to novel immunotherapy. After lecture 2, students were divided into two teams and participated in a competitive exercise using open notes to assess their comprehensive knowledge of the two lectures.

#### Module 4: Microbiome’s impact on cancer prognosis and treatment

Module 4 was based on incorporating hallmark “polymorphic microbiome” [8] and therapeutic options. The module was split into two lectures: Microbiome in Cancer and Therapy and the Microbiome. The first lecture introduced the human gut microbiome, microbial dysbiosis, bacteria’s role in cancer, and the importance of a healthy lifestyle to maintain a healthy gut microbiome. The students engaged in interactive learning experiences, such as the Human Gut Game (HGG) [9], a group-based activity that simulates the complexities of the human gut microbiome, allowing them to explore how changes in microbial populations can affect health outcomes, following lecture 1. The second lecture broadened students’ understanding of cancer treatment strategies beyond conventional therapies (i.e., surgery, radiation, chemotherapy). Subsequently, the lecture transitioned to the limitations of those therapies and how the microbiome can be harnessed as a complementary treatment strategy for cancer. For the group learning session, students played an interactive game, “Mystery Box”, that quizzes the understanding of the lecture. Students formed two teams, and each answered 10 questions related to the topic taught during the lecture.

### Case study assignment

Students were divided into two cohorts, formed through a random selection process. The students were provided with a rubric outlining the following prompts: Prompt 1: Present the findings related to the development, efficacy, safety, and culmination of a novel drug candidate for glioblastoma in clinical phase 4 trials. Prompt 2: Demonstrate the process of developing a novel diagnostic method for Chronic Myeloid Leukemia (CML), from hypothesis formulation to its implementation in clinical practice. Both cohorts were required to incorporate elements of in vitro and in vivo studies, team collaboration, data analysis and interpretation, clinical trial designs, and clinical implementation and evaluation. Students’ presentations were to be 15 min, followed by 5 min of questions by the audience and instructors.

### Escape room review

Students participated in examining seven multipart interactive challenges, utilizing their lecture notes to apply a comprehensive understanding of the course material (Additional file 1). The learning objectives (Additional file 2) encompass foundational knowledge of cancer hallmarks. Additionally, students could participate in a case study aimed at the practical application of the principles governing cancer hallmarks (Additional file 3).

### Panel discussion

Students were assessed to gauge their current career interests, and the panelists were selected based on students’ feedback. The career panel was divided into two parts. The first part was a brief 10-minute presentation about potential careers in science, technology, engineering and mathematics (STEM) given by one of the course instructors. The second and central part was a moderated panel discussion between the different STEM professionals and the high school students. Panelists with expertise in various fields, including engineering, physics, biomedical sciences and research, medical practice, and venture capital and consulting, were invited to participate in the cancer-centric panel discussion.

More details of each module and course components are discussed in the extended methods (attachment 5).

### Final assessment

The comprehensive assessment was an online timed examination, allowing only a single attempt, with a duration of 95 min. Students were allotted five days to complete the assessment, which could be completed anywhere on campus. The assessment was designed to maintain academic integrity: (1) The questions were randomized for each student, and once the examination began, the students were to complete it within the allotted time; (2) once the assessment was finished, the students could not see the questions or the corrected answers until the close of the assessment. The assessment encompassed 20 questions, divided into four modules, each containing five questions. These questions were structured to include three multiple-choice questions, one short-answer question, and one essay question. Examination questions are included (Additional file 4).

## Results

### Cumulative grade averages

**Fig. 1:**
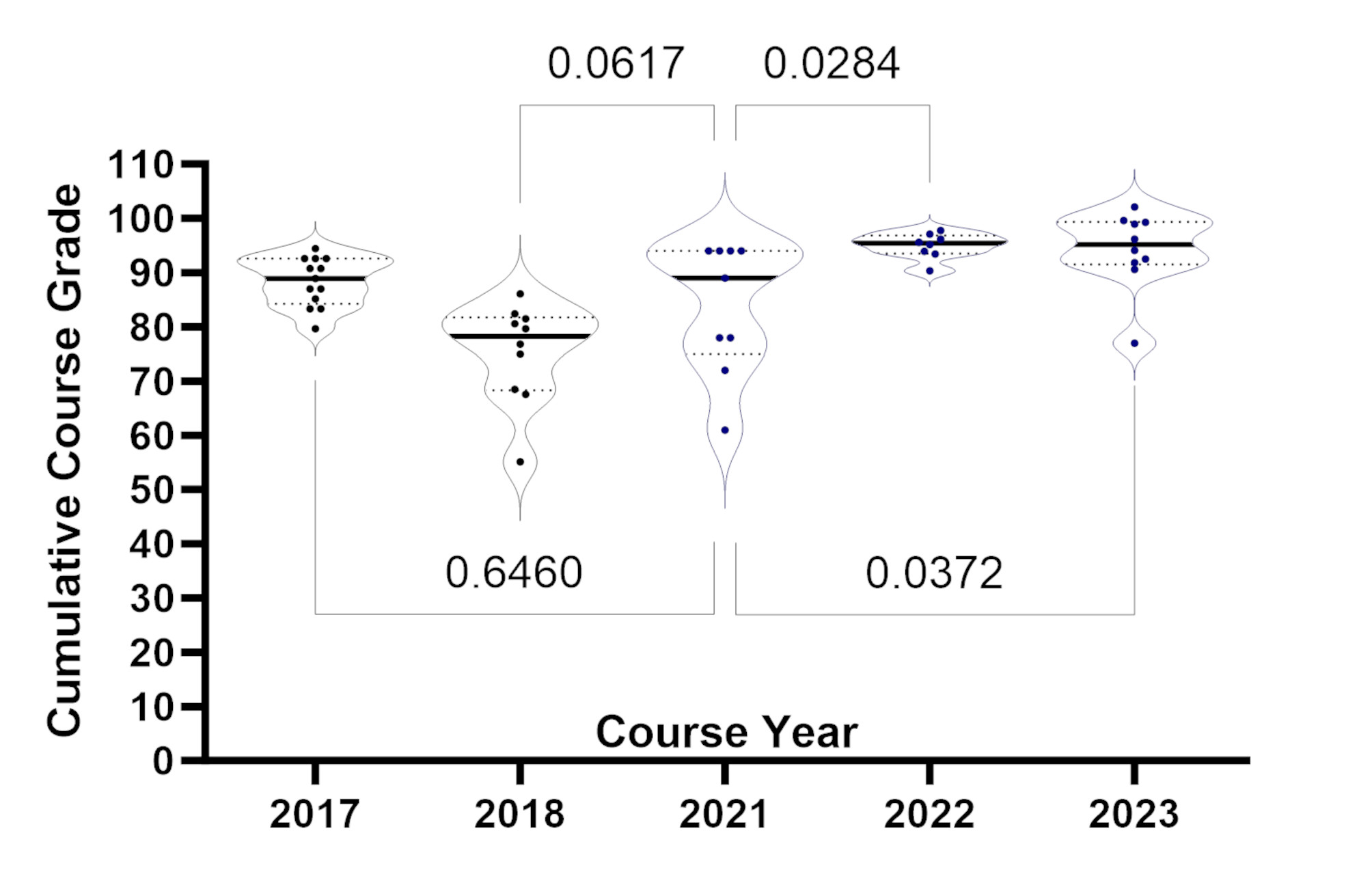
Cumulative course performance: Demonstrates the comparison of cumulative examination performance over different periods. Each data point represents an individual’s cumulative grade average. The examinations conducted in 2017–2018 were administered before the revision, while the 2021 examination was conducted virtually after the revision. Two-way ANOVA test was used to calculate the *p*-values

Trends in students’ final grade averages from 2017 to 2023 are depicted in Fig. 1, with individual grade averages tracked. Cumulative grade averages for 2017–2018 were collected by prior instructors as previously described [10]. The grades collected in 2021 reflect the modifications to the original course curriculum for virtual learning. Data for 2022 and 2023 represent grade averages after implementing changes to the curriculum (Fig. 1). Statistical analysis using students’ t-tests revealed no statistically significant deviation in student grade averages between 2017 and 2021. However, statistically significant improvements were observed from 2021 to 2022 (*p* = 0.0284) and 2023 (*p* = 0.0372). Each data point corresponds to the grade average of an individual student. We also observed improvement in the minimum grade average of 61 to 90.35 in 2022 and 76.98 in 2023 (Table 3).

**Table 3:** Frequency distribution and descriptive statistics of cumulative Grade averages

	2017	2018	2021	2022	2023
Total number of values	13	10	9	8	10
Minimum	79.63	55.09	61.00	90.35	76.98
25% Percentile	84.26	68.29	75.00	93.55	91.51
Median	88.89	78.24	89.00	95.40	95.15
75% Percentile	92.59	81.71	94.00	96.88	99.35
Maximum	94.44	86.11	94.00	97.81	102.15
Mean	88.32	75.32	83.78	94.95	94.21
Std. Deviation	4.50	9.24	12.09	2.37	7.17
Std. Error of Mean	1.25	2.92	4.03	0.84	2.27
Lower 95% CI of mean	85.60	68.72	74.48	92.96	89.08
Upper 95% CI of mean	91.04	81.93	93.07	96.93	99.34

### Pre and post questionnaire

**Fig. 2:**
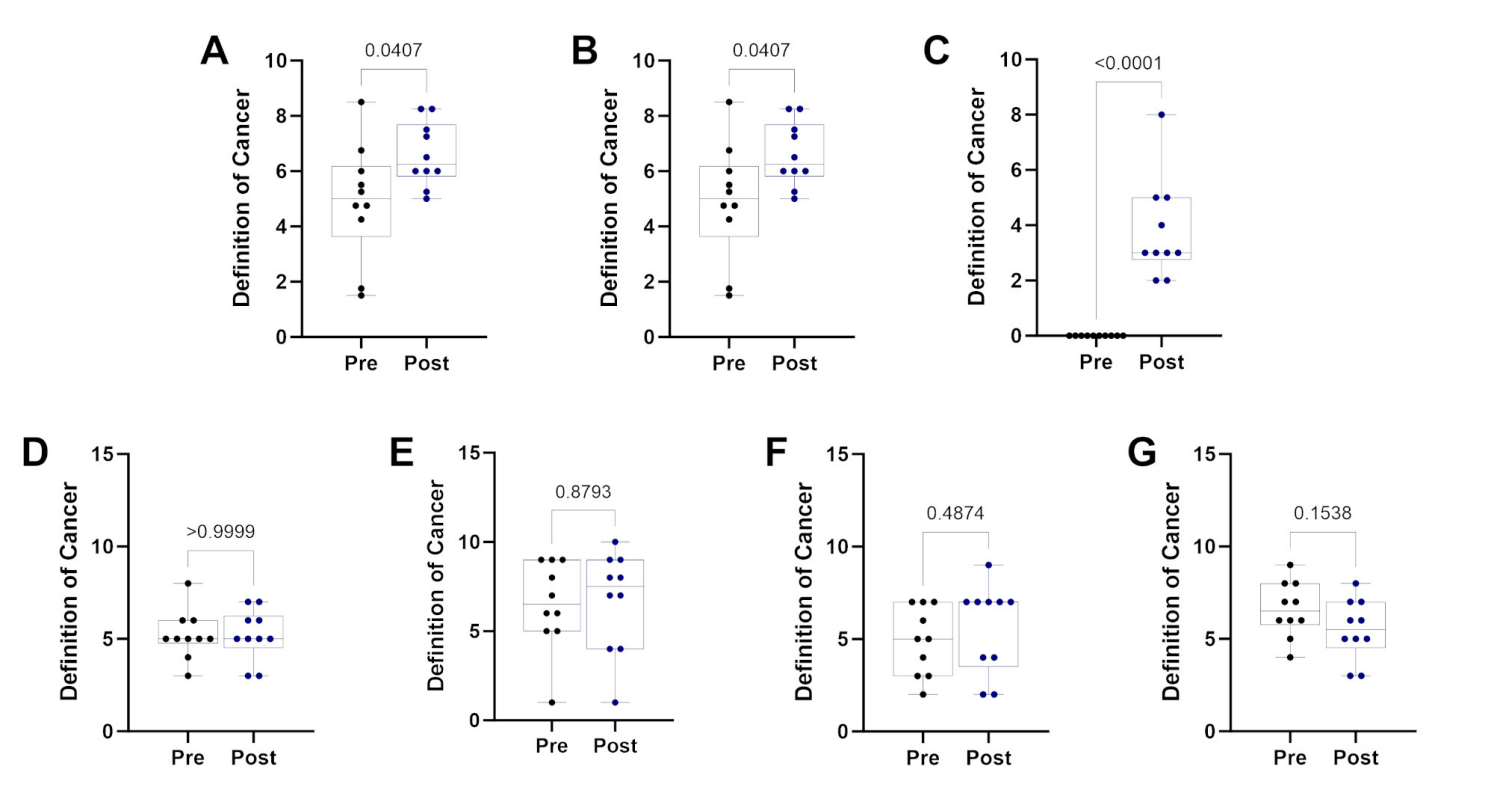
Results of the pre-and post-assessment. (**A**) Definition of cancer: Students’ responses to defining cancer in one sentence, with scores ranging from 1 to 10. (**B**) Cancer therapies: Students’ responses naming cancer therapies. (**C**) Hallmarks of cancer: Students’ responses naming hallmarks of cancer. (**D**) Likelihood of curing cancer: Students’ ratings on a scale of 1 to 10 on the likelihood of scientists curing cancer. (**E**) Likelihood of pursuing research: Students’ ratings on a scale of 1 to 10 on the likelihood of pursuing a research-focused career. (**F**) Likelihood of pursuing cancer research: Students’ ratings on a scale of 1 to 10 on the likelihood of pursuing a career focused on cancer research. (**G**) Perceived difficulty of the course: Students’ ratings on a scale of 1 to 10 on the expected difficulty vs. actual difficulty of the course. A paired parametric Student’s t-test was used to calculate the p-values. Error bars represent standard errors of the mean. The data is assumed to be normally distributed

On the first day of the course, students were evaluated on their baseline knowledge of cancer-related topics and their expectations for the course. The assessment was adopted as previously described [10], lasted 5 min, and included questions on cancer knowledge and hallmarks, treatment options, and their research interest. The students were re-evaluated on their knowledge on the final day of class. Figure 2 presents the distribution of responses for each question.

#### Define cancer in one sentence

The students’ pre- and post-assessment responses were recorded, pooled, and randomized. Instructors then assigned a numerical grade to each response, ranging from 1 to 10. The mean score increased from 4.9 to 6.6 throughout the course. Statistical analysis using a paired student’s t-test revealed a significant difference in scores (*p* = 0.0407).

#### Name as many cancer therapies as you can

Similar recorded therapies were counted only once, and vague answers (e.g., “*drugs*”) were assigned as 0. The mean number of therapies listed increased from 1.7 during the pre-assessment to 3.9 during the post-assessment. General or vague answers were counted once. Statistical analysis using a paired student’s t-test indicated a significant difference in the results (*p* = 0.0040).

#### Name as many hallmarks of cancer as you can

Similar hallmarks were recorded once, and blank responses were assigned a value of 0. Students’ average recollection of cancer hallmarks significantly increased from 0 to 3.8. General or vague answers were counted once. Statistical analysis using a paired student’s t-test revealed a highly significant difference in the results (*p* < 0.0001).

#### How close are scientists to curing cancer?

Students were asked to rate how close scientists were to curing cancer on a scale of 1 to 10 (with 10 indicating high likelihood). The average response scale remained unchanged, with a pre-assessment score of 5.2 and a post-assessment score of 5.2. Statistical analysis using a paired student’s t-test indicated these results were insignificant (*p* > 0.9999).

#### How likely are you to pursue research?

On a scale of 1 to 10 (with 10 indicating high likelihood), students were asked to rate how likely they were to pursue a research-focused career. The average response scale increased, with a pre-assessment score of 6.5 and a post-assessment score of 6.7. However, statistical analysis using a paired student’s t-test indicated that these results were statistically insignificant (*p* > 0.8793).

#### How likely are you to pursue cancer research?

Students were asked to rate, on a scale of 1 to 10 (with 10 indicating high likelihood), how likely they were to pursue a career focused on cancer research. The average response scale increased, with a pre-assessment score of 4.9 and a post-assessment score of 5.6. However, statistical analysis using a paired student’s t-test indicated these results were insignificant (*p* > 0.4874).

#### How difficult do you expect this class to be vs. how difficult was this class?

During the pre-assessment, students were asked to rate, on a scale of 1 to 10 (with 10 indicating high likelihood), how difficult they expected the course to be. During the post-assessment, the students were asked to rate how difficult the course had appeared to them. The average response scale decreased, with a pre-assessment score of 6.6 and a post-assessment score of 5.5. However, statistical analysis using a paired student’s t-test indicated these results were insignificant (*p* > 0.1538).

### Student evaluation of course design

**Fig. 3:**
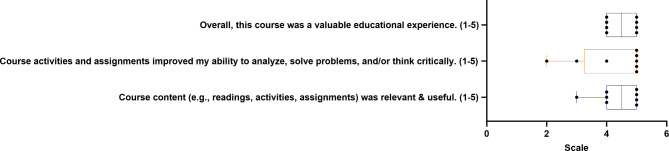
Student evaluation of course design. Student evaluation of course rigor. On a scale of 1 through 5, students were asked to evaluate their educational experience, their improved ability to solve problems and critically think, and the course content. Each point represents a student’s response. Error bars represent standard errors of the mean. The data is assumed to be normally distributed

Students received a post-course evaluation from the SSTP program director, which included rating items on a scale of 1 to 5. These items were: (1) Overall, this course provided a valuable educational experience. (2) Course activities and assignments enhanced my ability to analyze, solve problems, and/or think critically. (3) The course content (e.g., readings, activities, assignments) was relevant and useful. Students’ responses were recorded, as depicted in Fig. 3.

## Discussion

### Revision considerations for cooperative learning

The UF-SSTP program is an immersive experience for high school students interested in research. Our group designed and implemented a 7-week summer course on the fundamentals of cancer biology, focused on cancer hallmarks. Previous instructors of this course reported steady trends in cumulative grade averages over five years and increased basic cancer knowledge [1]. However, this study was published before the COVID-19 pandemic, and therefore, new aspects need to be considered to optimize student learning outcomes post-pandemic. Here, we provided an updated study on teaching methods and student learning outcomes in this summer course on cancer biology after the COVID-19 pandemic.

In 2021, the curriculum underwent revision to accommodate remote learning. We partnered with the UF Center for Precollegiate Education and Training (CPET) pre-scholars program to accommodate virtual learning standards and offer a curriculum that could crosslink Florida learning standards with cancer biology. Since collaborative learning has shown potential improvement in student autotomy [4, 5], we wanted to incorporate this style for remote learning, engaging in peer-to-peer collaboration. Thus, we aimed to maintain course rigor while retaining student autonomy and collaboration. The class convened online for eight sessions, each lasting two hours.

Consequently, the curriculum was adapted with the following considerations: adjusting the course duration, addressing technical constraints associated with the “Zoom” platform, balancing student autonomy with peer collaboration, upholding the academic rigor of the content despite the absence of nonverbal cues from students, and restructuring the contents of cancer hallmarks [11, 12] into manageable, foundational units. To address these considerations, we implemented “lecture workshops,” that is, didactic lectures followed by breakout room case studies to reinforce lecture material. We also implemented follow-along lecture worksheets and frequently asked questions to engage students throughout the lecture.

Based on prior student feedback, we further revised this course in 2022 to broaden the cancer hallmarks and include more clinical applications. We incorporated cancer immunology and focused on incorporating patient data into the student-led workshops. We also provided additional online resource links to support their analysis. For instance, one case study tasked students with identifying two major types of lung cancer and determining which type is most commonly associated with cigarette smoking. Moreover, they were asked to identify the top three most common mutations for each type and classify whether each mutation is a tumor suppressor gene (TSG) or an oncogene. In this case study, we used clinical data provided by The Cancer Genome Atlas (TCGA) to reinforce the concepts of oncogenes and tumor suppressors. As a learning benchmark, students were expected to determine if their “mock” patient has an oncogenic driver or depletion of a tumor suppressor.

In 2022, the cancer hallmarks were revised to include “polymorphic microbiomes” and “non-mutation epigenetic reprogramming” [8]. We further revised the curriculum to incorporate these hallmarks into modules, including cancer immunology from the prior year. This speaks to the versatility of the curriculum style, as we saw steady cumulative grade averages between the 2022 and 2023 cohorts (Fig. 1) despite introducing a more rigorous curriculum. In the following sections, we discuss and interpret the pre-post longitudinal observational study results and dive deeper into each course component. Our main objective is to provide guidelines on how we delivered a course on the fundamentals of cancer biology.

As previously published, student-led learning increased their performance and autonomy [4, 5]. In our study, we observed similar trends in overall students’ performance and autotomy. When students were first introduced to group assignments, a few took on leadership roles, but not many team-based decisions were made among group members. Throughout the course, we noticed that more students participated in group discussions and activities as a team, fostering collaboration, negotiation, and problem-solving skills. During the lectures, we observed an improvement in the number of questions asked. Additionally, the questions they asked demonstrated an improvement in critical thinking regarding the course lectures. We noted that students would apply principles acquired during previous lectures in their questions, indicating improvement in their critical thinking.

### Evaluation of student knowledge comprehension

The final grade averages served as a comparative assessment between years. We included 2017 and 2018 as a reference point for the curriculum prior to modifications in 2021. In 2017 and 2018, the course was taught in person by previous instructors [1]. In 2021, the course was taught virtually amid limitations in place via COVID-19. In 2022 and 2023, the course was taught in person following the removal of COVID-19 restrictions. We cannot account for the differences in educational backgrounds among cohorts. However, students are generally selected from similar regions each year. Moreover, we cannot account for the differences in how prior course instructors implemented the curriculum. We altered the curriculum in 2021 to comply with remote learning restrictions. When the restrictions were lifted, we adopted the same approach for future cohort installments (2022 and 2023), and we observed steady rigor in the grades tracked. We also observed improvement in the minimum grade average in 2022 and 2023 (Table 3). It is important to note that while we do not want to compare between cohorts, we do want to emphasize that the changes we made throughout 2021–2023 did not significantly worsen student performance. We also must note that the improvements we implemented may have altered final performances throughout the years, and we observed that some cohorts had closely distributed cumulative grades while others did not. This may be due to differences in foundational knowledge and variations in instructors’ delivery of the material. We believe adding follow-along assignments and student-led cooperative group assignments improved students’ autonomy and comfort with the subject area.

When we asked students to define cancer in one sentence, we observed a significant increase in the quality of the responses in the post-course assessment compared to the pre-assessment (Fig. 2A). For instance, general responses submitted for the pre-assessment included “*a foreign body that can spread throughout the body*” and “*cell mutation in the human body*.” In contrast, in the post-assessment, we observed higher-quality responses such as “*a mutation in a cell that leads to the uncontrolled dividing and spreading of malignant cells.*” We also noticed that some students incorporated cancer hallmarks and terms (“*defect of cells resulting in oncogenes that multiply quickly*,* spread throughout the body*,* and cause tumors*”) in the general responses after the course, which indicated that students retained general information throughout the course. Moreover, students also demonstrated the ability to connect key concepts over the duration of the course. For example, some students recognized the link between genomic instability and the potential for cancer-associated antigen presentation, recognizing avenues for targeted therapy or immunotherapy.

We next asked students to name as many cancer treatments as they could (Fig. 2B). During the pre-assessment, a lot of the answers were traditional therapies such as “*radiation*,” “*chemotherapy*,” and “*surgery.*” In the post-assessment, the variety of responses significantly improved to include both standard therapies as well as more precision-based therapies such as “*immunotherapies (CAR T cells)*,” “*gene therapy*,” “*hormone therapy*,” “*fecal microbiota transplant (FMT)*,” and “*bone marrow transplants*,” among others. Our focus throughout the course was to increase exposure to targeted therapies and novel therapies not commonly discussed, for instance, “*adoptive cell therapy*” and “*small tyrosine-kinase inhibitors*.” The interactive group learning assignments covered novel therapeutic approaches, likely aiding students’ high retention of the course material.

The most remarkable findings emerged when students were asked to identify as many cancer hallmarks as they could (Fig. 2C). Initially, during the pre-assessment, responses ranged from “*What is a hallmark?*” and “*not sure*” to some leaving the question unanswered. However, following the course, there was a statistically significant increase in the quantity and quality of the responses, exceeding our expectations. For instance, one student provided a summary list including “*sustaining proliferative growth*,* metastasis*,* tumor-promoting inflammation*,* developing immune escape*,* senescent cells*,* dysregulated cell metabolism*,* resisting cell death*,* genome mutations*,” and another answered “*angiogenesis*,* cell proliferation*,* evading cell death*,* polymorphic biomes*,* irregular cellular regulation.*” Notably, the students correctly identified cancer hallmarks within the allotted time.

Another notable trend between pre-assessment responses and post-assessment responses is the likelihood of pursuing research (Fig. 2D), specifically cancer research (Fig. 2E). Although no statistical significance could be observed throughout the course, there was a noticeable upward trend in median responses after the course ended. Furthermore, when assessing the course difficulty responses, the data indicate that some students did not feel like the course was as challenging as initially thought (Fig. 2G). However, the responses from that survey were non-statistically different.

The SSTP program director also gathered feedback from students regarding the course difficulty and design (Fig. 3). Students were asked to rate three prompts on a scale of 1–5, where 1 indicated “*strongly disagree*” and 5 indicated “*strongly agree*.” To the first prompt (overall, this course was a valuable educational experience), 50% of students answered 4, and the other 50% gave a score of 5, indicating the students found the course to be of educational value. This was coupled with additional feedback, with one student stating: “*The case studies were a lot of fun and provoked critical thinking in a way I’ve never experienced*”.

The second prompt interrogated if the course activities and assignments improved the students’ ability to analyze, solve problems, and/or think critically. While most students (62.5%) gave a rating of 5, one student gave a response of 2, another gave a score of 3, and the other gave a score of 4. Additional feedback highlighted the effectiveness of educational activities such as escape rooms and case studies, with responses like: “*The educational activities we did at the end of class (e.g.*,* escape room*,* case studies)*” and “*Learning about cancer and getting to work in groups and play games*” further reinforcing the curriculum style. The final prompt asked whether the course content (e.g., readings, activities, assignments) was relevant and valuable. Once again, most students responded with a rating of 5 or 4 (50% or 37.5, respectively), with only one response rating of 3. Overall, we felt confident that these students benefited from the course and enhanced their learner autonomy.

Students were also encouraged to provide additional comments, and only one did so. The response mentioned, “*The terminology was difficult to fully understand*,* and the lessons felt a little rushed each time. Adding more assignments would help us understand the information more and apply it*.” Although the course is condensed into 22 contact hours, future curriculum revisions may consider providing optional pre-reading assignments to help students grasp more challenging concepts.

### Final assessment

Due to the limited time and course lecture design, we could not proctor the final assessment. However, to ensure academic honesty, we added safeguards to the evaluation. We added a time limit of 95 min, randomized the assessment questions, and locked submitted assessments until the instructors graded all assessments. These steps would allow students enough time for multiple-choice and short-answer essay questions while discouraging academic dishonesty. We also noted how long it took for each student to complete the assessment and the time and date of completion. There is no indication of academic dishonesty for the final examination (data not shown).

### Lessons learned and applications

Some of the essential skills required for a successful career in STEM include the ability to effectively communicate, lead, think critically, collaborate, and summarize and interpret scientific literature and data. Throughout the course, these students participated in several peer-to-peer activities that prompted them to independently improve all of these skills. We observed improvements in student-led leadership and the enhancement of shared ideas when students engaged after didactic lectures. Moreover, the students were more resourceful, taking the initiative to look up and understand the case studies and assignments during that time.

Students also presented case studies and self-coordinated with each other to determine sections to present. This case study presentation was timed, and students effectively communicated a lot of information on a topic they had not previously known. During the escape room, we observed peak performance in student collaboration, with students working together to connect main ideas across lectures to decode the escape room.

Additionally, students participated in a career overview panel. The panelists were from several fields in STEM and are described in the extended methods (Attachment 5). During this time, students were prompted to develop several questions to ask the panelists. Most of the students were unfamiliar with all aspects of STEM, especially careers outside of medicine in cancer, and found the panel informative. Other studies have addressed that students generally have limited knowledge of STEM careers and that early exposure can enhance their interest in pursuing other STEM-related careers [13, 14]. Our study showed a non-statistical mean increase in students likely to pursue research, specifically cancer research. Altogether, students not only improved their general knowledge but demonstrated improvement in all the skills required to navigate future careers in STEM. This is corroborated by prior literature that has shown collaboration improves these skills [4, 5].

### Limitations of this study

One important caveat is that we are not comparing online virtual learning from the CPET pre-scholars program in 2021 to the in-person modified course in 2023. The conditions and requirements for student selection were changed in 2021. We also tracked student grades across five years to assess if our modifications in 2021 disparaged grading standards when students were allowed to return to in-person learning. Therefore, the grades tracked over time are used to ensure grading standards. This study includes grades collected prior to 2021 by different course instructors who were responsible for the course. Therefore, inconsistencies in the final evaluation between 2017 and 2022 may exist. The Course feedback and cumulative grades from 2021 to 2022 have been collected, dating before and during course modification. However, cumulative grade averages were not presented, limiting the comprehensive understanding of student performance. Furthermore, variations in teaching styles due to changes in course instructors could have influenced the results, suggesting the need for consistency in instructional approaches for future studies. We note that the final examination was conducted unsupervised, and we have added measures to reduce academic dishonesty, but these may exist, skewing the data. Therefore, we placed emphasis on lessons learned from our experience implementing this cancer biology and therapeutic course. Lastly, our pre-post observational study was conducted for one cohort. Future instructors should track responses for long-term and consistent results.

## Conclusion

Overall, our course aimed not only to teach students about different concepts of cancer development and hallmarks but also to encourage students to explore different careers within the STEM field. In our experience, we have found that students at the high school stage have not been exposed to a wide range of STEM careers, leading to many students focusing on a career in medicine as their only option. In this course, we aimed to highlight alternative STEM careers to medicine to help students make informed decisions on their future careers.

In summary, we imparted a summer course on cancer hallmarks, adapting our teaching strategy to the post-COVID-19 era and measuring student learning outcomes. The results indicated that students performed better in the post-course assessment than in the initial evaluation, indicating that students could successfully understand and retain concepts related to diverse cancer hallmarks. Students also demonstrated improved critical thinking and enhanced collaborative and communication skills. We hope our detailed overview of teaching methodologies and outcome assessment approaches can assist fellow instructors in crafting courses in the post-COVID-19 pandemic era. This curriculum can be adapted based on the level and the number of students per class, and it can be modified to cover additional cancer hallmarks.

## Electronic supplementary material

Below is the link to the electronic supplementary material.

Supplementary Material 1

Supplementary Material 2

Supplementary Material 3

Supplementary Material 4

Supplementary Material 5

## Data Availability

The data supporting the findings of this study are available upon reasonable request from the University of Florida Cancer Center.
