# Supplementary material for: A summer course in cancer for high school students-an update on lessons taught and lessons learned
Source: BMC Med Educ. 2024 Sep 17;24:1020. doi: 10.1186/s12909-024-06002-z (PMC11409685; doi:10.1186/s12909-024-06002-z)
Supplement: Supplementary file 1 — Supplementary Material 1 [file 12909_2024_6002_MOESM1_ESM.docx]

**Escape Room Review: Learning Objectives**

Understand mechanisms involved in Avoiding Immune Destruction, including:

- Tumor Promoting Inflammation
- Immune Desert
- Immune Exclusion

Explore strategies for Evading Growth Suppressors, covering:

- Cellular Responses
- DNA Damage
- Cell Proliferation
- Cell Survival

Examine the role of Genomic Instability & Mutation, focusing on:

- Oncogenic Activation
- Neoantigen Presentation
- Cancer Transformation

Analyze pathways involved in Sustaining Proliferative Signaling, such as:

- Oncogenic Signaling
- Damaging Mutations
- Bypassing Growth Factor Activation

Investigate the impact of the Polymorphic Microbiome, including:

- Therapeutic Index
- Healthy Gut Microbiome
- Fecal Microbiota Transplantation (FMT)

Understand mechanisms associated with Adaptive and Innate Immune Responses in Avoiding Immune Destruction.

Explore Nonmutational Epigenetic Reprogramming, covering:

- Methylation
- DNMT3A
- TET
- Epigenetic Gene Regulation
