# Supplementary material for: A summer course in cancer for high school students-an update on lessons taught and lessons learned
Source: BMC Med Educ. 2024 Sep 17;24:1020. doi: 10.1186/s12909-024-06002-z (PMC11409685; doi:10.1186/s12909-024-06002-z)
Supplement: Supplementary file 2 — Supplementary Material 2 [file 12909_2024_6002_MOESM2_ESM.docx]

**Decoding Cancer: A Multidimensional Challenge**

*Part 1: Case Study - Unraveling the Mysteries*

Read the following case study and answer the questions based on your understanding of cancer hallmarks, basic research, translational research, and drug discovery.

Case Study:

A research team is investigating a rare and aggressive type of cancer. They have identified specific genetic mutations in tumor cells that contribute to the initiation and progression of this cancer. Your task is to analyze the given information and answer the questions below.

1. List and describe three cancer hallmarks that are likely to be associated with the identified genetic mutations in this aggressive cancer.
2. Explain how these genetic mutations can lead to the development of these cancer hallmarks.

*Part 2: Basic Research - Unveiling the Mechanisms*

Now, let's dive deeper into the basic research aspect. Research the provided questions to uncover the mechanisms associated with the identified genetic mutations.

1. Identify and list the specific genetic mutations commonly observed in this type of cancer and describe their impact on cellular processes and pathways involved in cancer development.
2. Explore and describe the molecular mechanisms through which these genetic mutations contribute to the acquisition of the identified cancer hallmarks.

*Part 3: Translational Research - Bridging the Gap*

Shifting our focus to translational research, uncover the potential therapeutic strategies for targeting the identified genetic mutations.

1. Identify two potential targeted therapies or molecularly targeted agents that can specifically inhibit the effects of the identified genetic mutations. Research their names and mechanism of action.
2. Explain how each targeted therapy or molecularly targeted agent can interfere with the molecular pathways affected by the genetic mutations, leading to the reversal or suppression of the identified cancer hallmarks.

*Part 4: Drug Discovery - Cracking the Code*

In the final stage, explore the drug discovery process and investigate potential challenges and strategies related to the development of targeted therapies.

1. Research and identify the current challenges faced in developing targeted therapies for the identified genetic mutations, including issues related to drug efficacy, resistance, or toxicity.
2. Suggest a potential strategy or approach to overcome the challenges identified in question 7 and enhance the effectiveness and safety of the targeted therapies.

The Final Question:

Based on the provided information, propose a hypothetical combination therapy that incorporates the two targeted therapies or molecularly targeted agents identified in part 3. Explain how this combination therapy could synergistically target multiple cancer hallmarks associated with the identified genetic mutations.
