# Supplementary material for: A summer course in cancer for high school students-an update on lessons taught and lessons learned
Source: BMC Med Educ. 2024 Sep 17;24:1020. doi: 10.1186/s12909-024-06002-z (PMC11409685; doi:10.1186/s12909-024-06002-z)
Supplement: Supplementary file 3 — Supplementary Material 3 [file 12909_2024_6002_MOESM3_ESM.docx]

**Final assessment questions**

**Central Dogma and Cancer Hallmarks section**

1. Which of the following statements accurately represents the Central Dogma of molecular biology?
   1. DNA is transcribed into RNA, and RNA is translated into proteins.
   2. RNA is transcribed into DNA, and DNA is translated into proteins.
   3. RNA is translated into DNA, and DNA is transcribed into proteins.
   4. DNA is translated into RNA, and RNA is transcribed into proteins.
2. Which of the following accurately describes the relationship between the central dogma of molecular biology and cancer hallmarks?
   1. Mutations in the central dogma machinery directly cause cancer development.
   2. The central dogma plays a role in controlling cell proliferation, which is a hallmark of cancer.
   3. The central dogma is a set of hallmark characteristics observed specifically in cancer cells.
   4. The central dogma only applies to normal cells and is not relevant in understanding cancer biology.
3. Which of the following scenarios related to the central dogma and gene regulation is associated with the hallmark of "sustaining proliferative signaling" in cancer?
   1. Overexpression of microRNAs that inhibit mRNA degradation.
   2. Mutations in DNA repair genes leading to genomic instability.
   3. Enhanced activity of DNA methyltransferases leading to gene silencing.
   4. Mutation in a proto-oncogene leading to constitutive activation of its protein product.
4. Name one of the recognized hallmarks of cancer and briefly explain its significance in cancer development.
5. Describe how mutations in the epidermal growth factor receptor (EGFR) gene contribute to cancer development, and which cancer hallmarks are influenced by these mutations?
   1. Constitutive activation of the EGFR receptor, resulting in uncontrolled and continuous signaling even in the absence of growth factors. Sustaining proliferative signaling, evading growth suppressors, resisting cell death, promoting angiogenesis, and enhancing metastasis

**Oncogenes, tumor suppressors, and epigenetics and cancer section**

1. Which statement about epignetics is incorrect?
   1. Epigenetics is the study of heritable changes that do not affect the DNA sequence
   2. Epigenetics is the study of how cells control gene activity without changing DNA sequence
   3. Epigenetic changes are modifications to the DNA (ex. methylation) that regulate whether genes are turned on or off.
   4. Epigenetics includes the study of changes that do not change the DNA sequence, as well as mutations that permanently change the DNA sequence.
2. DNA hypomethylation is defined as:
   1. Too much DNA methylation
   2. Too little DNA methylation
   3. Just the right amount of DNA methylation
   4. None of the above
3. What type of mutation could lead to oncogene activation?
   1. Chromosomal translocation
   2. Chromosomal duplication
   3. Missense mutation
   4. All of the above
4. Briefly describe the process of DNA methylation (hint: what two enzymes are involved?). Describe how much methylation you would expect to find at a tumor suppressor and an oncogene in cancer and why.
5. Normal cells have a very controlled process of growing and dividing. In cancer cells, talk about the role of oncogenes and tumor suppressors, and give one example of a specific oncogene and a tumor suppressor. Bonus points if you can name all 4 normal functions of the tumor suppressor we talked about in class.

**Cancer Immunology section**

1. Select which of these statements about the tumor microenvironment (TME) is false:
   1. The tumor microenvironment is the ecosystem that surrounds a tumor inside the body
   2. The tumor microenvironment is composed of tumor cells only
   3. A tumor and its microenvironment constantly interact and influence each other, either positively or negatively
   4. Understanding the tumor microenvironment may help improve cancer treatment
2. What is correct about immunotherapy?
   1. Immunotherapy is the immune system
   2. Immunotherapy is a type of treatment that helps the immune system to better act against cancer.
   3. Immunotherapy uses the body’s immune cells to initiate an immune response to eliminate cancer
   4. AB
   5. BC
   6. All of the above
3. Which of these are lines of defense in the body?
   1. Anatomic barriers
   2. Complement
   3. Adaptive Immunity
   4. Innate immunity
   5. ABC
   6. ABD
   7. All of the above
4. Briefly state 3 main differences between the innate and adaptive immune systems and describe the phases of an immune response (Hint: which immune response happens first, and last…). Bonus: in one or two sentences, how do vaccines work and why are they important against certain diseases?
5. Describe the cancer immunity cycle. If a patient presents with a hot tumor (lots of neoantigens and mutations) such as melanoma, what do you think would be a good treatment option for this patient?

**Microbiome in Cancer section**

1. DNA damage is caused by:
   1. *Escherichia. coli* pks+
   2. Lab grown *Escherichia coli*
   3. All of the above
   4. None of the above
2. Short chain fatty acids are:
   1. Acetate
   2. Butyrate
   3. Propionate
   4. and c) only
3. Name the bacteria increased in a Western diet:
   1. Prevotella
   2. Bifidobacteria
   3. a) and b) only
   4. Firmicutes
4. Describe the process of Fecal Microbiota Transplantation (Essay type Question)
5. Explain therapeutic index. (Short answer)
