# Supplementary material for: A summer course in cancer for high school students-an update on lessons taught and lessons learned
Source: BMC Med Educ. 2024 Sep 17;24:1020. doi: 10.1186/s12909-024-06002-z (PMC11409685; doi:10.1186/s12909-024-06002-z)
Supplement: Supplementary file 4 — Supplementary Material 4 [file 12909_2024_6002_MOESM4_ESM.pptx]

## Slide 1
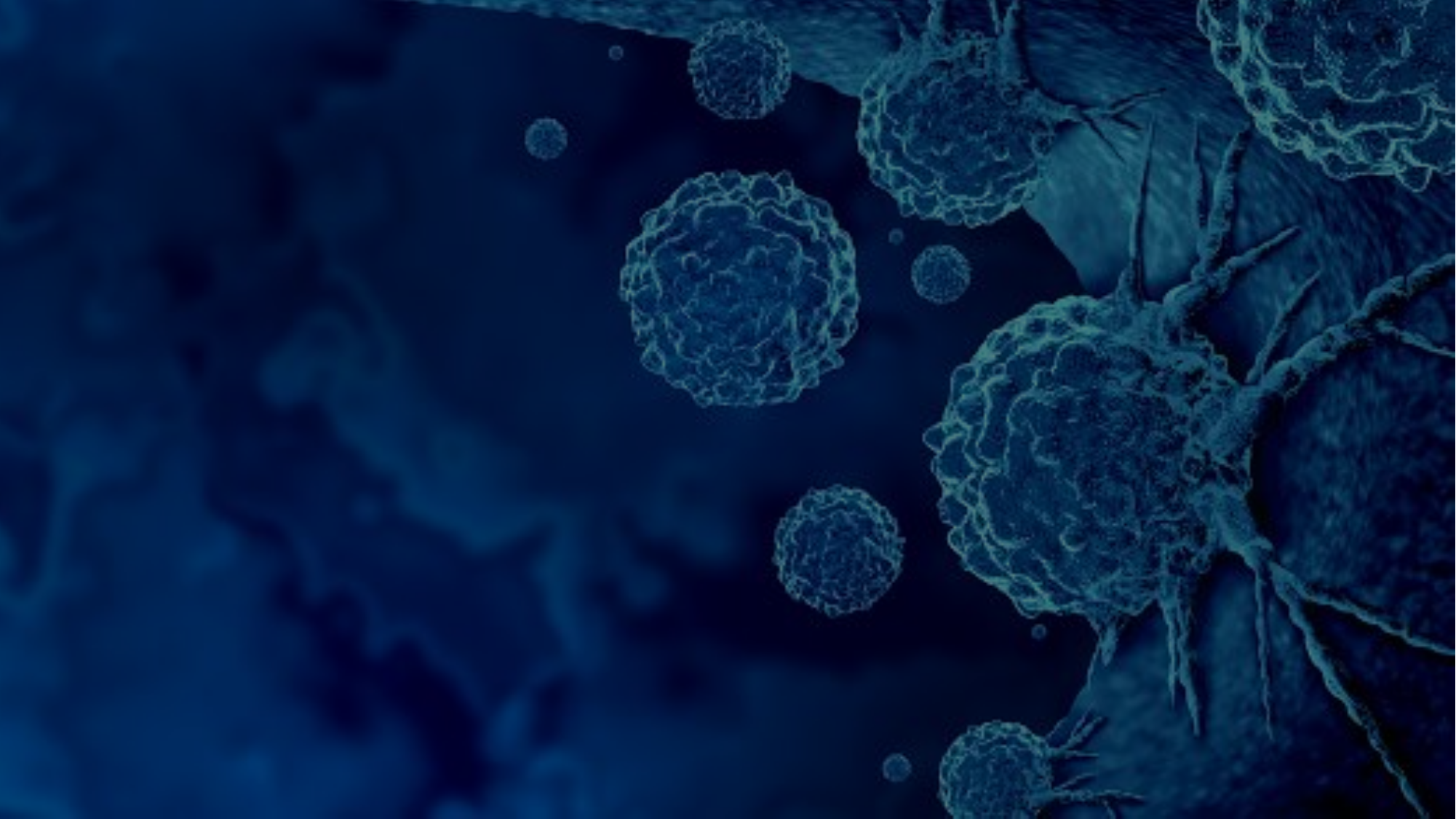

Game Play
Introduction
More
CANCER HALLMARK

## Slide 2
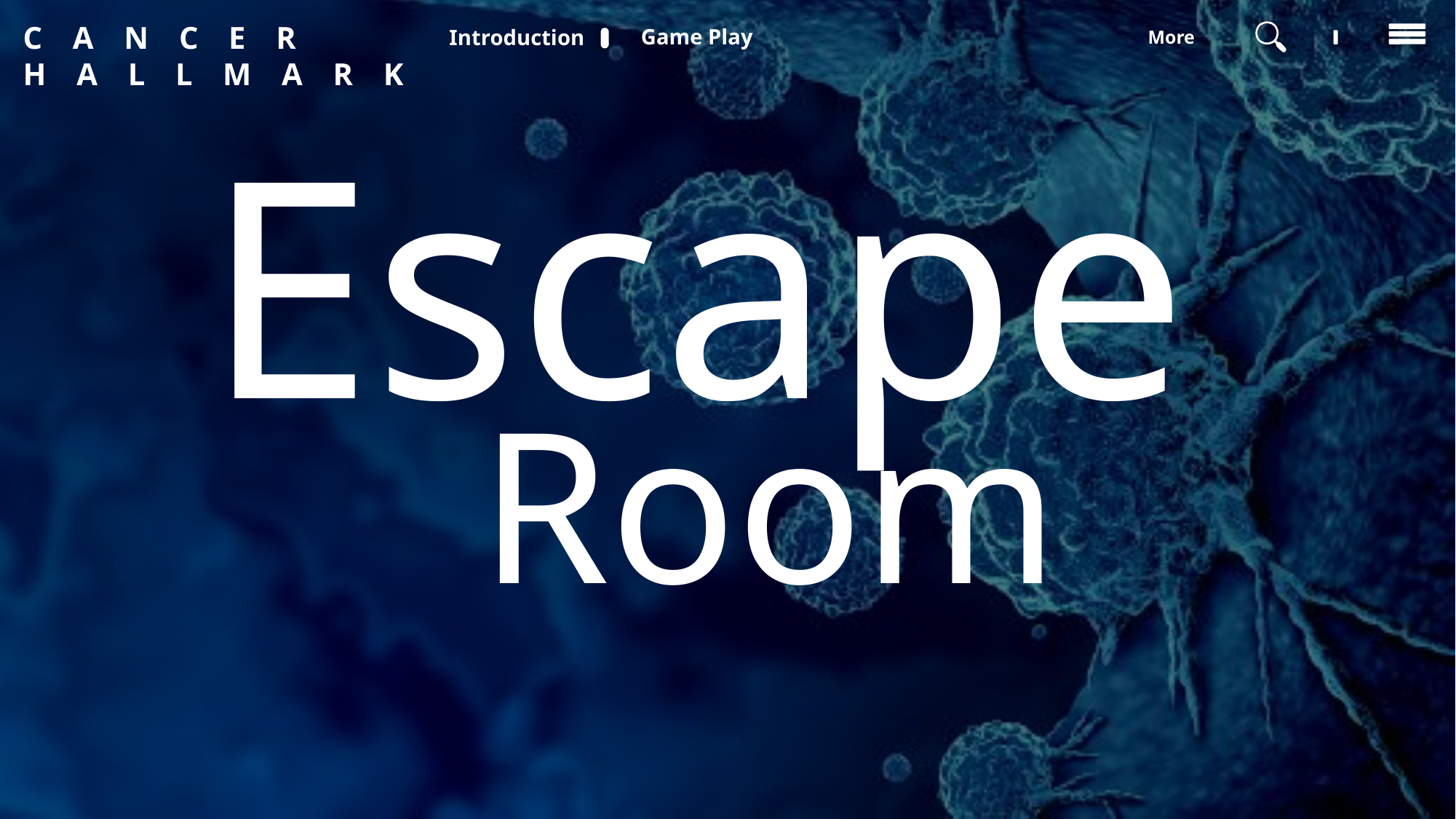

CANCER HALLMARK
Game Play
Introduction
More
Escape
Room
Empire State Building
1,250 feet
Empire State Building, steel-framed skyscraper rising 102 stories that was completed in New York City in 1931 and was the tallest building in the world until 1971. The Empire State Building is located in Midtown Manhattan, on Fifth Avenue at 34th Street.

## Slide 3
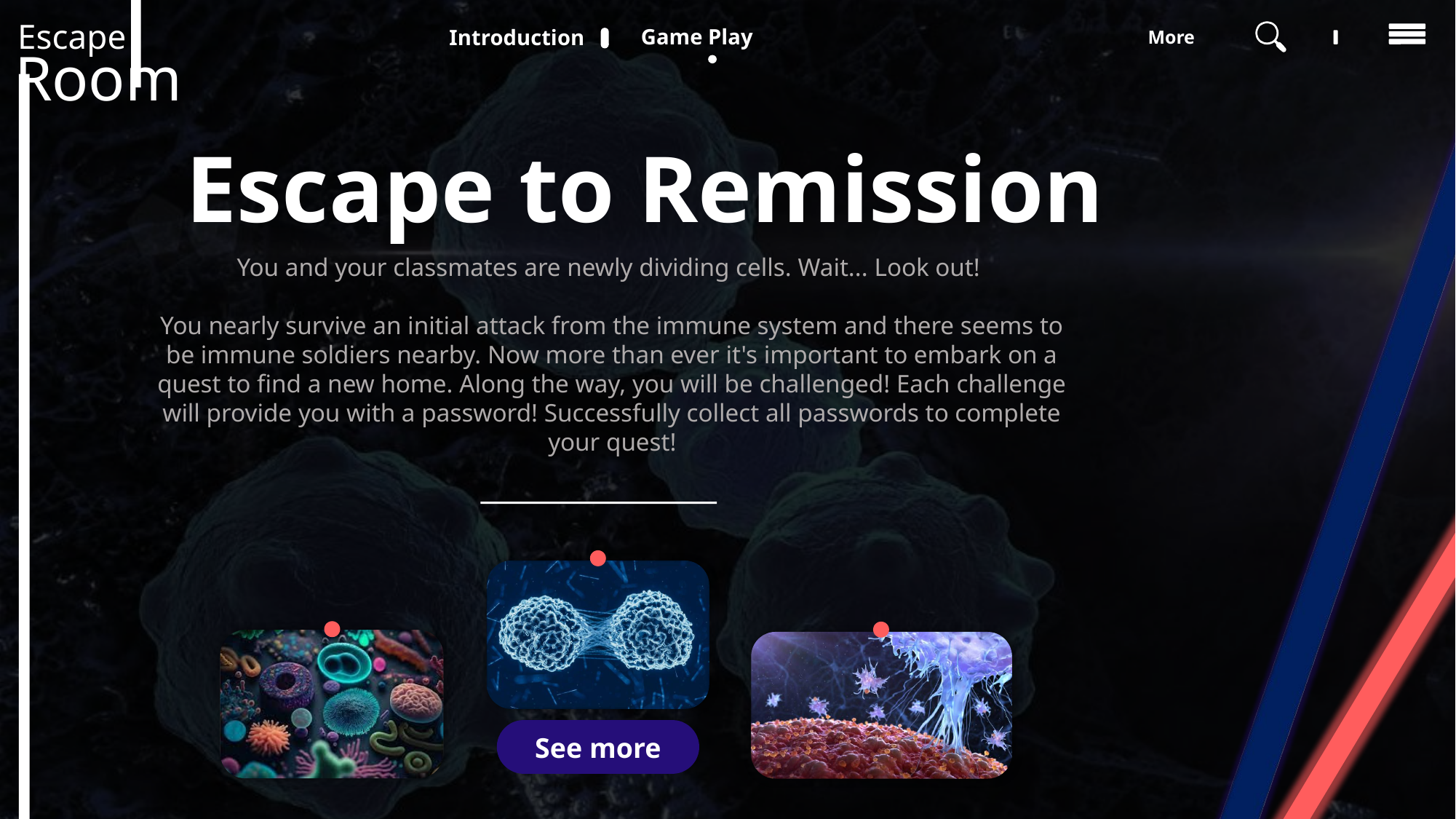

Escape
Game Play
Introduction
More
Room
Escape to Remission
You and your classmates are newly dividing cells. Wait... Look out!
You nearly survive an initial attack from the immune system and there seems to be immune soldiers nearby. Now more than ever it's important to embark on a quest to find a new home. Along the way, you will be challenged! Each challenge will provide you with a password! Successfully collect all passwords to complete your quest!
See more

## Slide 4
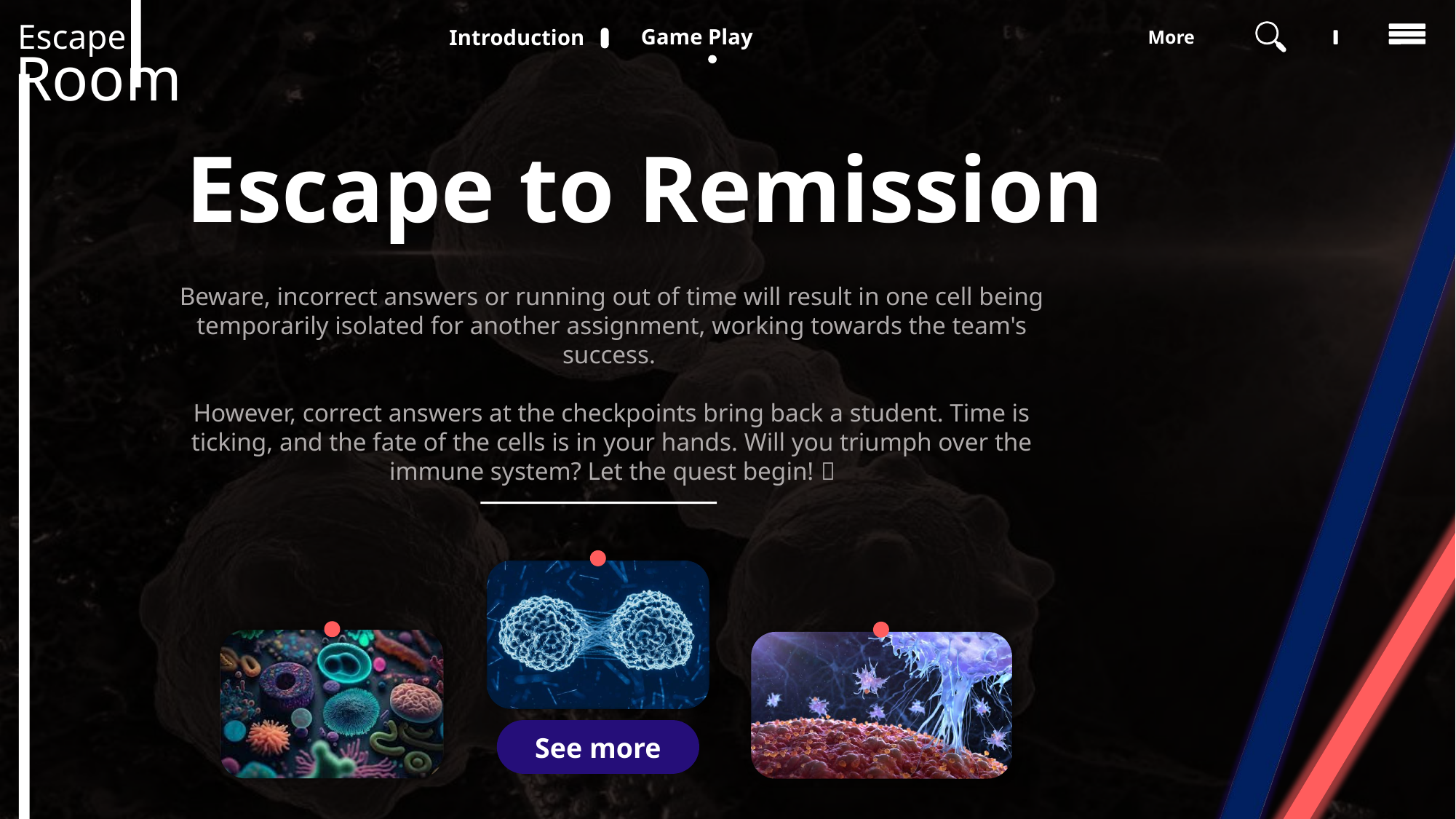

Escape
Game Play
Introduction
More
Room
Escape to Remission
Beware, incorrect answers or running out of time will result in one cell being temporarily isolated for another assignment, working towards the team's success.
However, correct answers at the checkpoints bring back a student. Time is ticking, and the fate of the cells is in your hands. Will you triumph over the immune system? Let the quest begin! 
See more

## Slide 5
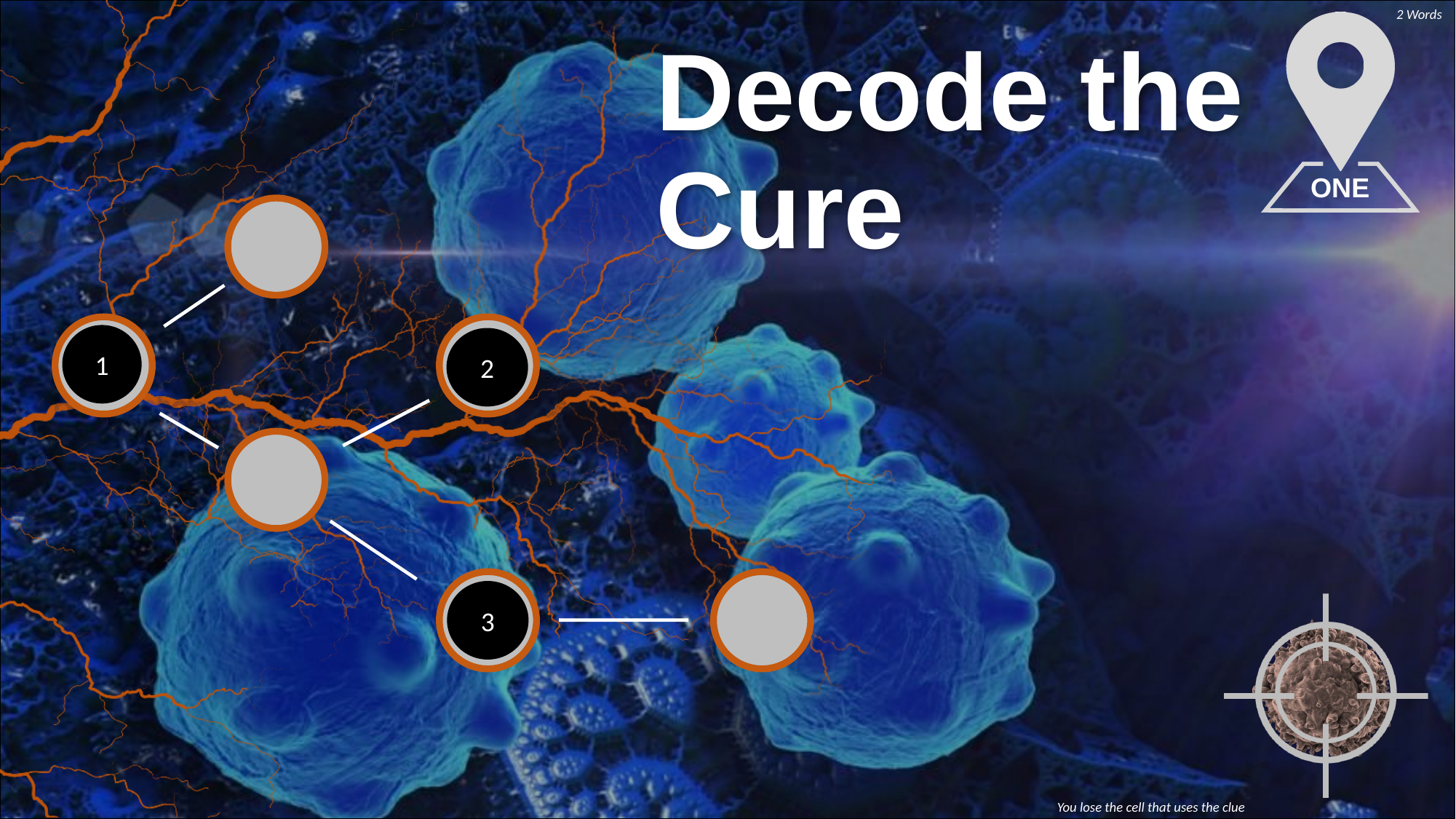

2 Words
Decode the Cure
ONE
1
2
3
You lose the cell that uses the clue

## Slide 6
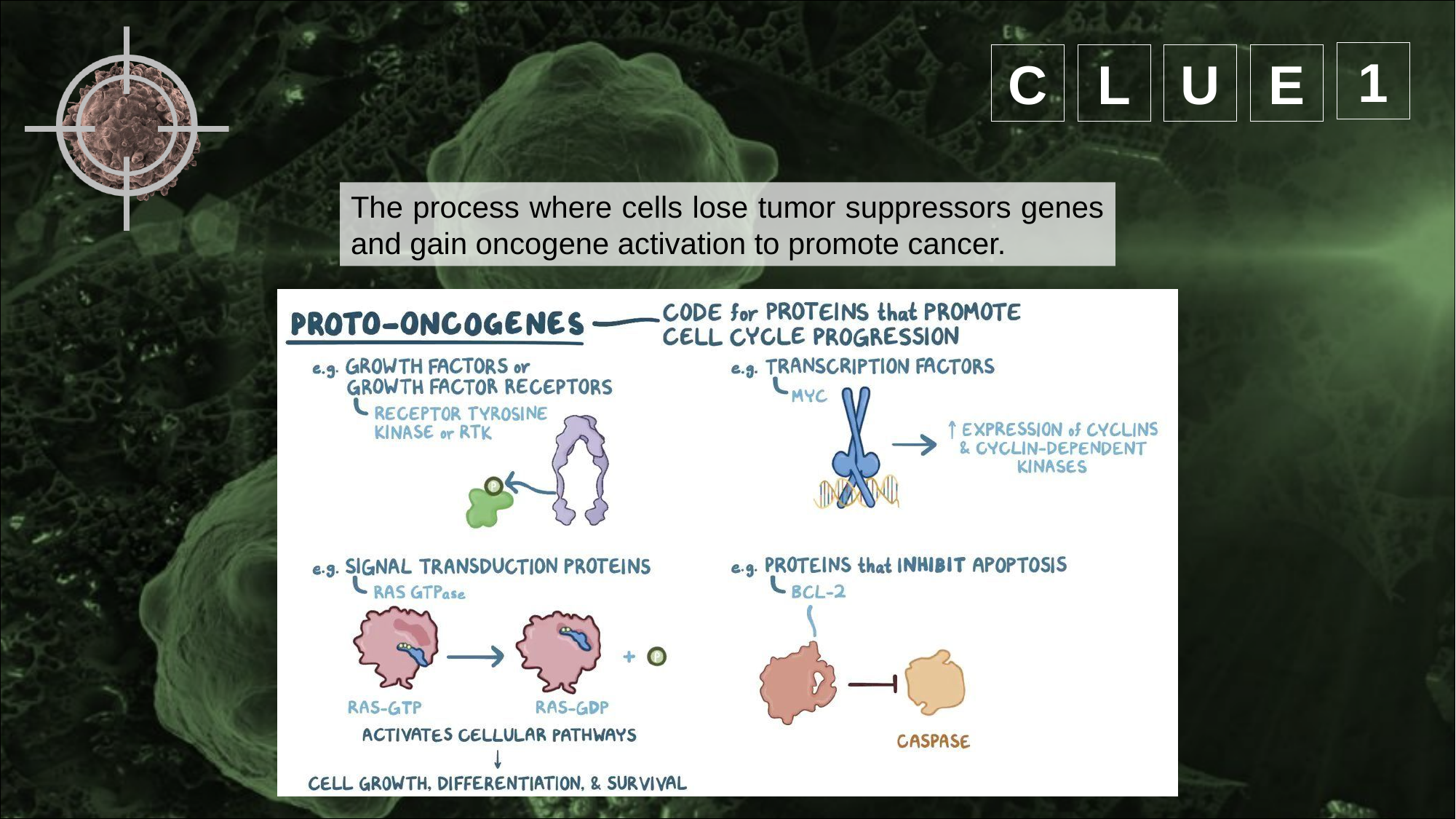

1
C
L
U
E
The process where cells lose tumor suppressors genes and gain oncogene activation to promote cancer.

## Slide 7
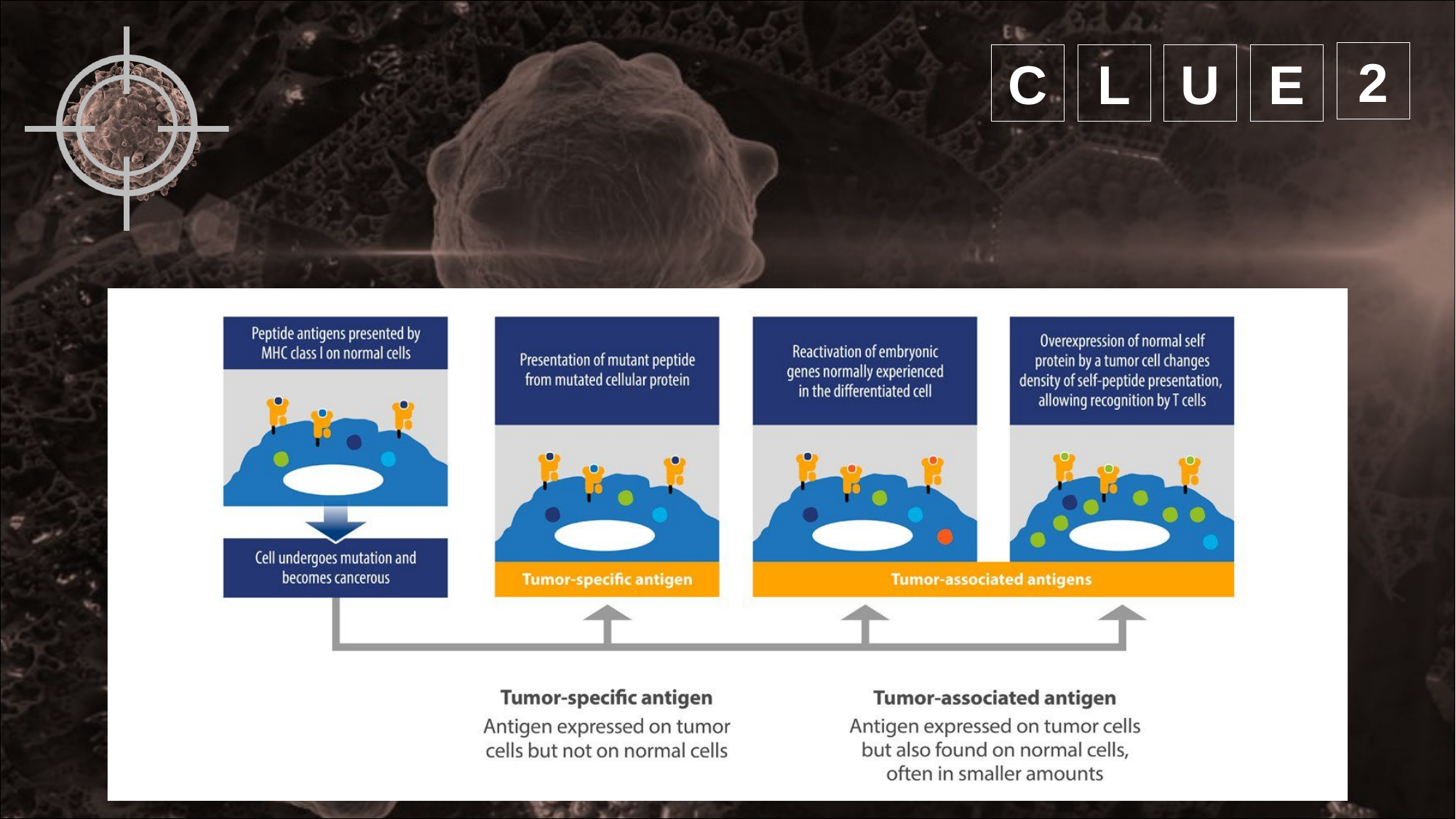

2
C
L
U
E

## Slide 8
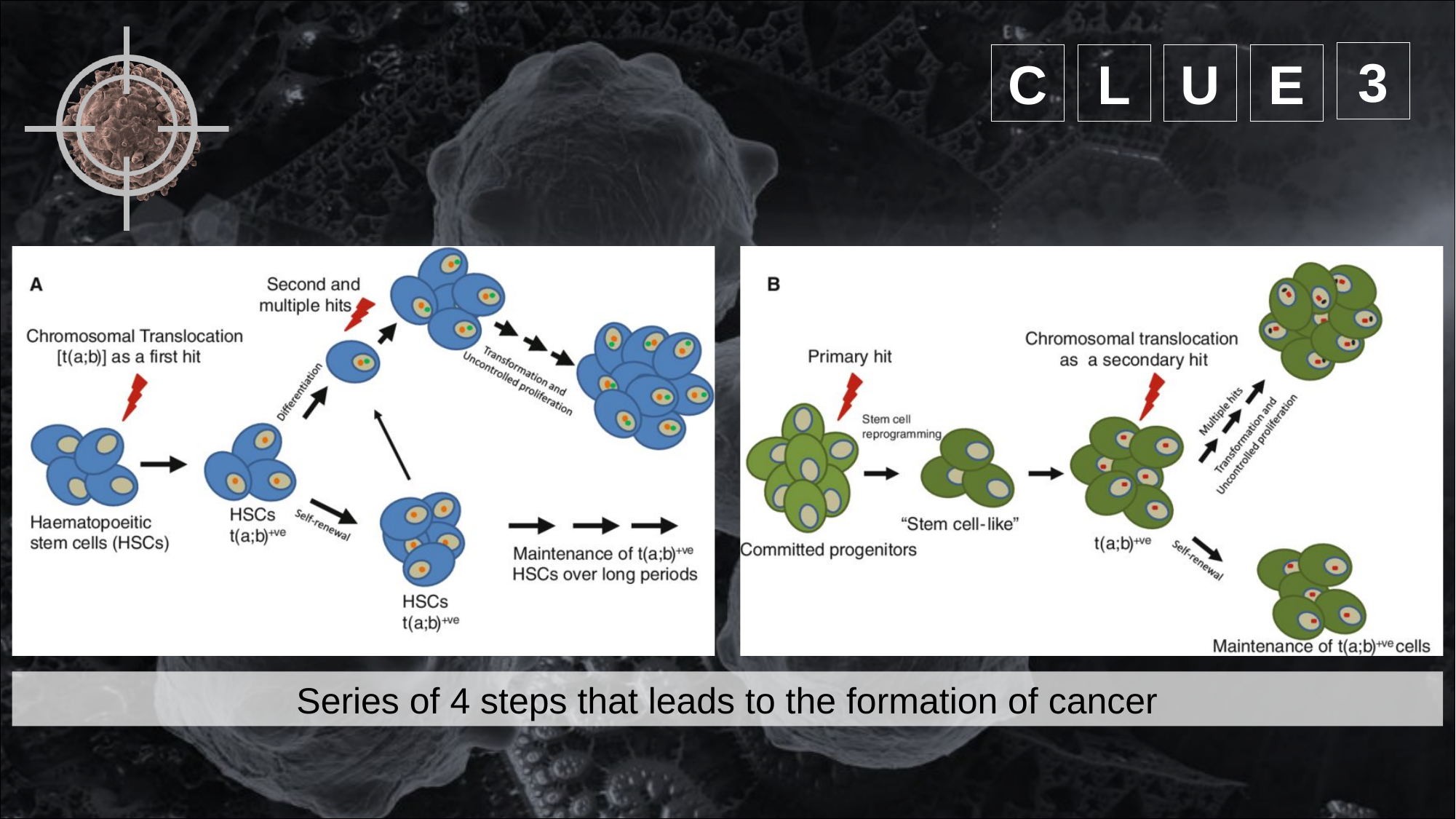

3
C
L
U
E
Series of 4 steps that leads to the formation of cancer

## Slide 9
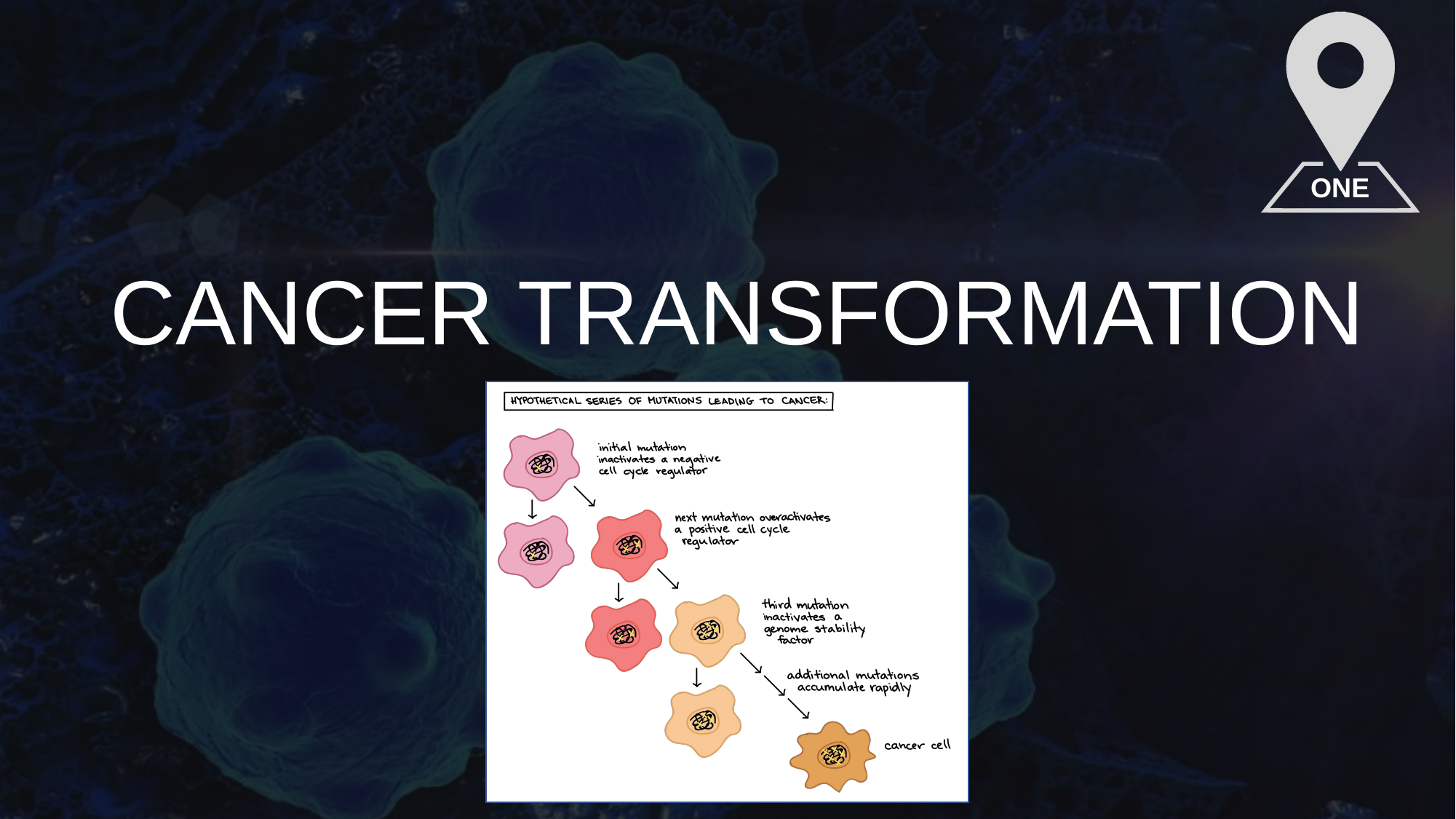

ONE
Cancer TRANSFORMATION

## Slide 10
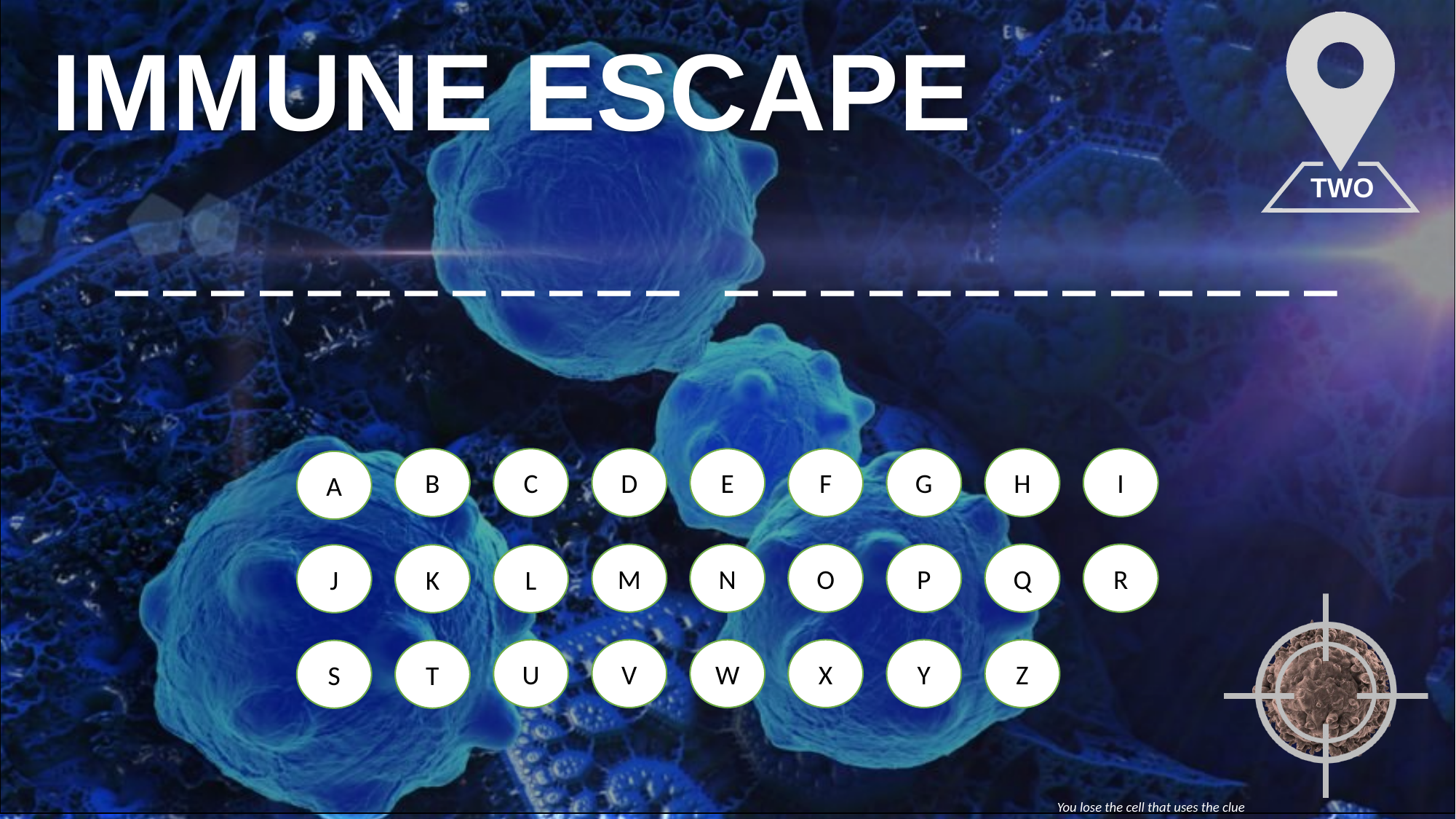

Immune Escape
TWO
_ _ _ _ _ _ _ _ _ _ _ _ _ _ _ _ _ _ _ _ _ _ _ _ _
B
C
D
E
F
G
H
I
A
M
N
O
P
Q
R
J
K
L
U
V
W
X
Y
Z
S
T
You lose the cell that uses the clue

## Slide 11
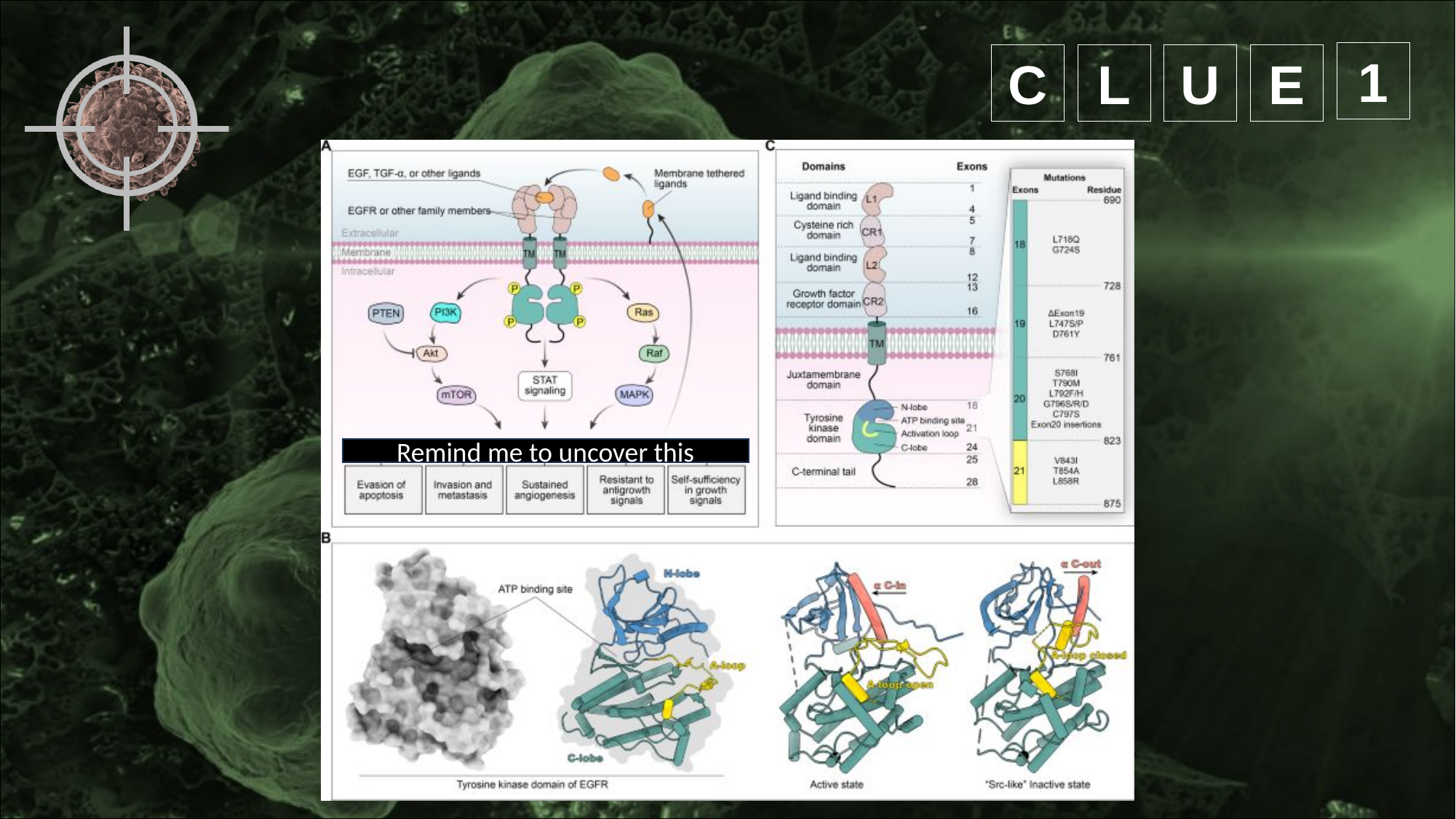

1
C
L
U
E
Remind me to uncover this

## Slide 12
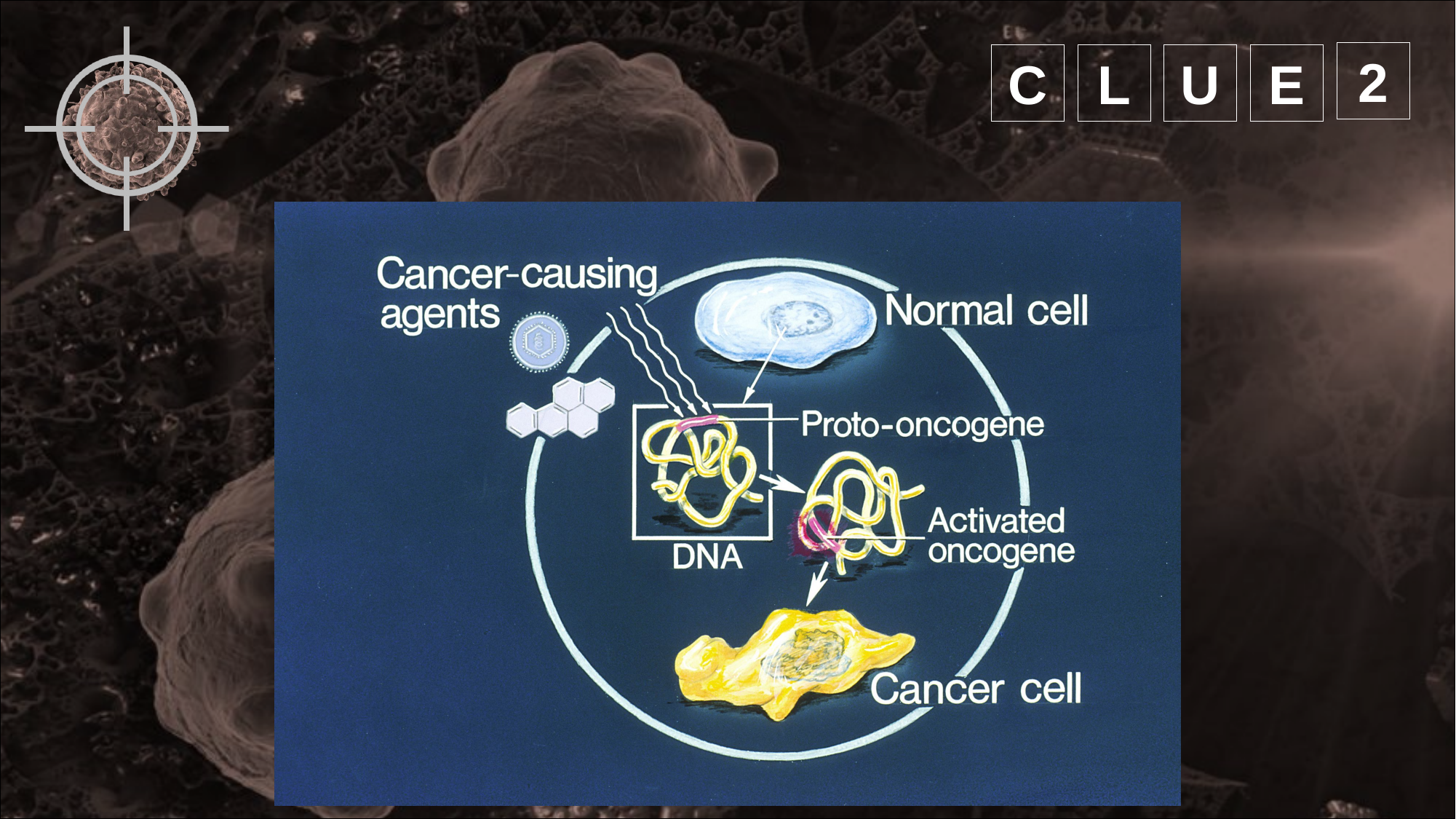

2
C
L
U
E

## Slide 13
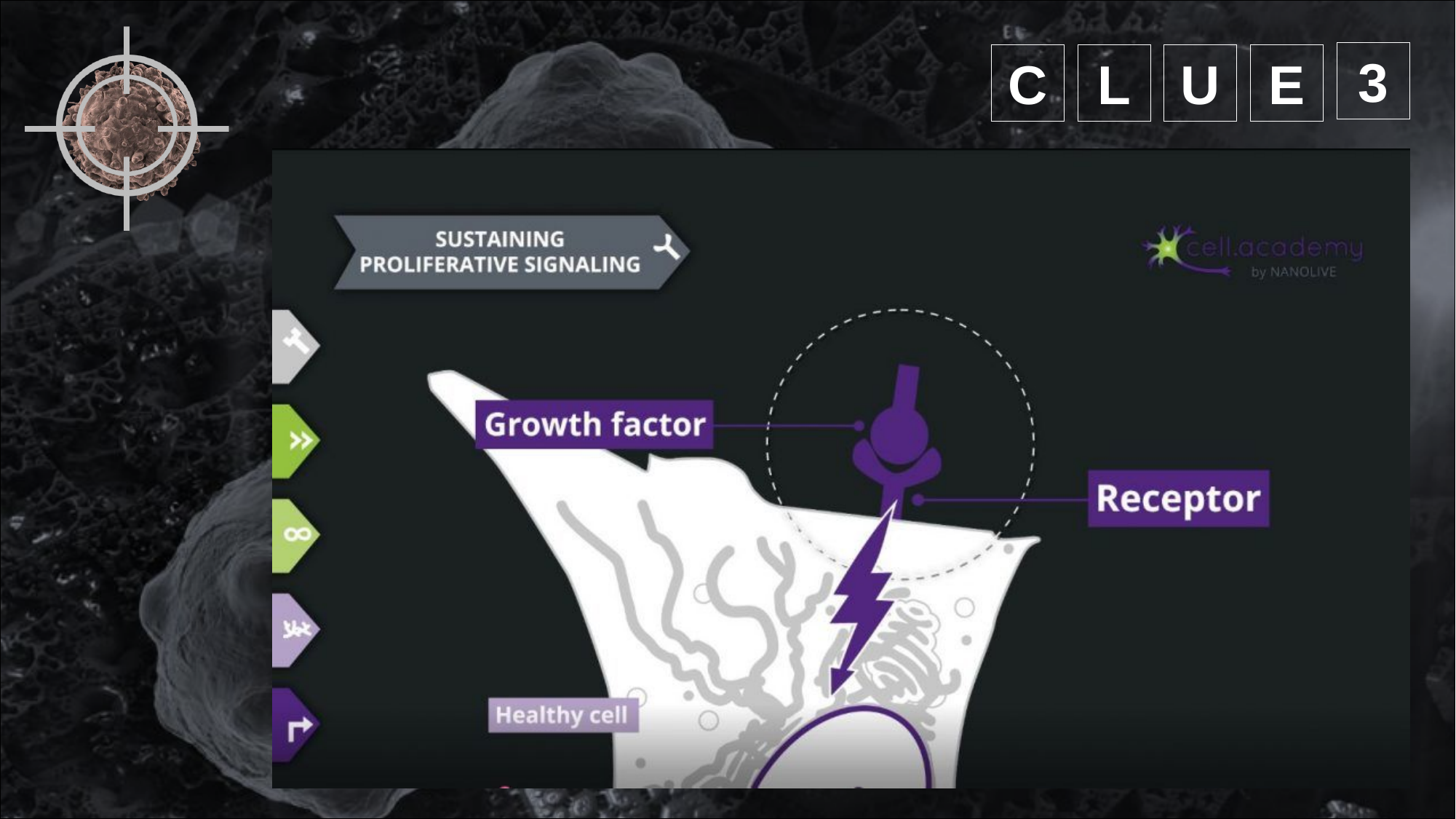

3
C
L
U
E

## Slide 14
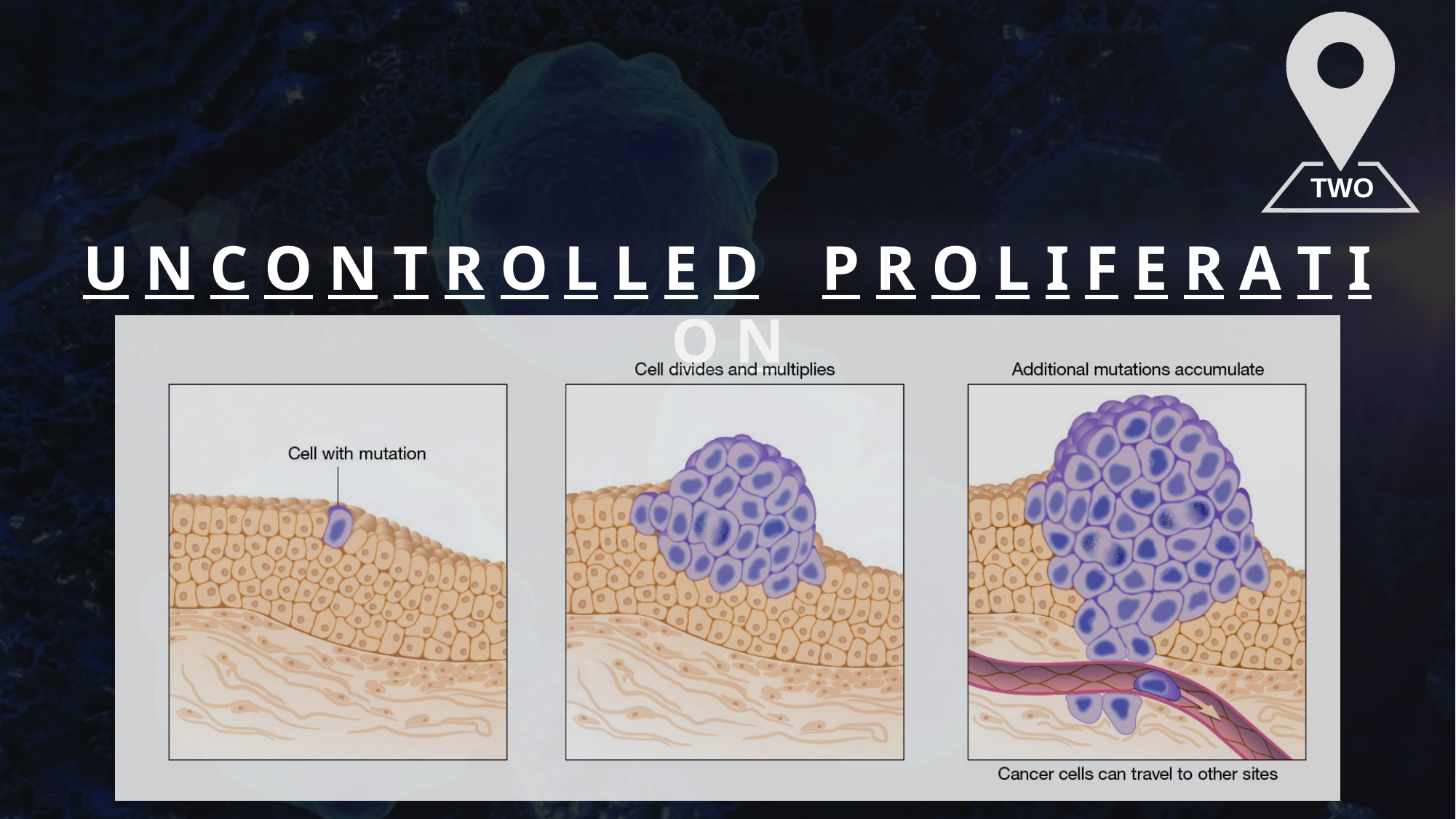

TWO
U n c o n t r o l l e d p r o l I f e r a t I o n

## Slide 15
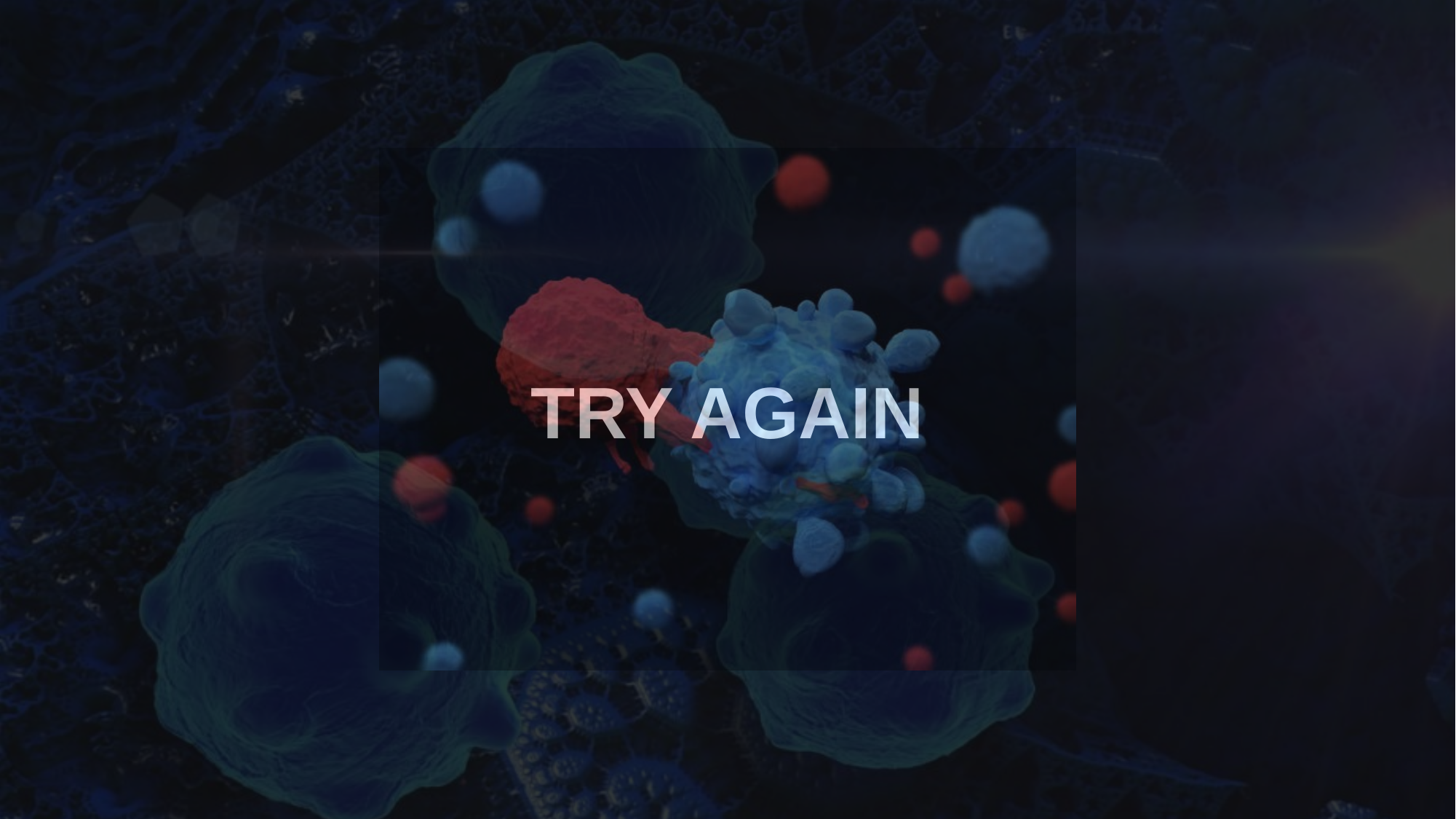

TRY AGAIN

## Slide 16
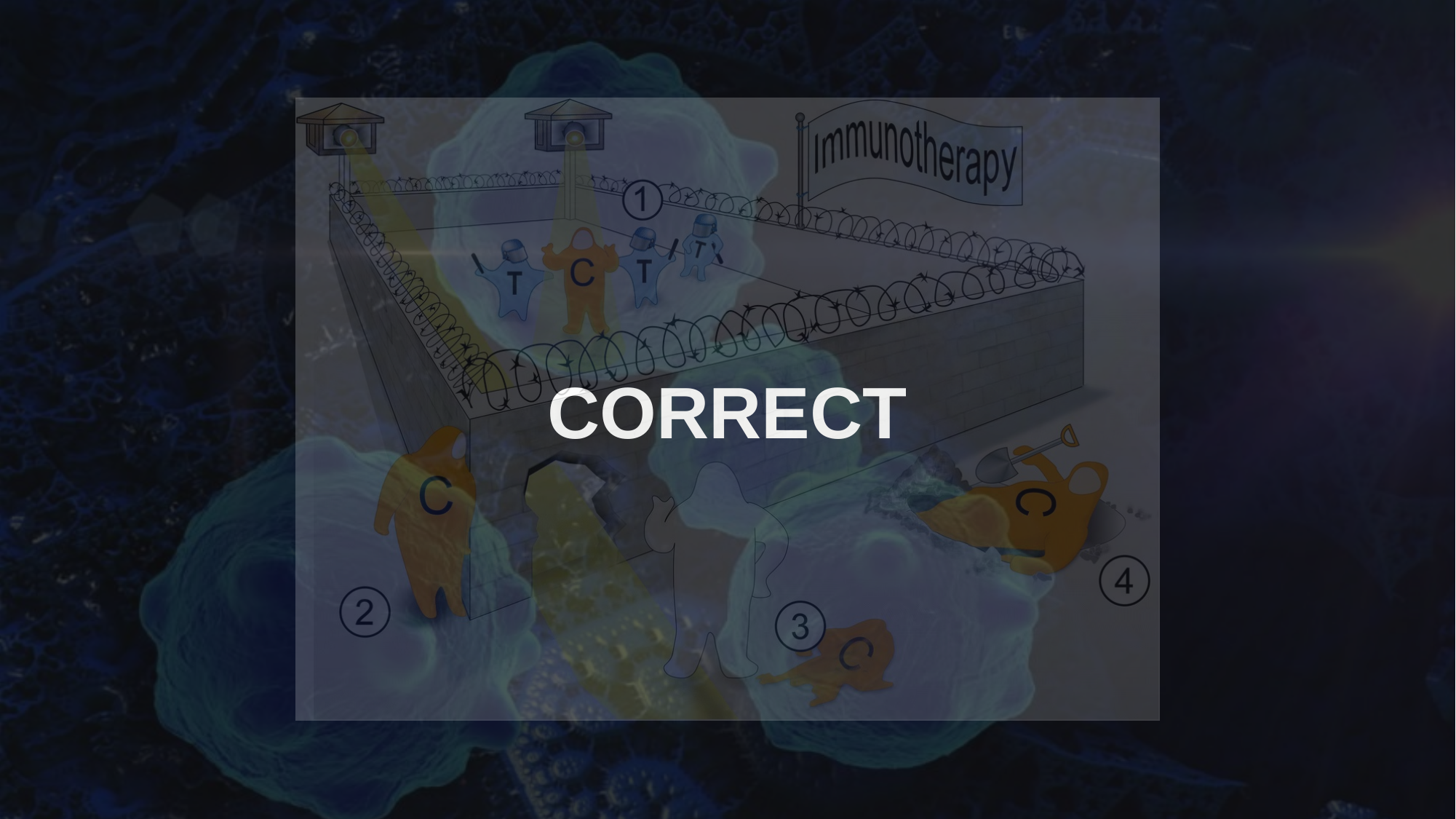

CORRECT

## Slide 17
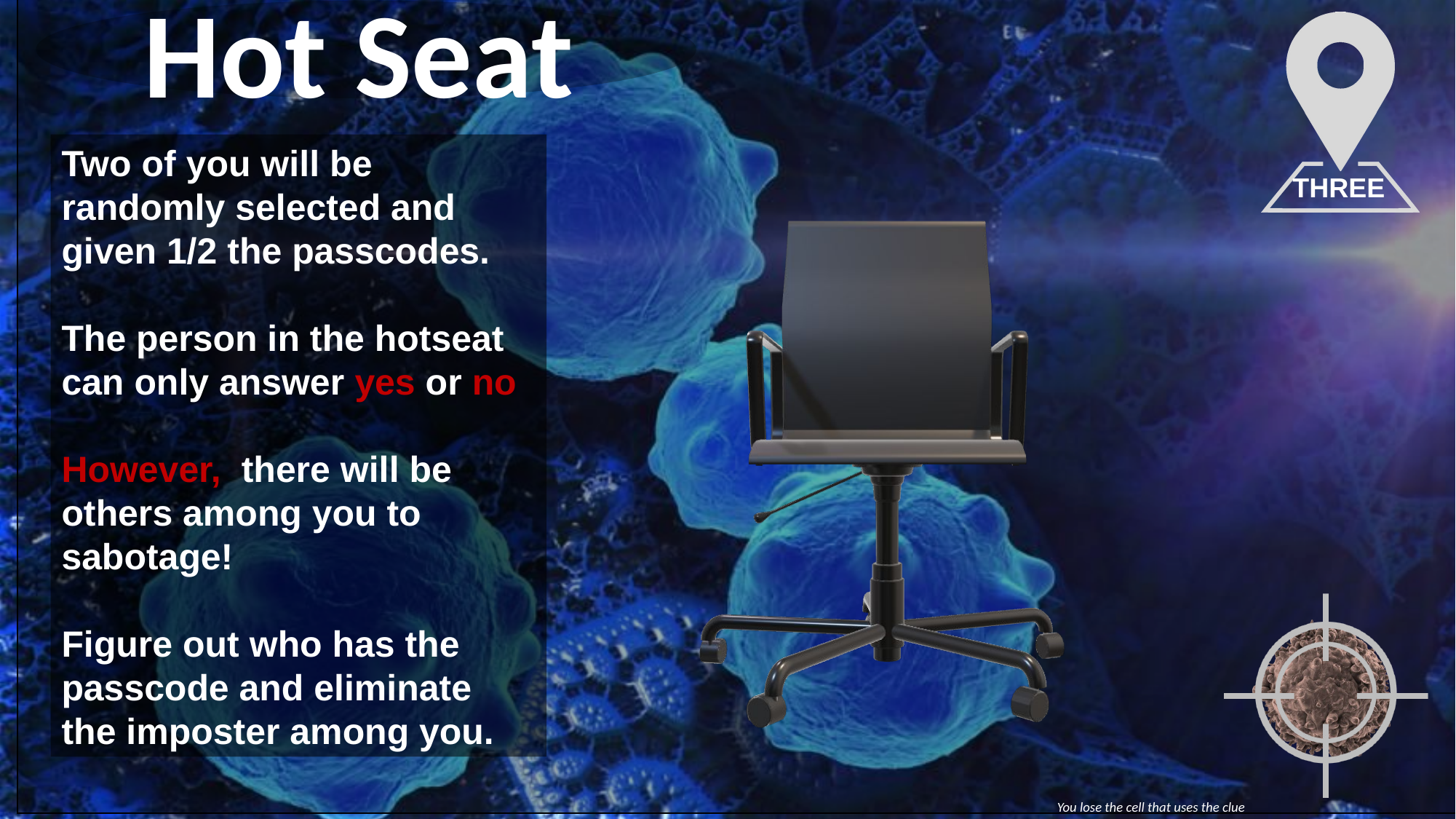

Hot Seat
THREE
Two of you will be randomly selected and given 1/2 the passcodes.
The person in the hotseat can only answer yes or no
However, there will be others among you to sabotage!
Figure out who has the passcode and eliminate the imposter among you.
You lose the cell that uses the clue

## Slide 18
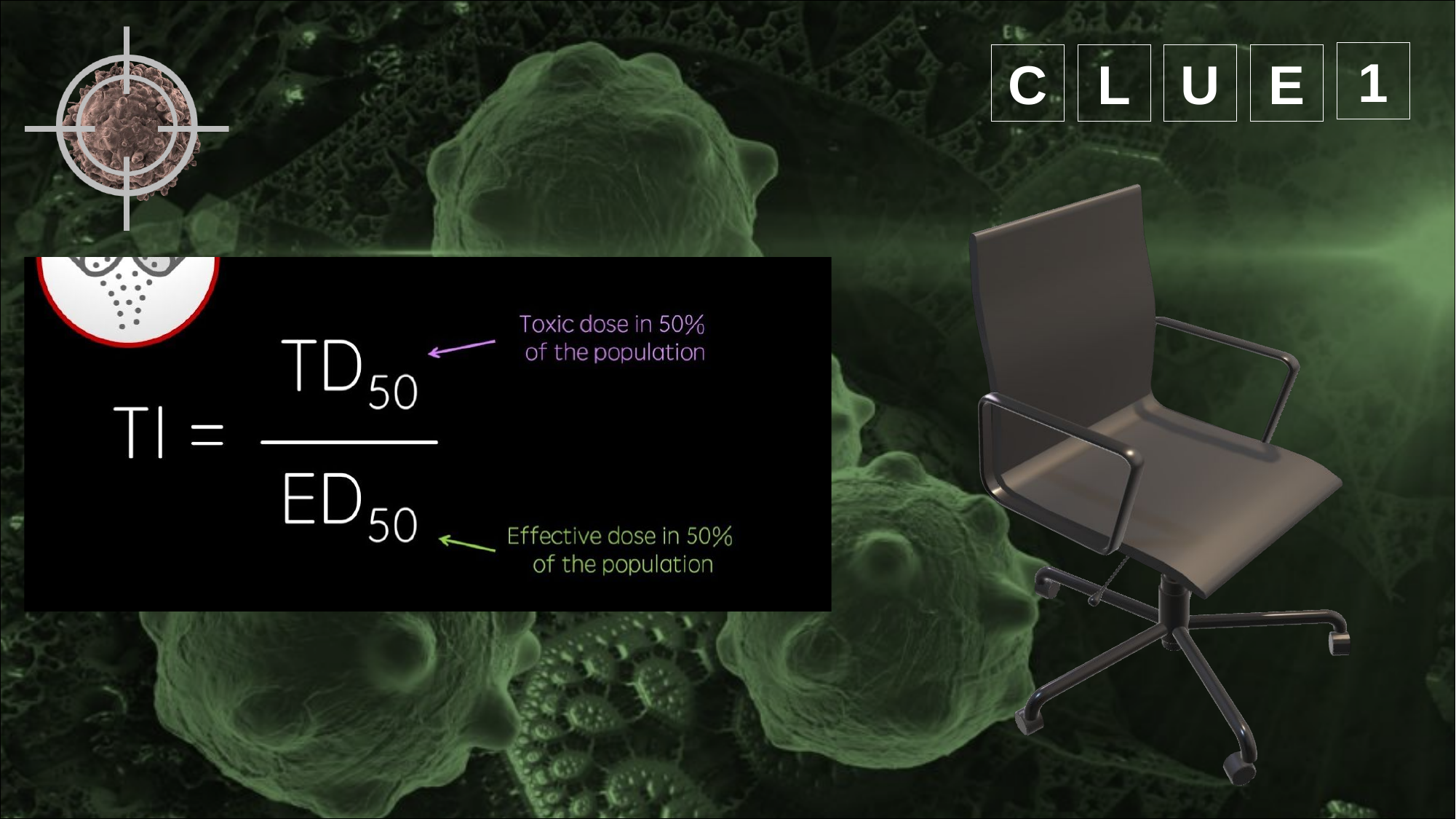

1
C
L
U
E

## Slide 19
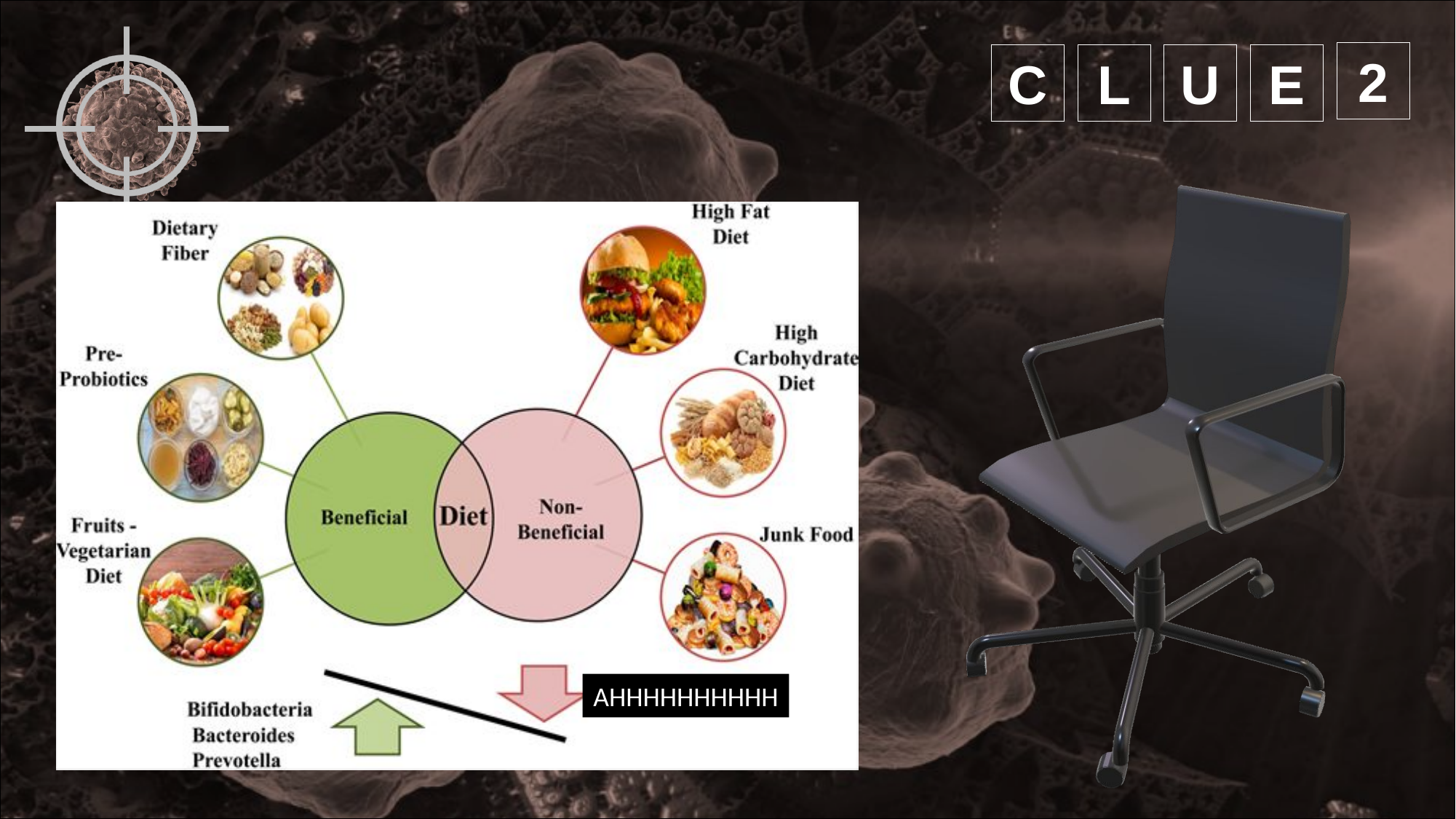

2
C
L
U
E
AHHHHHHHHHH

## Slide 20
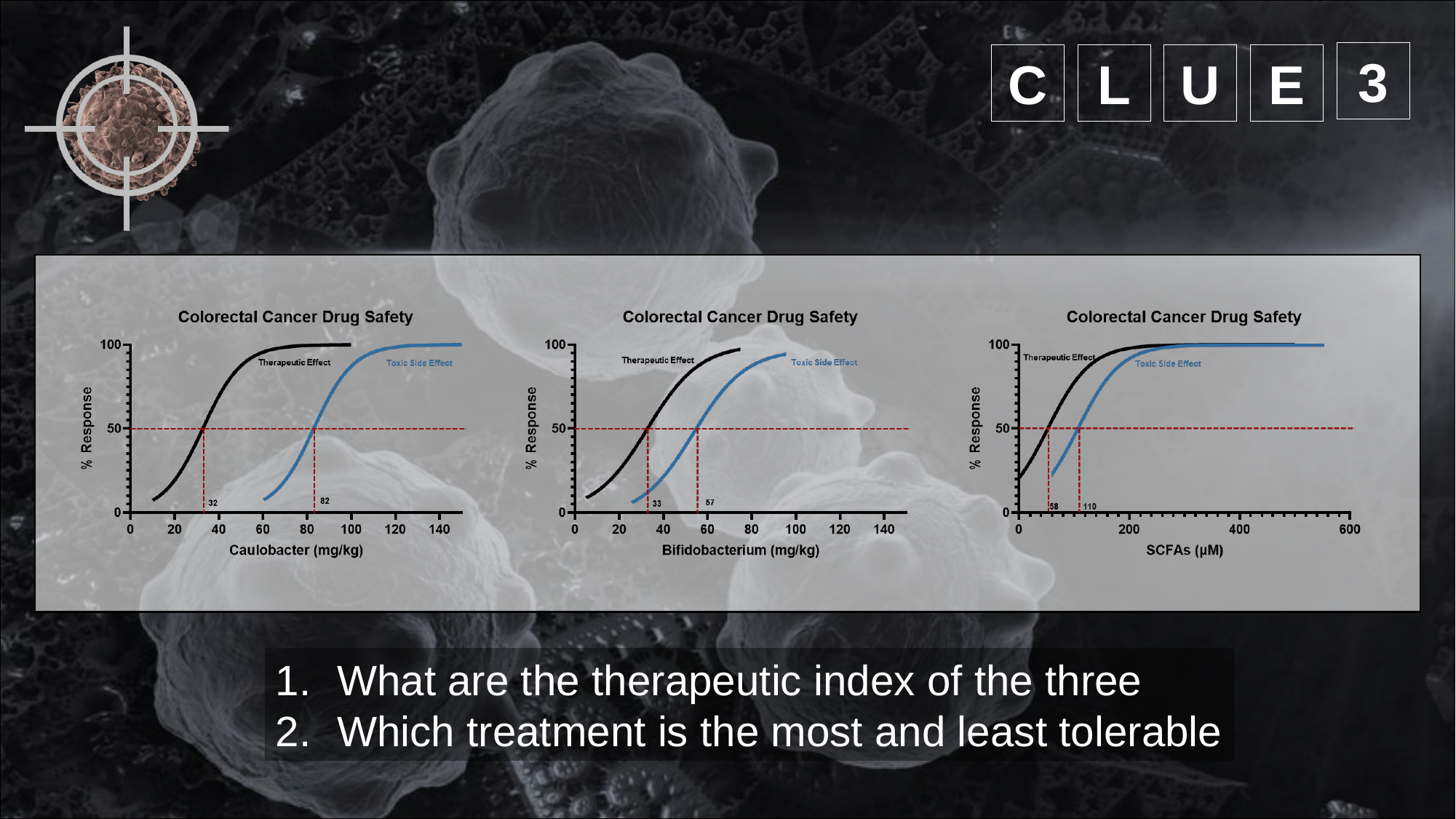

3
C
L
U
E
What are the therapeutic index of the three
Which treatment is the most and least tolerable

## Slide 21
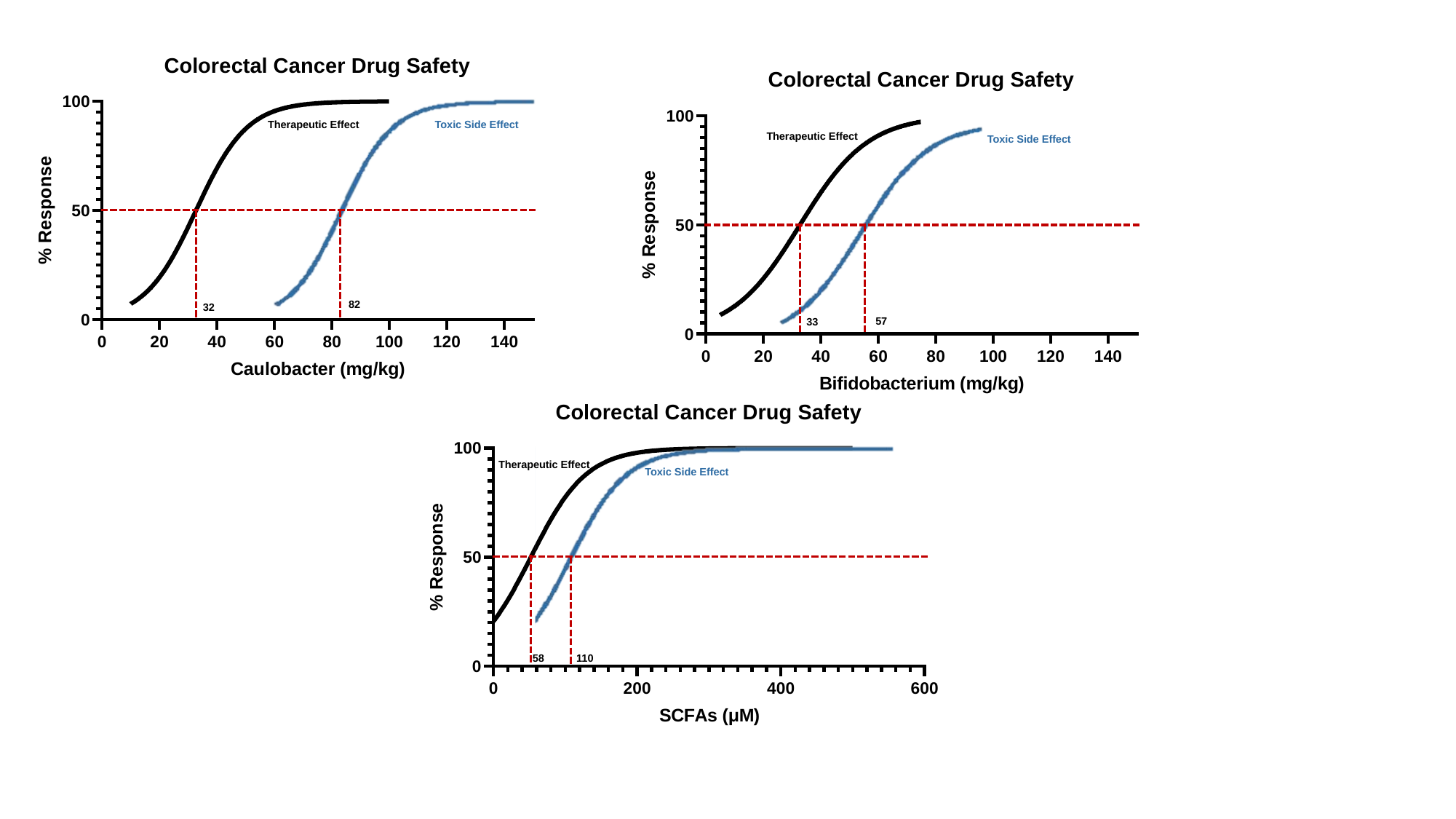

Therapeutic Effect
Toxic Side Effect
82
32
Therapeutic Effect
Toxic Side Effect
57
33
Therapeutic Effect
Toxic Side Effect
110
58

## Slide 22
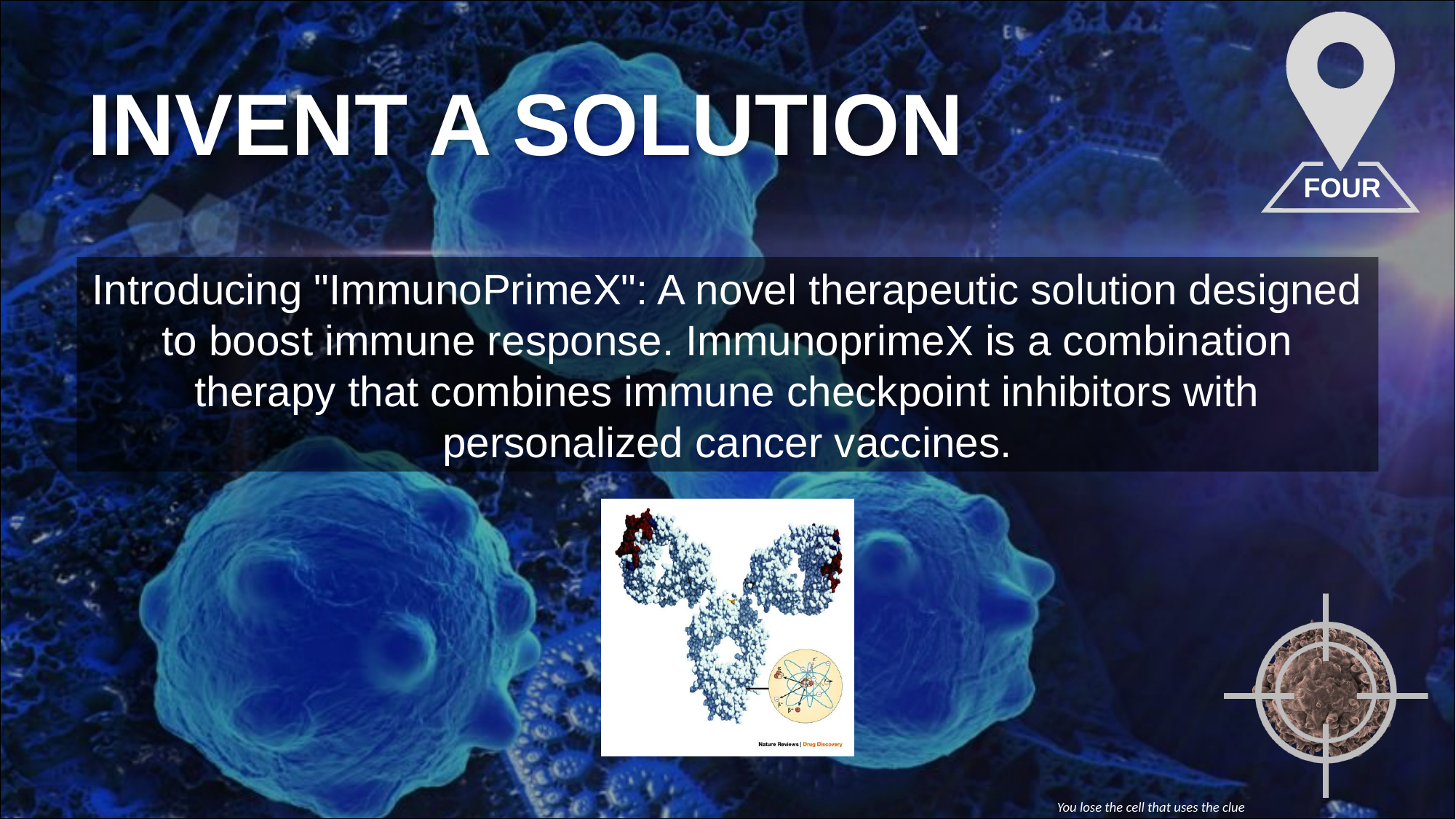

FOUR
Invent A Solution
Introducing "ImmunoPrimeX": A novel therapeutic solution designed to boost immune response. ImmunoprimeX is a combination therapy that combines immune checkpoint inhibitors with personalized cancer vaccines.
You lose the cell that uses the clue

## Slide 23
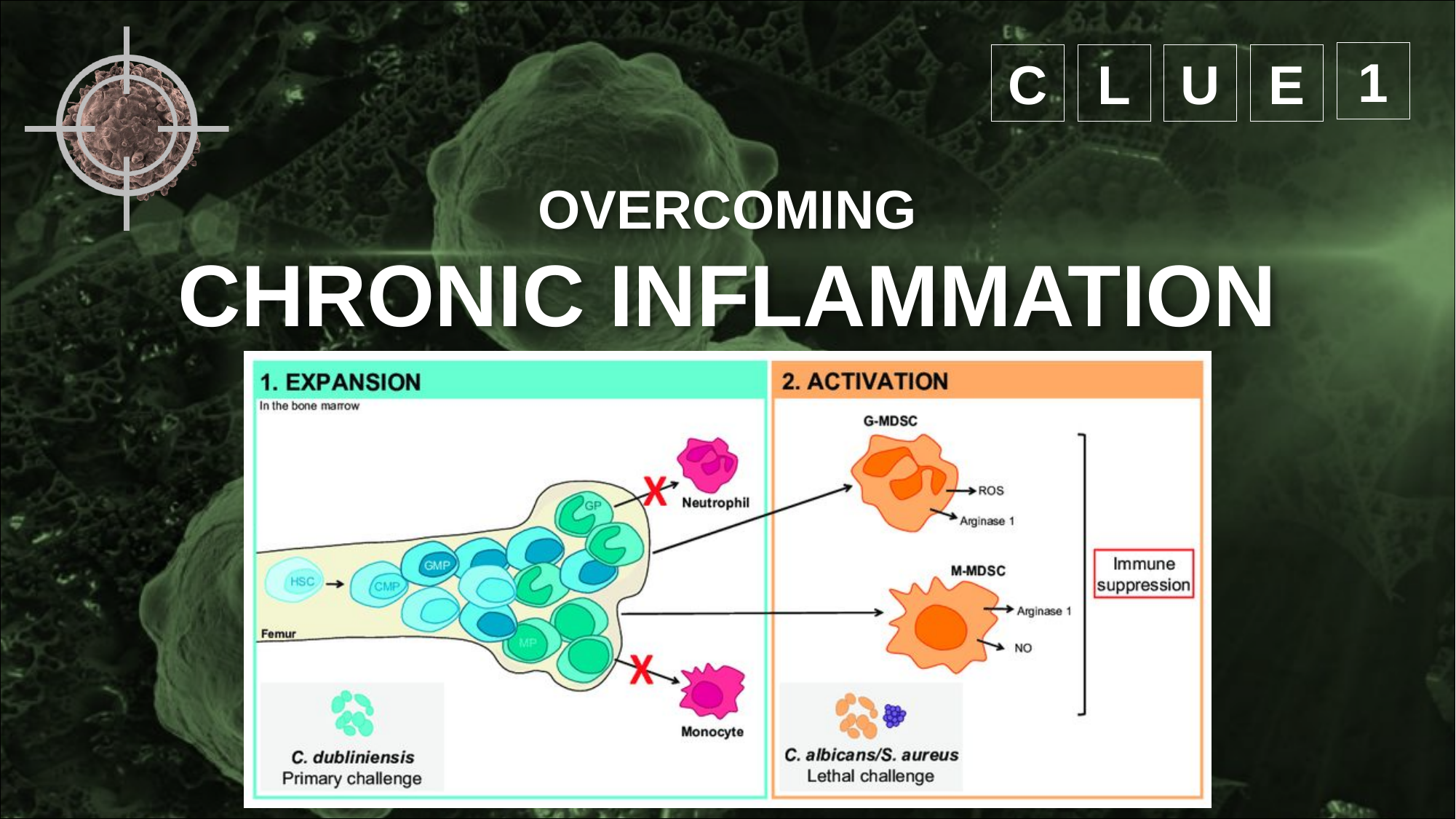

1
C
L
U
E
Overcoming
Chronic Inflammation

## Slide 24
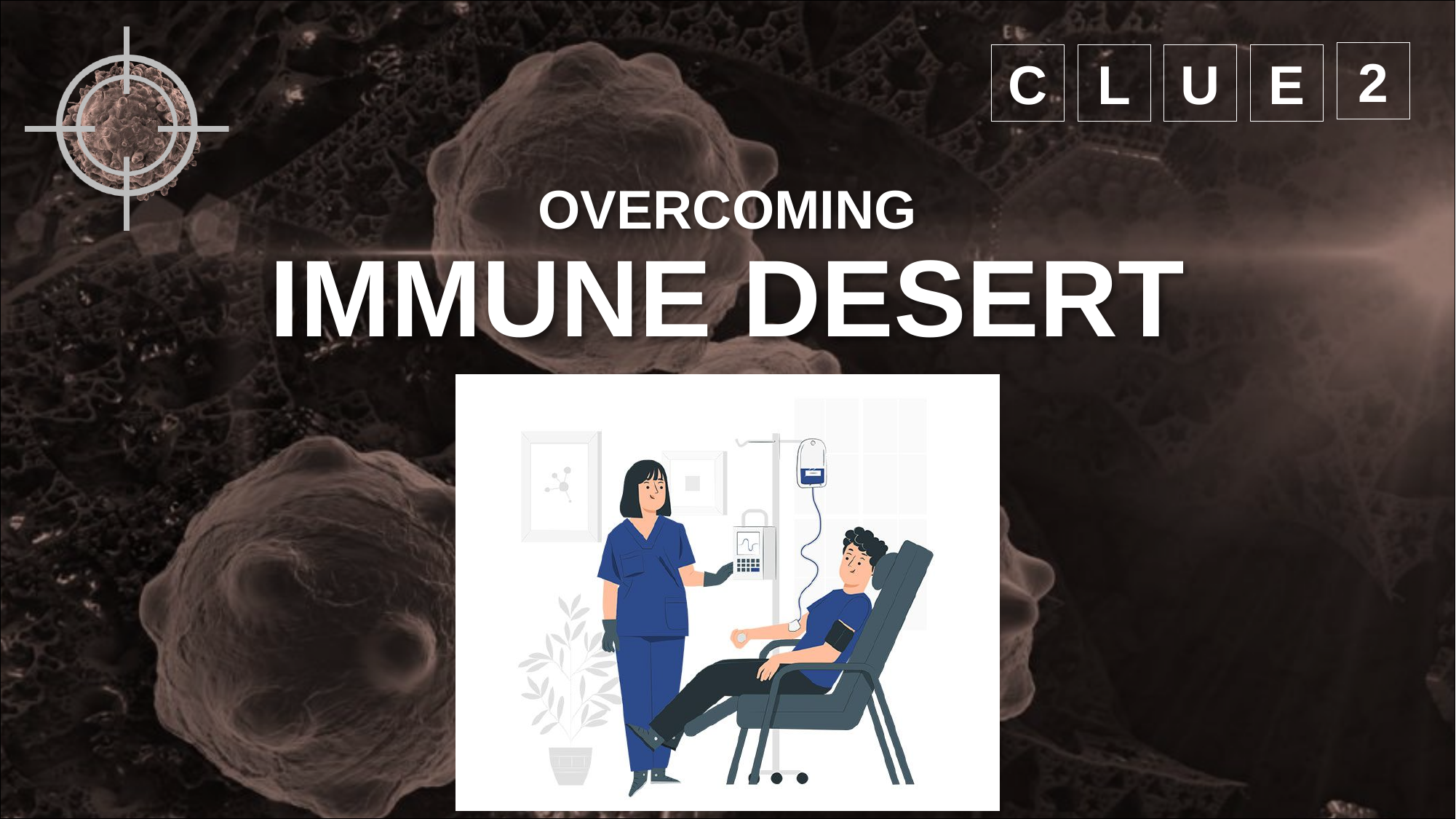

2
C
L
U
E
Overcoming
Immune Desert

## Slide 25
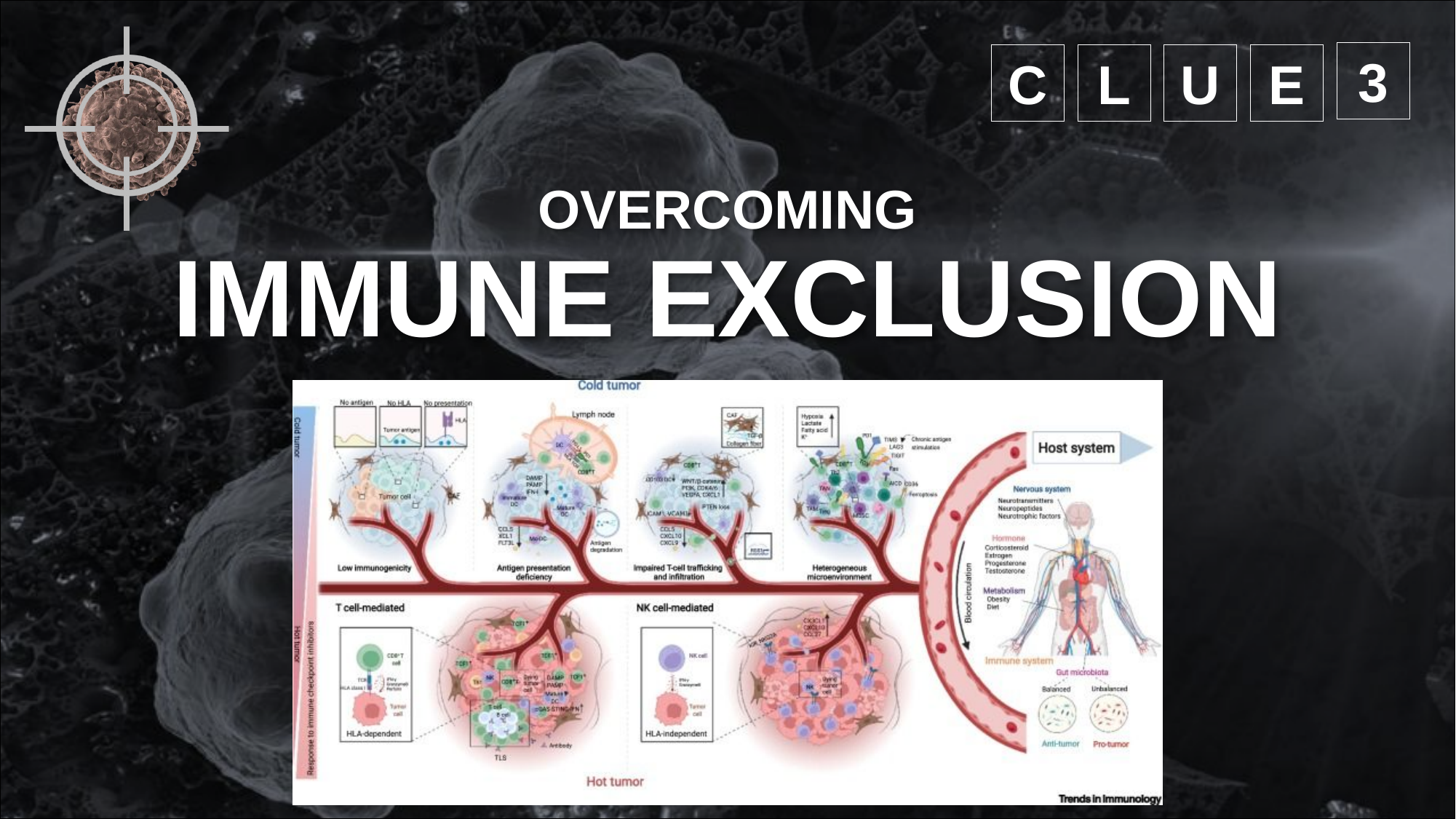

3
C
L
U
E
Overcoming
Immune Exclusion

## Slide 26
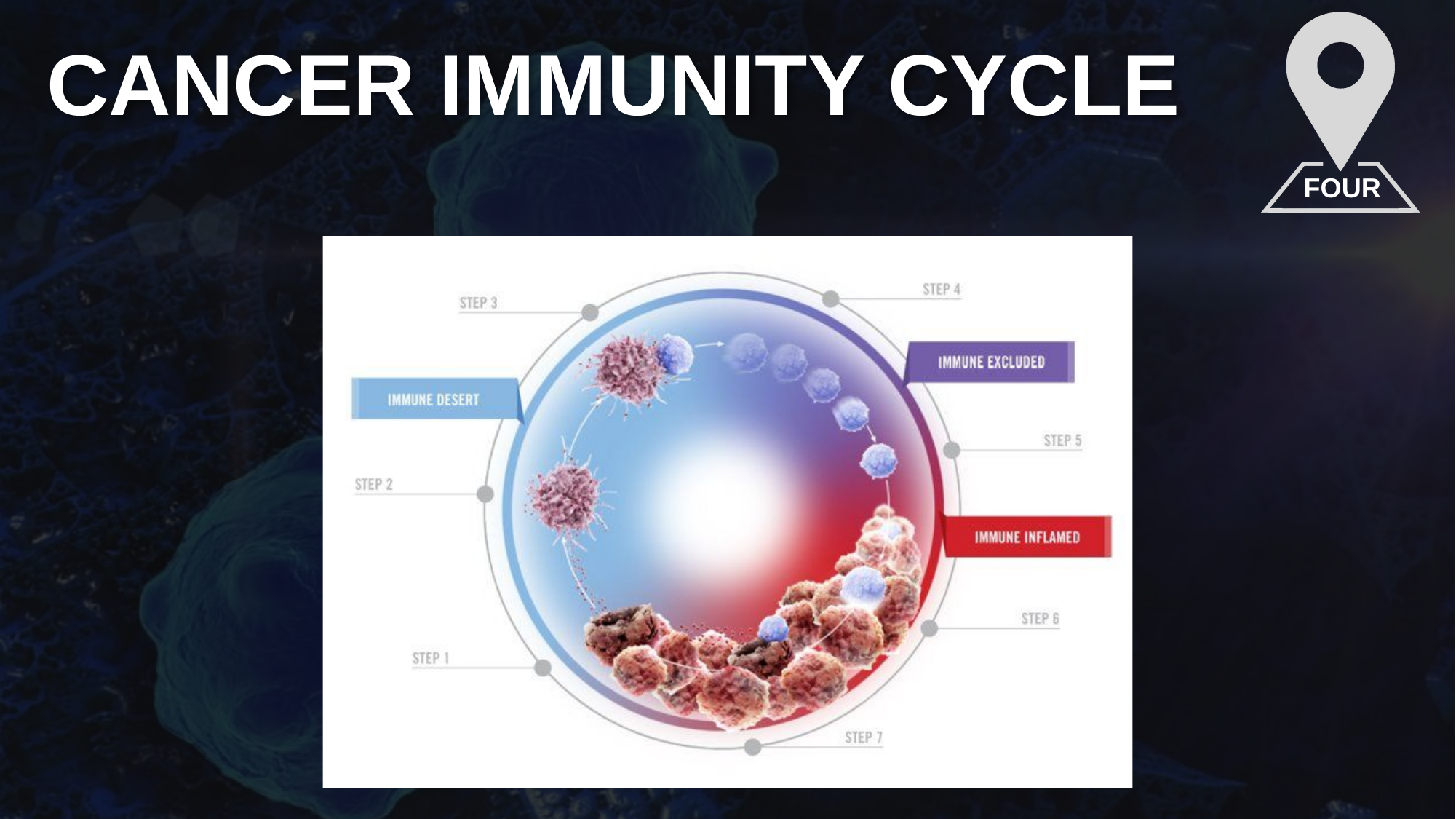

Cancer Immunity Cycle
FOUR

## Slide 27
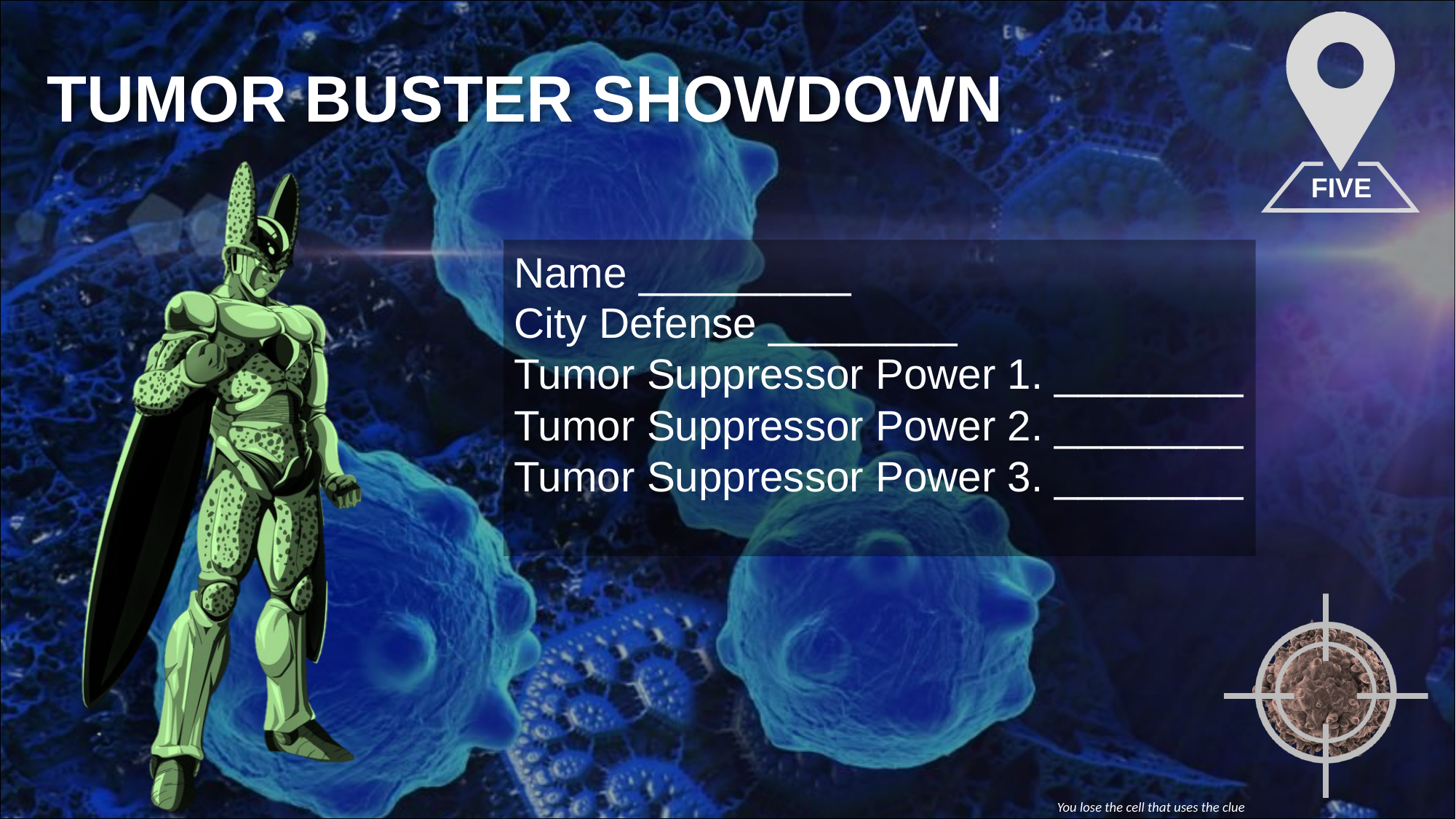

FIVE
Tumor Buster Showdown
Name _________
City Defense ________
Tumor Suppressor Power 1. ________
Tumor Suppressor Power 2. ________
Tumor Suppressor Power 3. ________
You lose the cell that uses the clue

## Slide 28
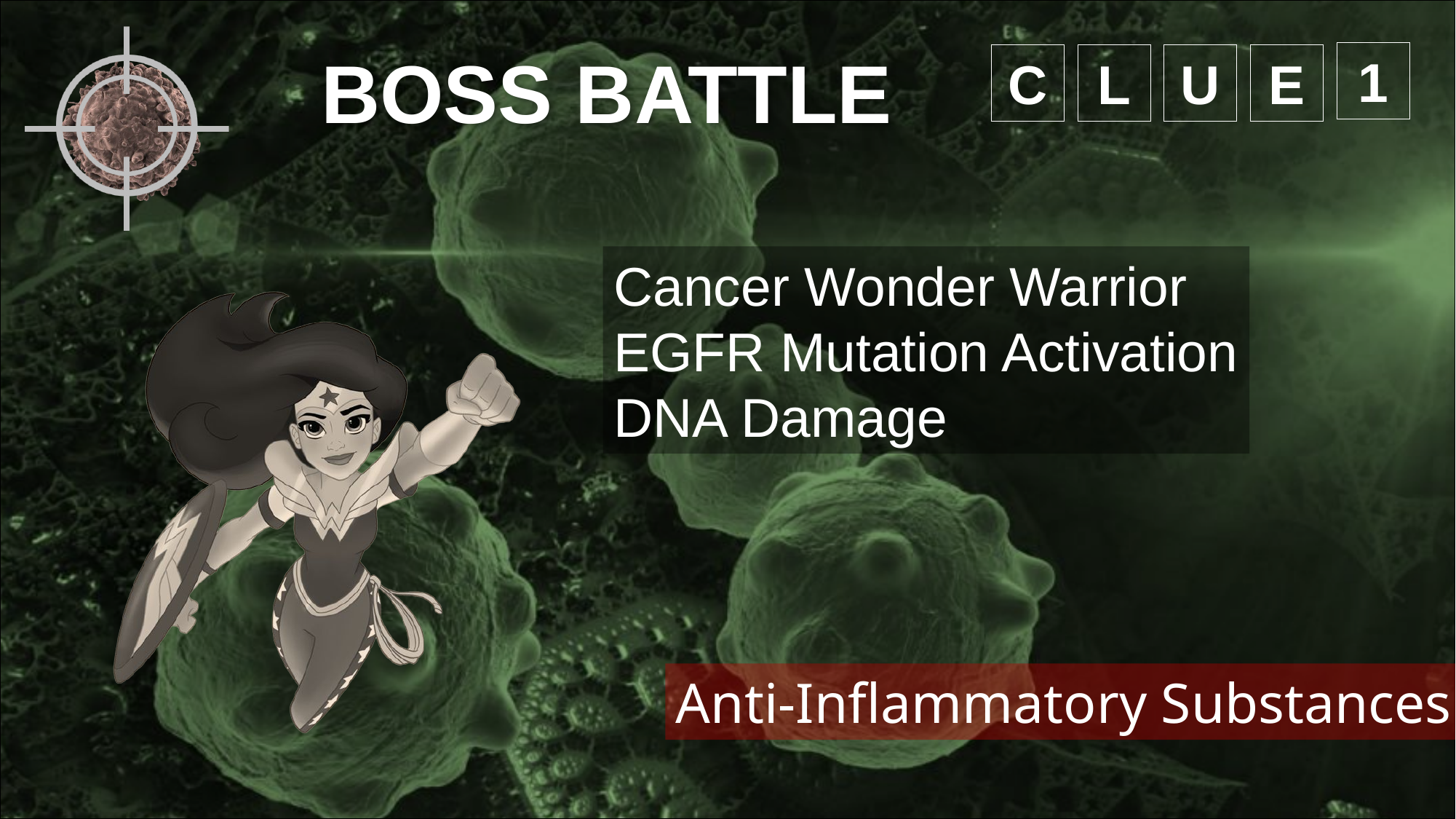

BOSS BATTLE
1
C
L
U
E
Cancer Wonder Warrior
EGFR Mutation Activation
DNA Damage
Anti-Inflammatory Substances

## Slide 29
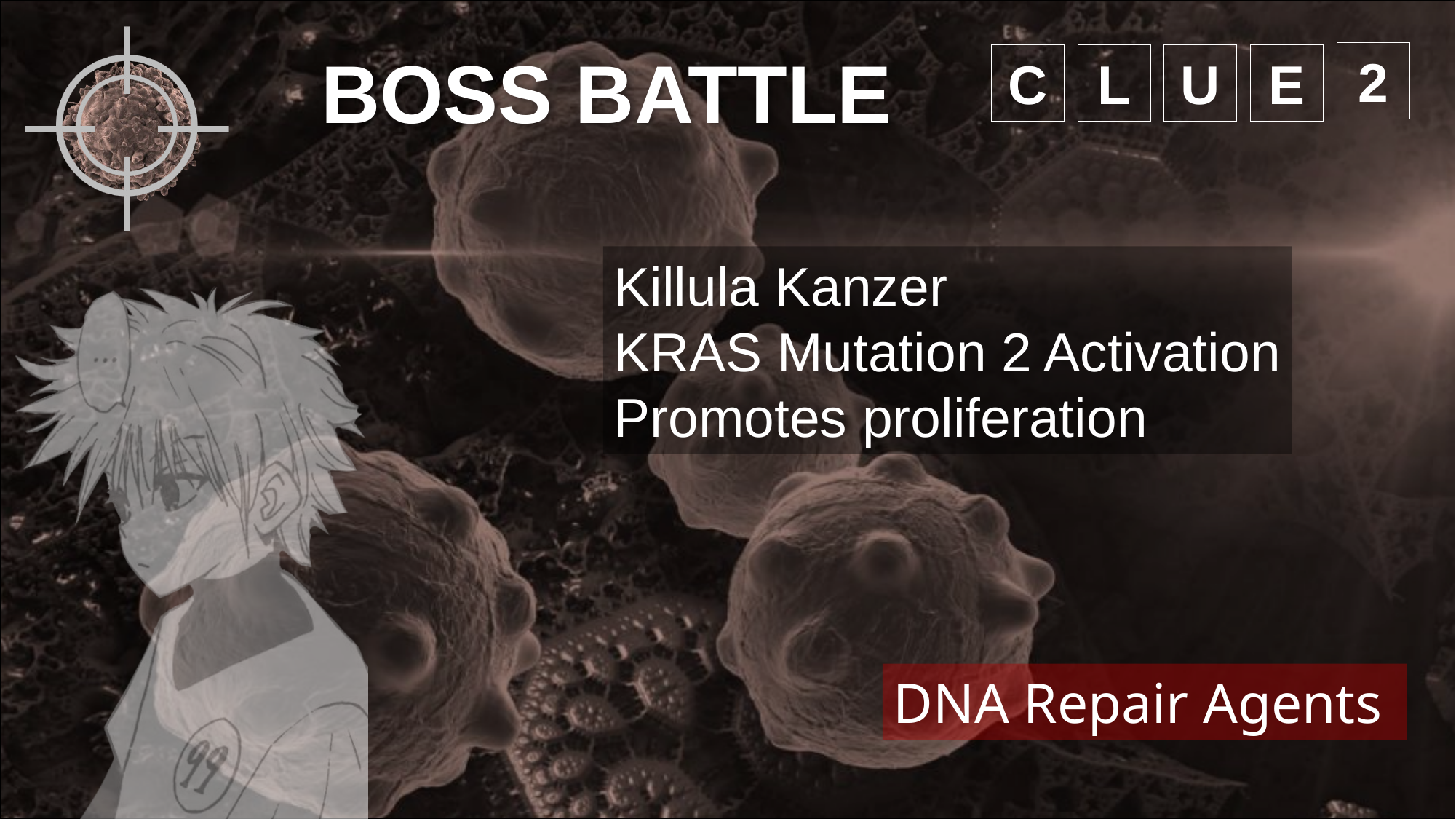

BOSS BATTLE
2
C
L
U
E
Killula Kanzer
KRAS Mutation 2 Activation
Promotes proliferation
DNA Repair Agents

## Slide 30
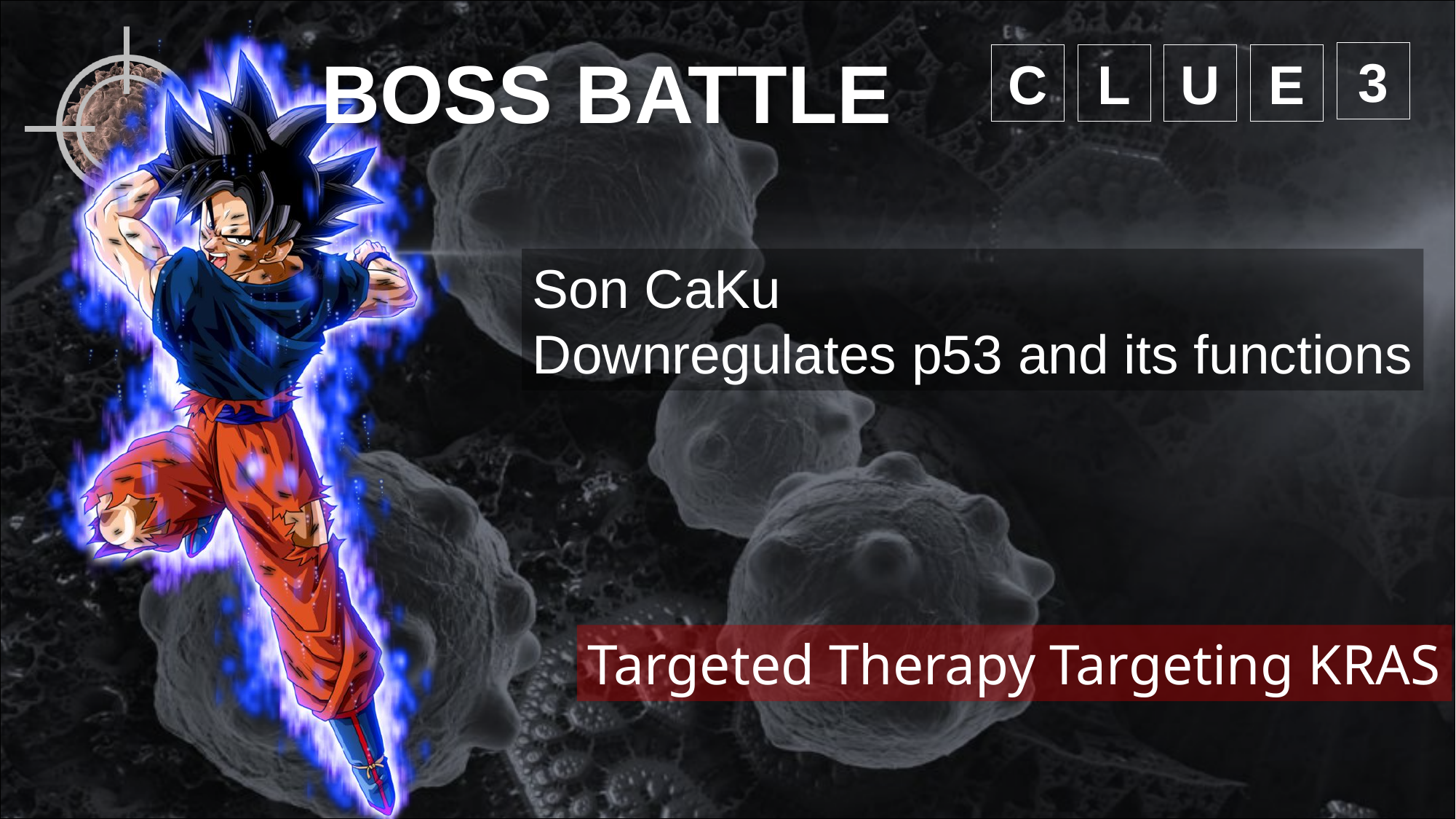

Bypass anti-survival mechanism
BOSS BATTLE
3
C
L
U
E
Son CaKu
Downregulates p53 and its functions
Targeted Therapy Targeting KRAS

## Slide 31
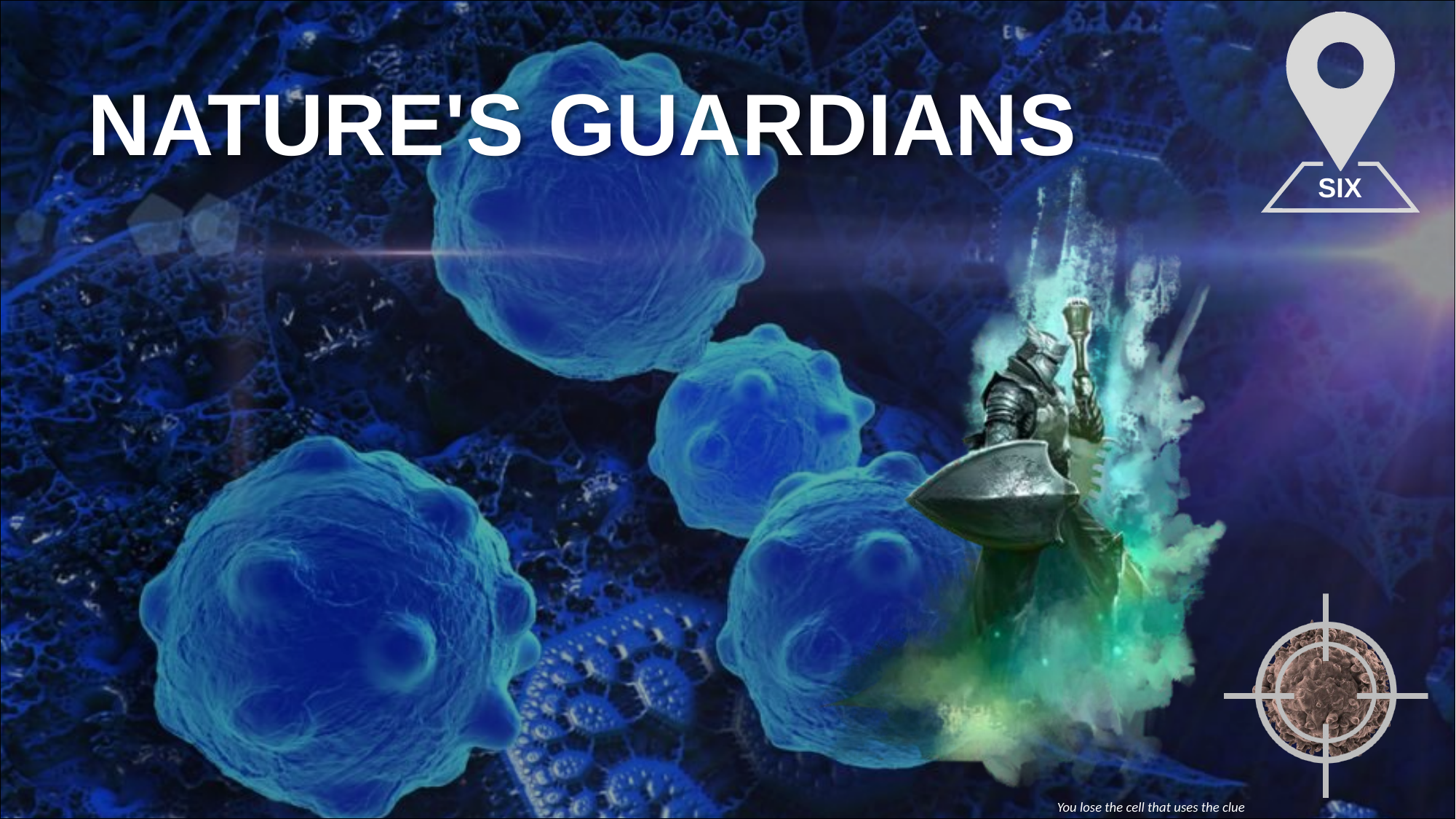

SIX
Nature's Guardians
You lose the cell that uses the clue

## Slide 32
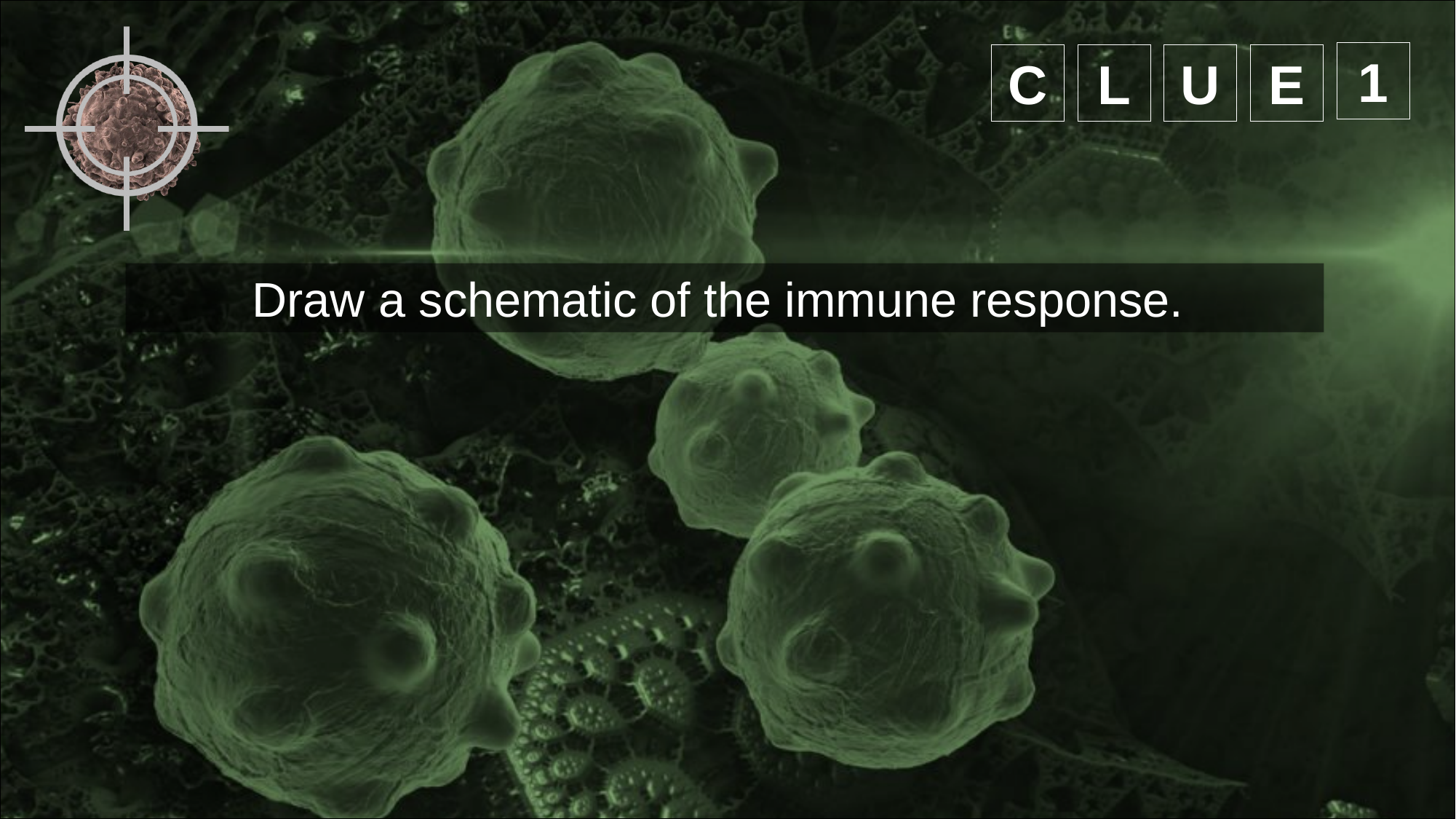

1
C
L
U
E
Draw a schematic of the immune response.

## Slide 33
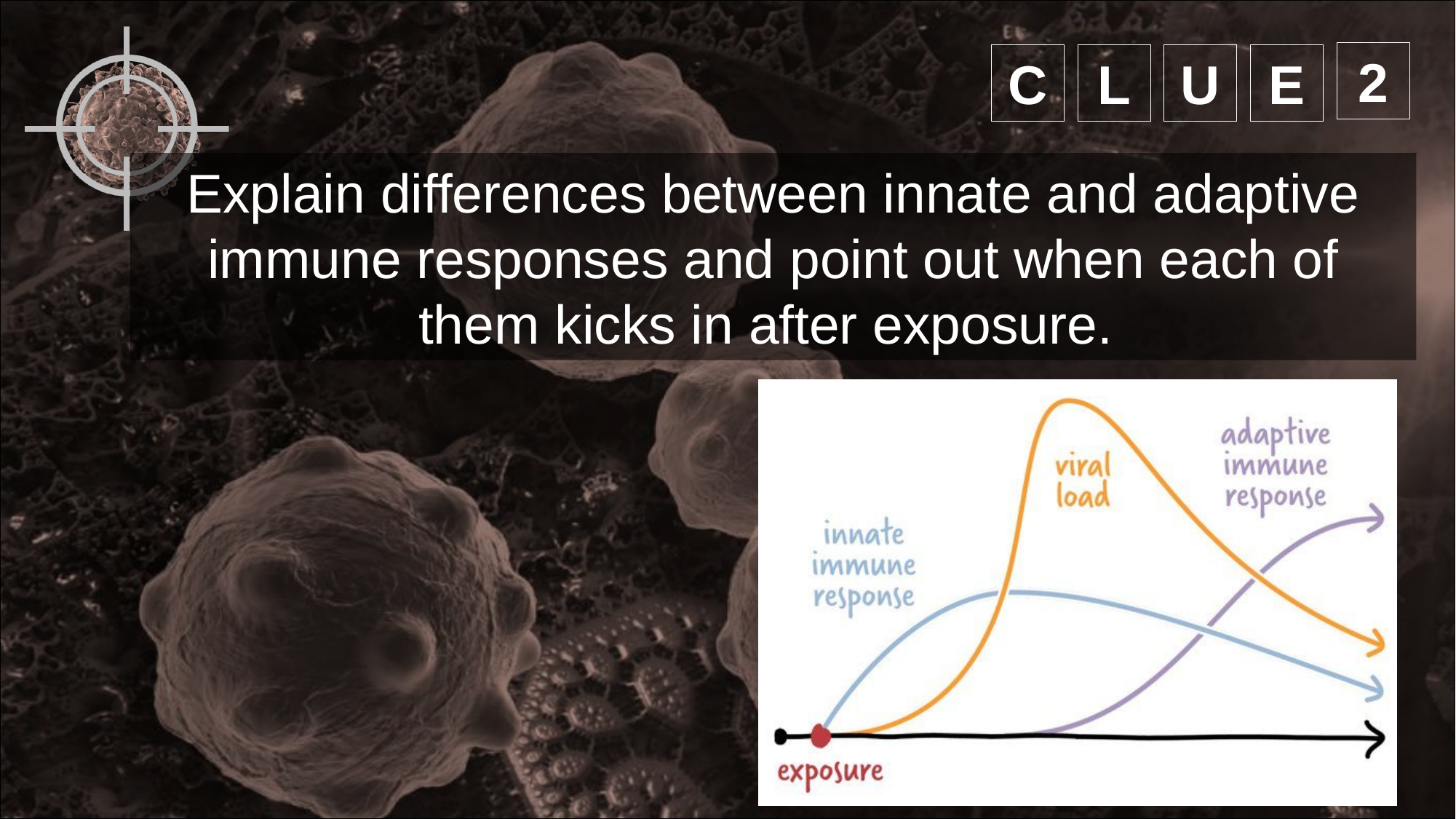

2
C
L
U
E
Explain differences between innate and adaptive immune responses and point out when each of them kicks in after exposure.

## Slide 34
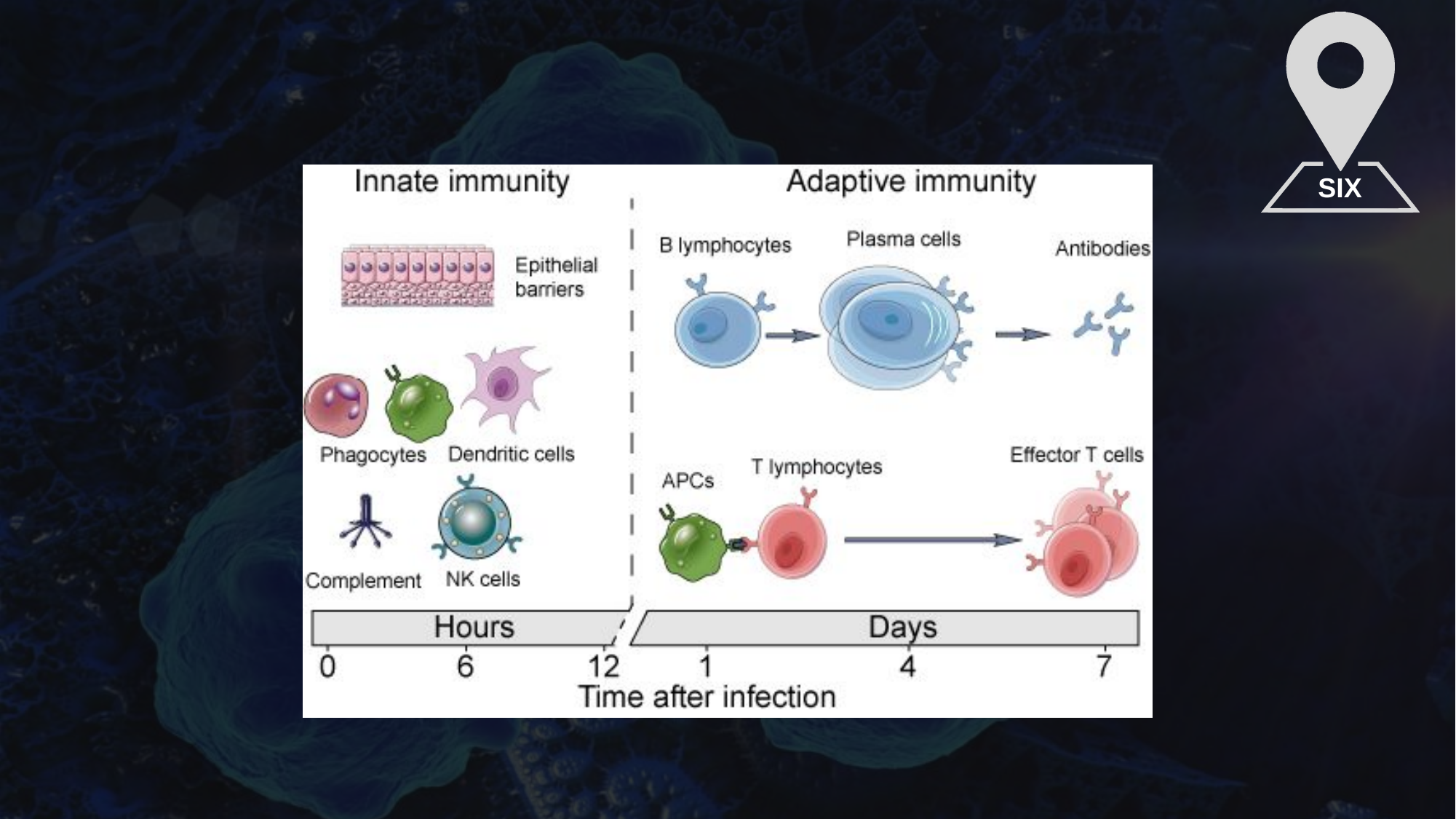

SIX

## Slide 35
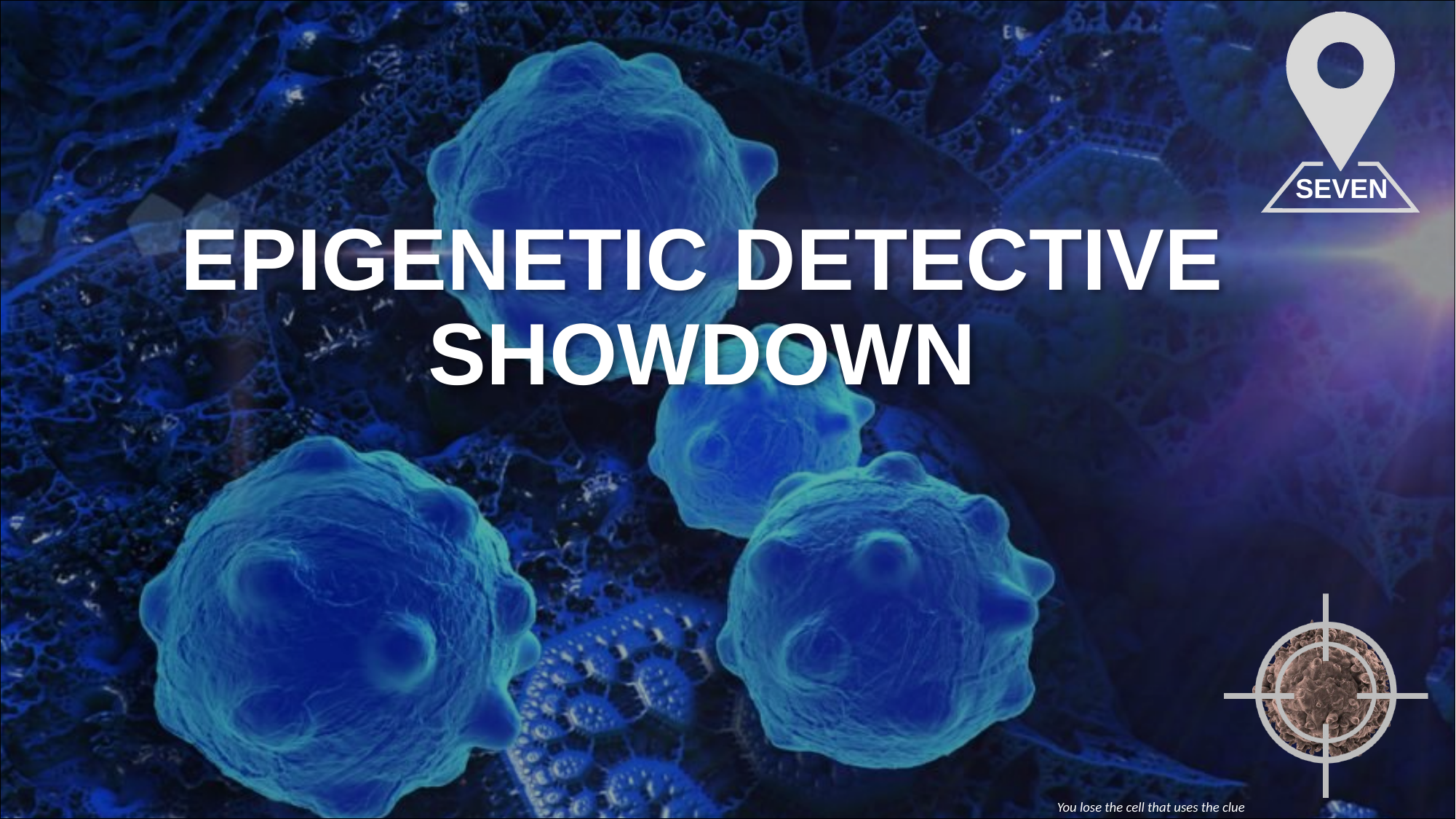

SEVEN
Epigenetic Detective Showdown
You lose the cell that uses the clue

## Slide 36
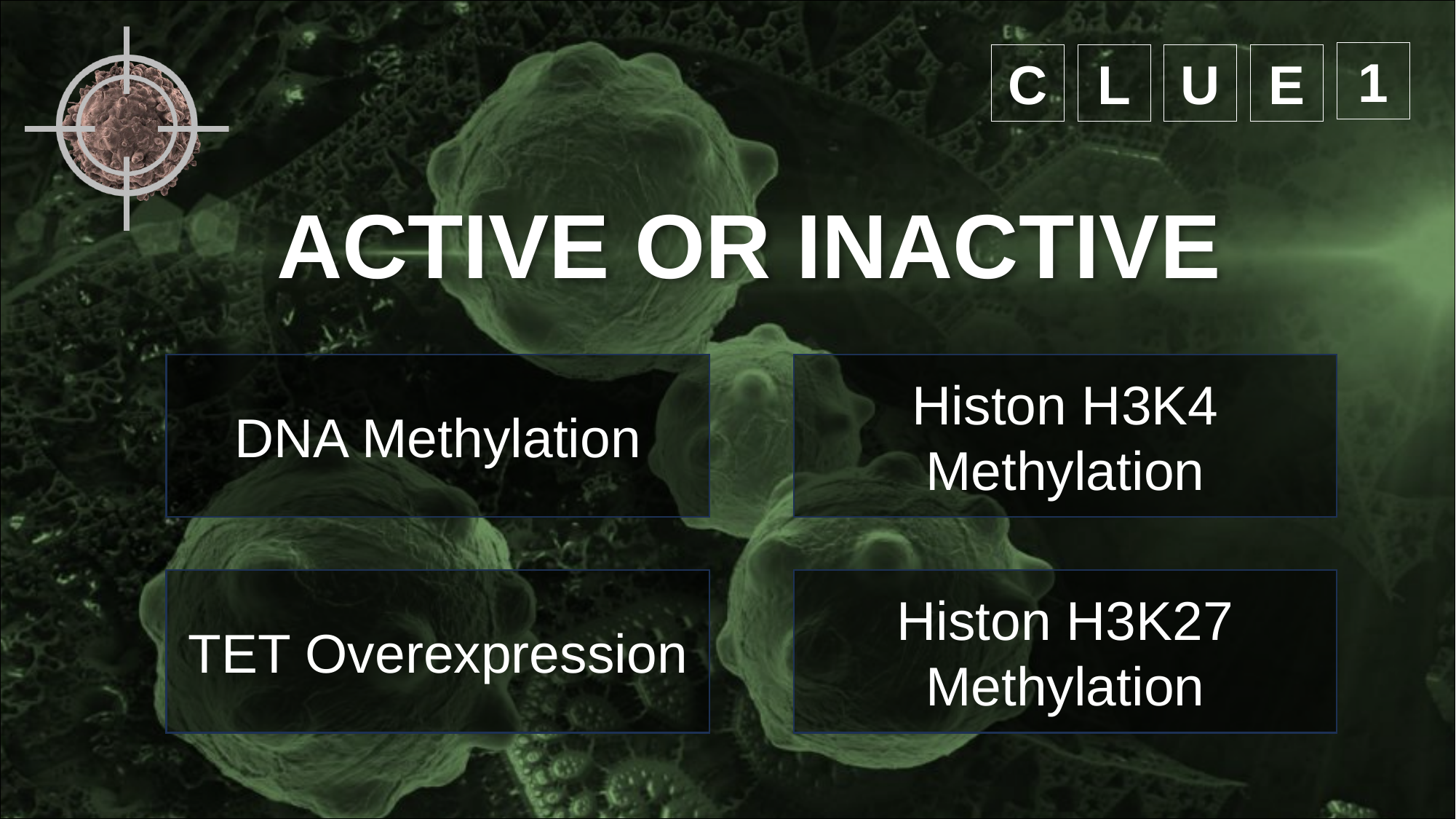

1
C
L
U
E
Active or Inactive
DNA Methylation
Histon H3K4 Methylation
Histon H3K27 Methylation
TET Overexpression

## Slide 37
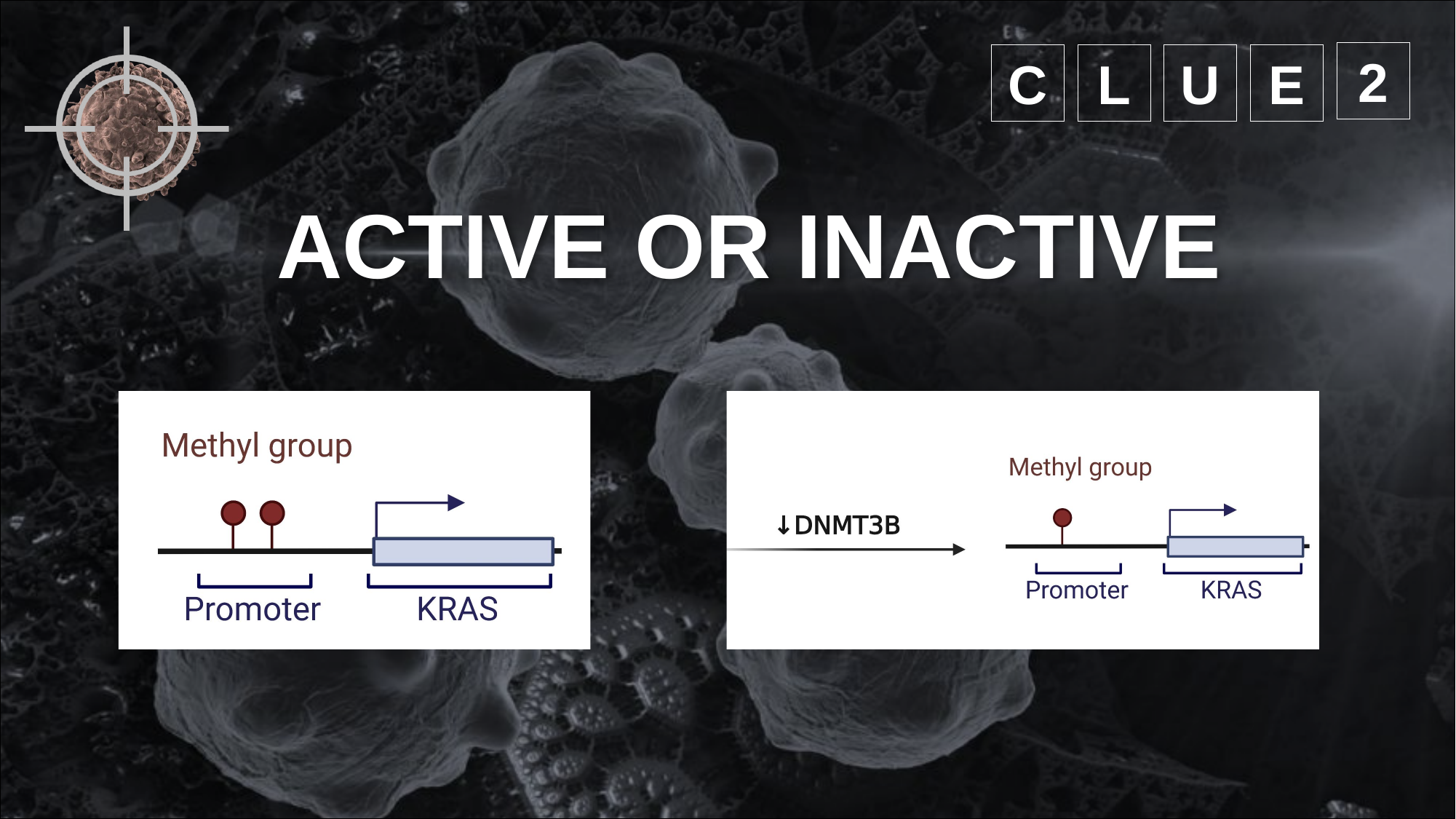

2
C
L
U
E
Active or Inactive

## Slide 38
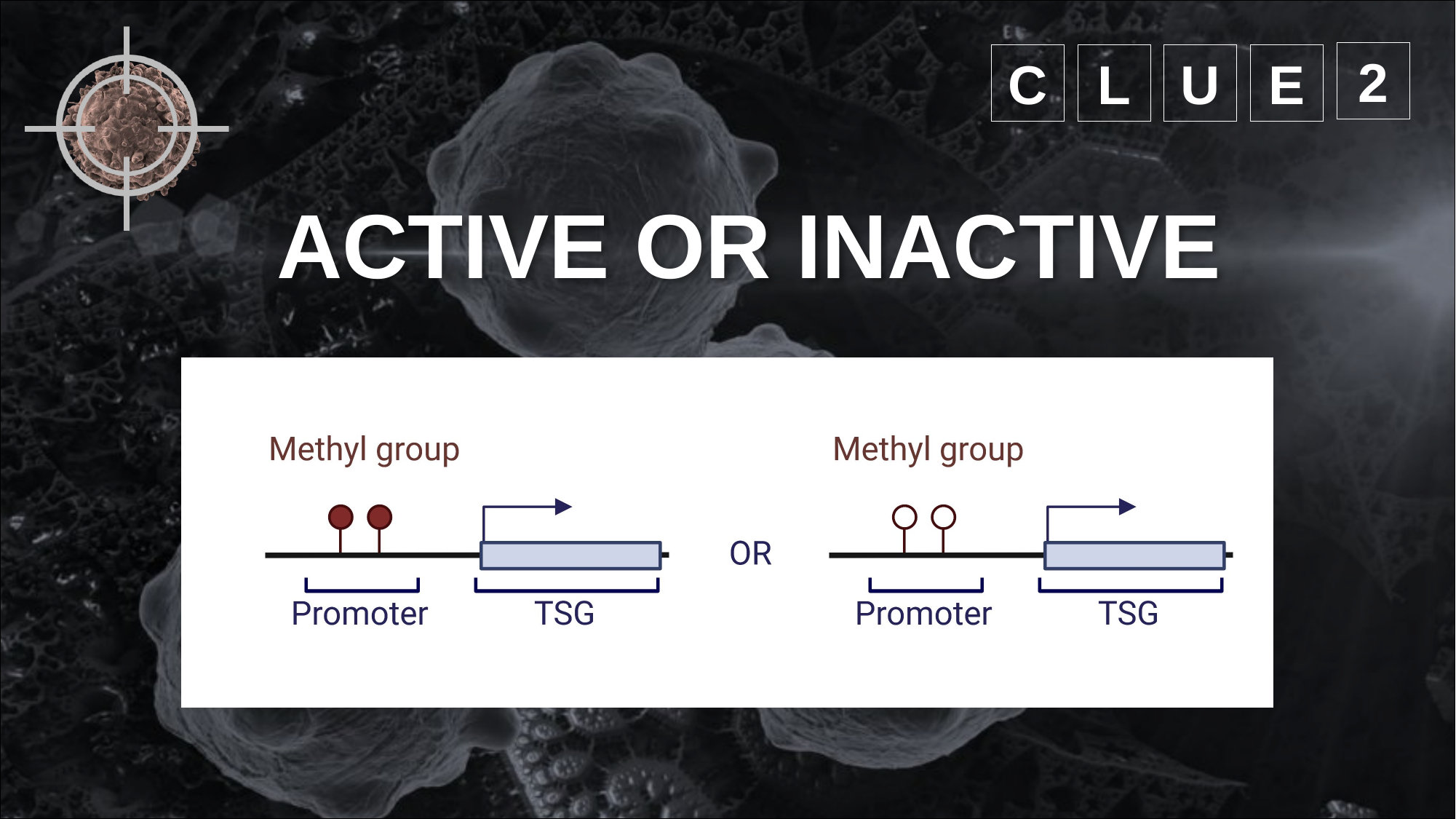

2
C
L
U
E
Active or Inactive

## Slide 39
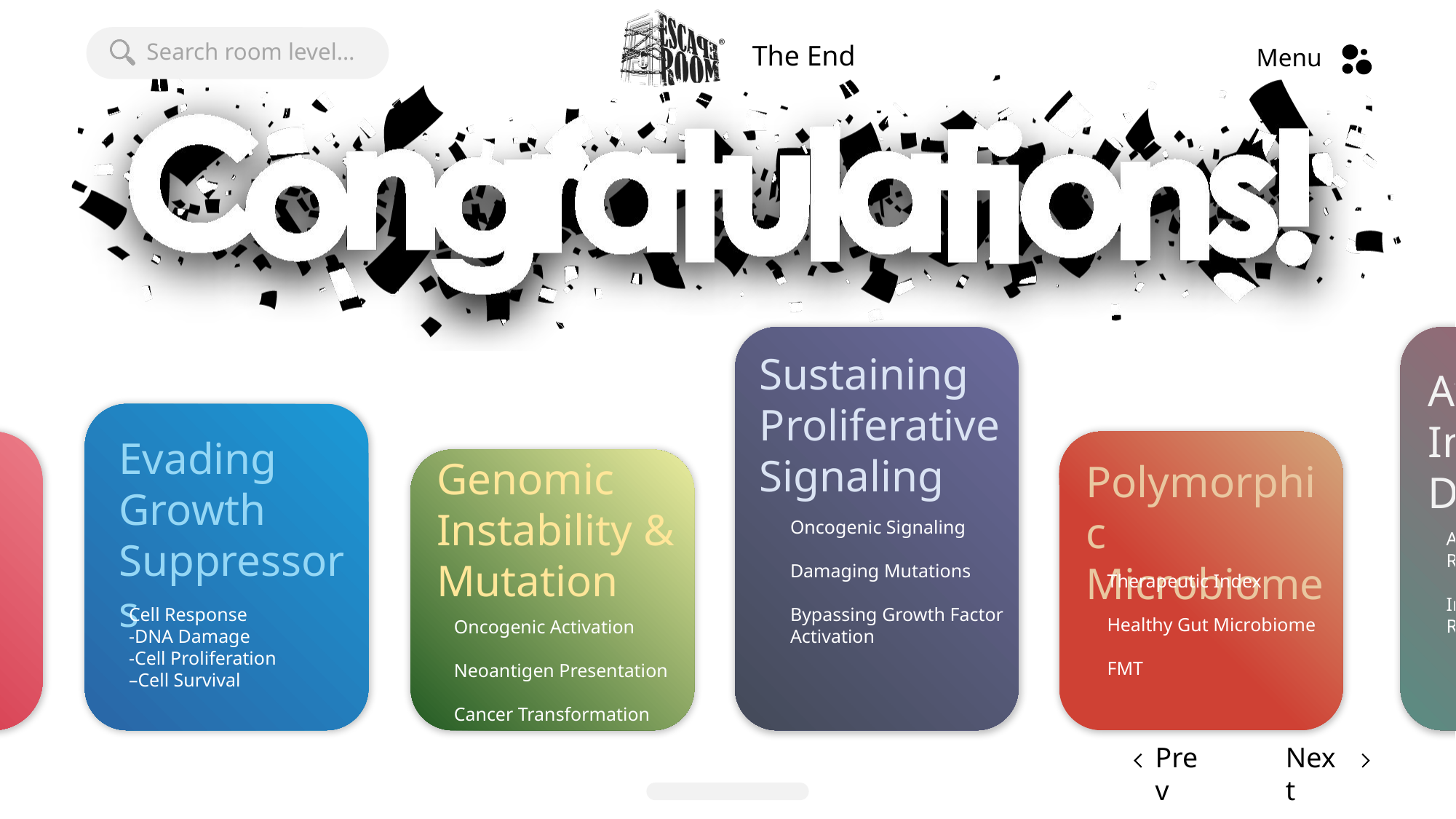

Search room level…
The End
Menu
Sustaining Proliferative Signaling
Avoiding Immune Destruction
Nonmutational Epigenetic Reprogramming
Evading Growth Suppressors
Avoiding Immune Destruction
Genomic Instability & Mutation
Polymorphic Microbiome
Oncogenic Signaling
Damaging Mutations
Bypassing Growth Factor Activation
Adaptive Immune Response
Innate Immune Response
Methylation
DNMT3A
TET
Epigentic Gene Regulation
Therapeutic Index
Healthy Gut Microbiome
FMT
Cell Response
-DNA Damage
-Cell Proliferation
–Cell Survival
Tumor Promoting Inflammation
Immune Desert
Immune Exclusion
Oncogenic Activation
Neoantigen Presentation
Cancer Transformation
Prev
Next

## Slide 40
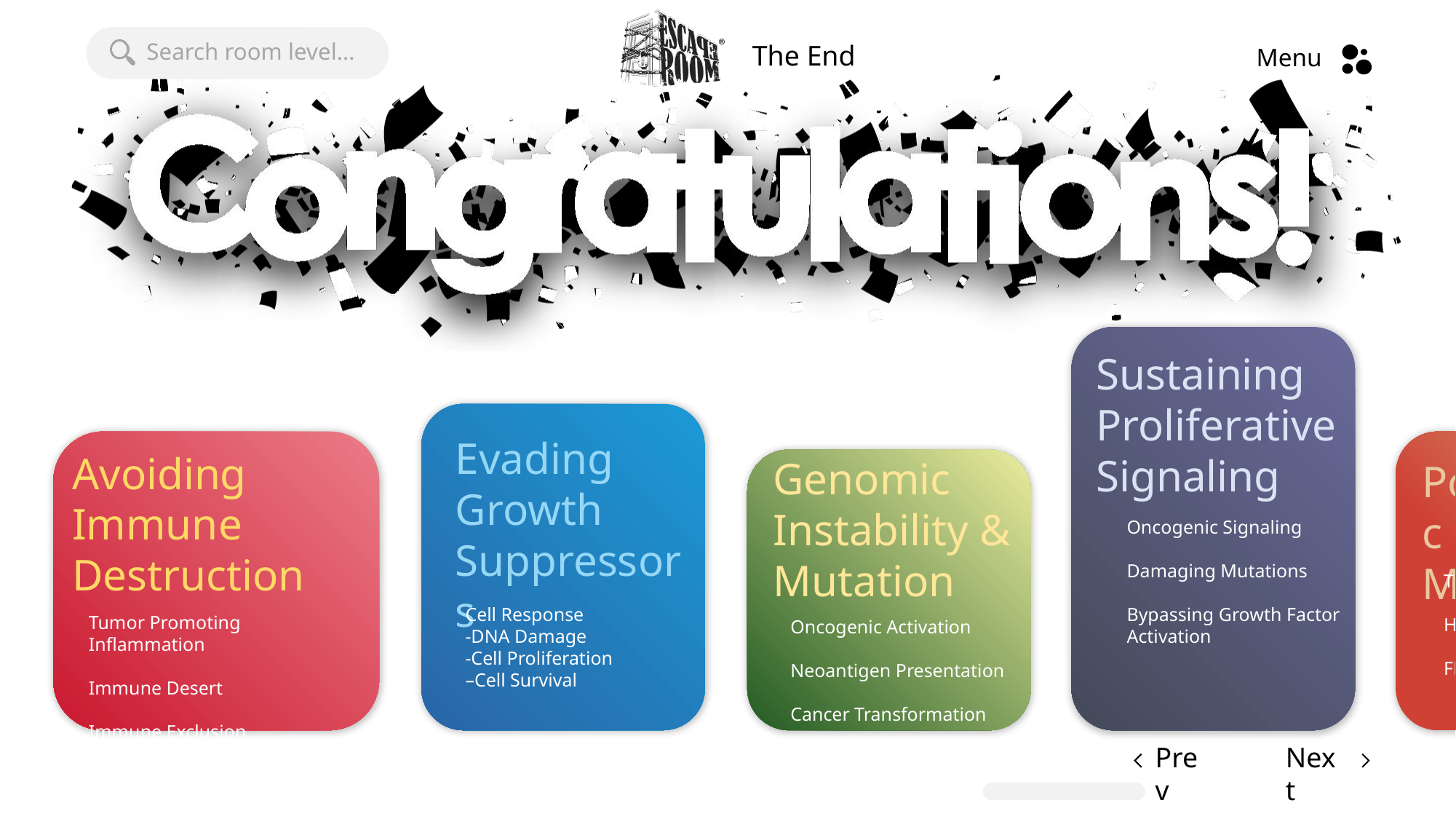

Search room level…
The End
Menu
Sustaining Proliferative Signaling
Avoiding Immune Destruction
Nonmutational Epigenetic Reprogramming
Evading Growth Suppressors
Avoiding Immune Destruction
Genomic Instability & Mutation
Polymorphic Microbiome
Oncogenic Signaling
Damaging Mutations
Bypassing Growth Factor Activation
Adaptive Immune Response
Innate Immune Response
Methylation
DNMT3A
TET
Epigentic Gene Regulation
Therapeutic Index
Healthy Gut Microbiome
FMT
Cell Response
-DNA Damage
-Cell Proliferation
–Cell Survival
Tumor Promoting Inflammation
Immune Desert
Immune Exclusion
Oncogenic Activation
Neoantigen Presentation
Cancer Transformation
Prev
Next

## Slide 41
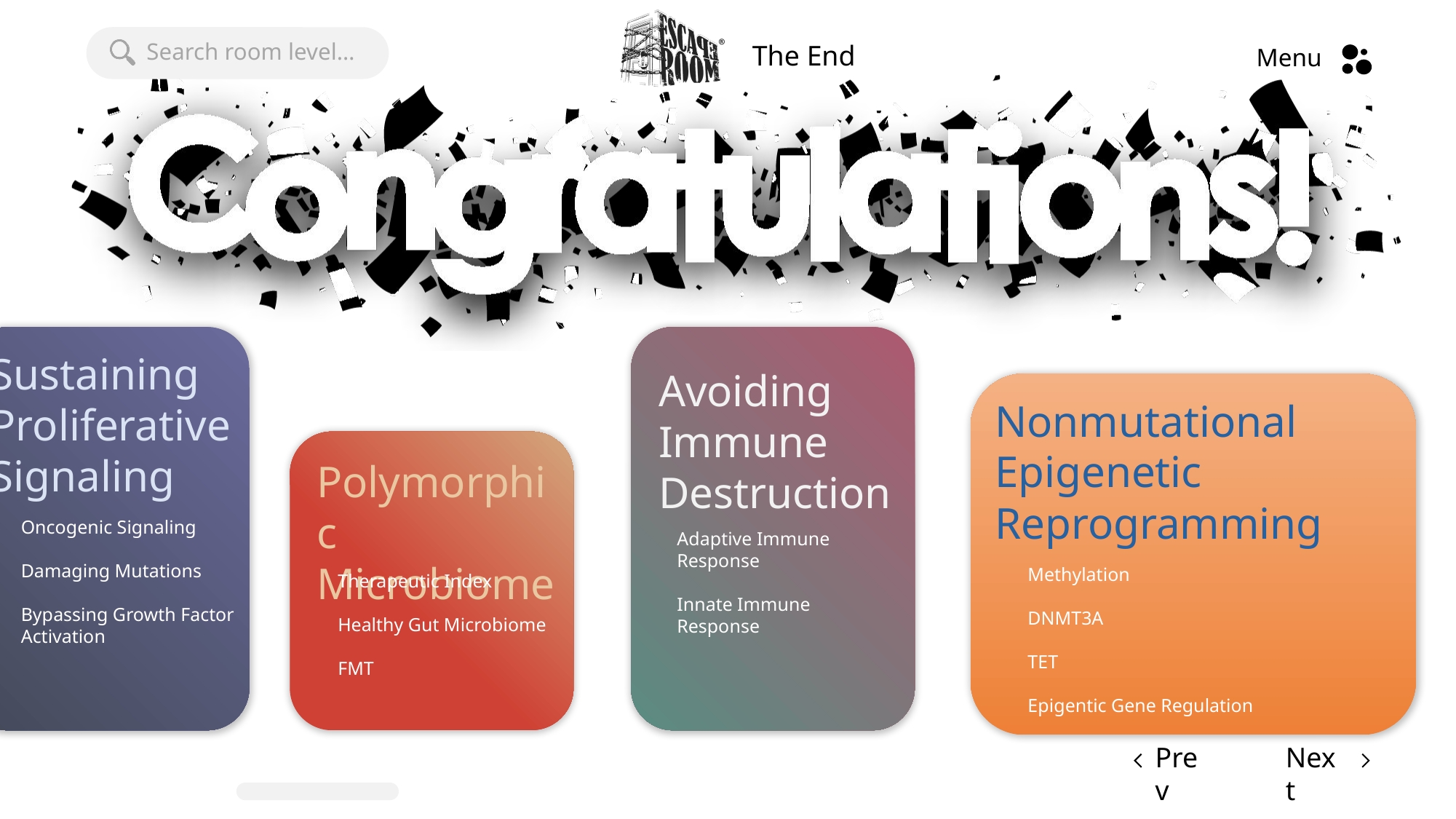

Search room level…
The End
Menu
Sustaining Proliferative Signaling
Avoiding Immune Destruction
Nonmutational Epigenetic Reprogramming
Evading Growth Suppressors
Avoiding Immune Destruction
Genomic Instability & Mutation
Polymorphic Microbiome
Oncogenic Signaling
Damaging Mutations
Bypassing Growth Factor Activation
Adaptive Immune Response
Innate Immune Response
Methylation
DNMT3A
TET
Epigentic Gene Regulation
Therapeutic Index
Healthy Gut Microbiome
FMT
Cell Response
-DNA Damage
-Cell Proliferation
–Cell Survival
Tumor Promoting Inflammation
Immune Desert
Immune Exclusion
Oncogenic Activation
Neoantigen Presentation
Cancer Transformation
Prev
Next
