# Supplementary material for: A summer course in cancer for high school students-an update on lessons taught and lessons learned
Source: BMC Med Educ. 2024 Sep 17;24:1020. doi: 10.1186/s12909-024-06002-z (PMC11409685; doi:10.1186/s12909-024-06002-z)
Supplement: Supplementary file 5 — Supplementary Material 5 [file 12909_2024_6002_MOESM5_ESM.docx]

**Extended Methods**

**Curriculum Modules and Content**

Module 1

Module 1 introduced the course’s foundation and reinforced critical biological concepts. These include “central dogma” and “mutation difference leading to disease.” For instance, chromatin translocation of the t (9;22) (q34; q11) (Philadelphia chromosome) comprising the ABL gene and the BCR gene, producing BCR-ABL oncogene is a famous fusion event driving chronic myeloid leukemia (CML). The learning objective was for students to investigate the factors and mechanisms contributing to cancer cell development and transformation, Table 1. The following lecture was to introduce students to all cancer hallmarks. We based this lecture on the recent updates to cancer hallmarks [8]. We expected students to explore the fundamental characteristics of cancer hallmarks and gain insights into how cancer cells behave differently from normal cells. The students were exposed to unfamiliar topics for the student-led group assignment and learned how to research and interpret data. For instance, students needed to research the molecular target for erlotinib (Tarceva) before analyzing the Kaplan Meier (KM) survival plot.

A critical hallmark of cancer is genomic instability and mutation. The second lecture focused on understanding what DNA mutations were and teaching students that multiple types of mutations can have varying effects depending on the gene mutated. Students were taught the difference between a Tumor Suppressor and an Oncogene, one of the fundamental lessons of cancer biology that will help set the stage for greater comprehension of the rest of the course. Two hallmark genes (p53 and KRAS) were discussed in depth to provide an example of one tumor suppressor and oncogene. Moreover, students engaged in two case studies covering cancer patient diagnoses & treatment to promote a deeper understanding of the topic. Each case study had a prompt that required students to think critically and engage with their peers to come up with an answer. The objective of the lecture and case study activity was to help students investigate the factors and mechanisms contributing to cancer cells’ development and transformation.

Module 2

Epigenetics is a less common but important concept in cancer. Thus, we wanted to introduce students to an unfamiliar topic. At the beginning of the lecture, students took a survey question to explain, “What makes a neuron different from a muscle cell?” The goal was to help students understand the importance of epigenetics in biology. The idea of epigenetics was explained, though the lecture only focused on DNA methylation for simplicity. The students were then asked to hypothesize the level of DNA methylation on a tumor suppressor gene and an oncogene to assess their comprehension. Following the lecture, the students were then tasked with two short case studies reflecting on actual situations in which DNA methylation was altered in cancer or during famine and had to hypothesize how DNA methylation could change in response to these events. One final case study was presented, in which the whole class had to collaborate to come up with possible treatment options if a patient had a mutation in an enzyme causing DNA methylation or in an enzyme removing DNA methylation. This discussion helped students fully understand the role of DNA methylation, the process of DNA methylation, and how disruption/mutations in this cycle could be relevant in cancer & how to target it for treatment.

Module 3

The overall learning objective for module 3 was to teach students that the immune system protects us against cancer, but when it fails, cancer can develop. Specifically, it was important for students to understand the relationship between the immune system and cancer and how immune responses can affect the progression of the disease. Important concepts covered in this lecture were the following: the science of immunology and examples of immunity, history of immunology (e.g., smallpox vaccine as an example of how vaccines work), development of immune cells and the immune response (e.g., innate and adaptive immune responses), immune system defenses against cancer, and why the immune system sometimes fails to protect us. The goal of the second lecture, “Immunotherapies for Cancer,” was to provide an understanding of how we can exploit our immune system to create therapies against cancer. Immunotherapy is a growing field that has already improved the standard of care for malignancies such as melanoma [12]. This lecture aimed to help students understand that immunotherapies may not be effective for all cancer patients and that different immunotherapies may be used to treat different cancer types. Concepts covered in this lecture were the tumor microenvironment and the role of immune cells within, neoantigens and mutations, immunosurveillance, the cancer immunity cycle, and types of immunotherapy and how they work against cancer (adoptive cell therapy, CAR T-cells, immune checkpoint inhibitors, and cancer vaccines).

Module 4

The incorporation of the “polymorphic microbiome” into the Hallmarks of Cancer [8] has illuminated its significant role in intersecting with genetic factors and inflammation for supporting cancer. This growing field presents exciting prospects for advancing our understanding and potentially harnessing the microbiome to enhance cancer prevention and treatment strategies. The primary objective of educating high school students about the microbiome’s role in cancer is to provide them with a foundational comprehension of how the diverse microorganisms within our bodies can significantly influence our overall health. It was crucial to emphasize that even a minor disruption in the gut microbiome can potentially contribute to the development and progression of cancer. Moreover, it endeavors to impart to them an awareness of the significant link between alterations in the microbiome and cancer, elucidating the mechanisms through which specific microorganisms can influence cancer risk. In pursuit of this goal, students engaged in interactive learning experiences, such as the Human Gut Game (HGG) (2), a group-based activity that simulates the complexities of the human gut microbiome, allowing them to explore how changes in microbial populations can affect health outcomes. This educational way encourages critical thinking and exploration of ongoing research, nurturing a sense of curiosity and a passion for lifelong learning in microbiome-cancer interactions while fostering communication skills. Furthermore, the course concluded with a lecture covering treatment strategies available in cancer clinics and the diverse approaches for modulating the microbiome as a potential foundation for treatment.

**Cooperative Learning Assignments- Case Studies, Presentation, and Escape Room**

Throughout our course, we introduced students to “mock” clinical case studies that revolved around crucial aspects of cancer research, intending to familiarize them with cooperative learning environments. In order to help students understand the relationship between cancer hallmarks and the clinical practicality of applied and translational sciences, the students were assigned into two groups. The first group presented the findings on the development, efficacy, safety, and culmination of a novel drug candidate for glioblastoma in clinical phase 4 trials. Specific instructions were to form a hypothesis proposing a potential drug target or approach to inhibit tumor growth or enhance treatment response, to include pre-clinical analysis (in vitro and in vivo), team collaboration (identify interdisciplinary teams for completion of the project), data analysis and interpretation, and to design a clinical trial from phase 1-3 (considering the ethical implications). The second group was tasked with demonstrating the journey of developing a novel diagnostic method for CML, from hypothesis formulation to its implementation in clinical practice. Specifically, they were to hypothesize a specific biomarker or genetic aberration that could serve as a reliable diagnostic marker for early detection of CML. Similarly, the case study must include pre-clinical analysis, team collaboration, data analysis and interpretation, and clinical trial design. These case studies served as a valuable platform for enhancing the students’ presentation and communication skills, fostering teamwork, and applying scientific principles to real-world scenarios. Their presentation encompassed essential elements within their case study. Their presentation adeptly highlighted the translational aspects of the research, bridging pre-clinical findings to clinical applications. Furthermore, they tackled challenges, outlined anticipated clinical outcomes, and thoughtfully addressed the potential for long-term effects, stressing the significance of continuous safety and efficacy monitoring.

The escape room activity fostered students with a comprehensive open-note assessment featuring seven challenging, multistep questions. Additionally, “eliminated” students engaged in a cancer hallmark worksheet covering lecture-discussed hallmarks in a multistep case study format. The escape room aimed to explore mechanisms involved in evading immune destruction, including concepts such as tumor immune profiles (immune inflamed, immune desert, and immune excluded), as well as strategies for evading growth suppressors, genomic instability and mutation, sustaining proliferative signaling, and the impact of the polymorphic microbiome. Emphasis was placed on understanding adaptive and innate immune responses and non-mutational epigenetic reprogramming.

**Panel Discussion**

At the end of the course, we also organized a career panel for the students to learn about the wide umbrella of opportunities within the STEM field. We included panelists from a wide range of STEM careers, representing diverse stages in their professional journeys:

- Biomedical Sciences PhD student in the final stage of their PhD
- MD/PhD student in their second year of PhD (2 years of medical school already done)
- Venture capitalist with experience in consulting (PhD in Biomedical Sciences)
- Bioinformatician (PhD in Biomedical Sciences)
- PhD student in Physics focusing on biophysical applications in cancer
- Postdoctoral student in Biomedical Engineering with a focus on CRISPR gene therapy
- Physician and Principal Investigator of a laboratory (MD)
- Radiation Oncology Physician Resident (MD)

First, the panelists introduced themselves, briefly describing their career trajectories. The intention behind this was to familiarize students with key steps and diverse strategies for entering STEM careers. After that, we opened the floor to ask the students questions. Some of the topics and questions that were discussed that can serve for future panel discussions are summarized below:

- Why did you become interested in your career?
- What steps did you take to get to where you are today in your career?
- What are the differences between medical school, graduate school (MS or PhD), and MD/PhD? How are the requirements different?
- Do you enjoy your current role?
- If you could start your career over, would you choose the same career path?
- What advice do you have for high school students planning to go to college and pursue a STEM career in the future?
